# Fisher Scoring for crossed factor linear mixed models

**DOI:** 10.1007/s11222-021-10026-6

**Published:** 2021-07-19

**Authors:** Thomas Maullin-Sapey, Thomas E. Nichols

**Affiliations:** Big Data Institute, Li Ka Shing Centre for Health Information and Discovery, Old Road Campus, Oxford, OX3 7LF UK

**Keywords:** Fisher Scoring, Linear mixed model, Crossed factors

## Abstract

The analysis of longitudinal, heterogeneous or unbalanced clustered data is of primary importance to a wide range of applications. The linear mixed model (LMM) is a popular and flexible extension of the linear model specifically designed for such purposes. Historically, a large proportion of material published on the LMM concerns the application of popular numerical optimization algorithms, such as Newton–Raphson, Fisher Scoring and expectation maximization to single-factor LMMs (i.e. LMMs that only contain one “factor” by which observations are grouped). However, in recent years, the focus of the LMM literature has moved towards the development of estimation and inference methods for more complex, multi-factored designs. In this paper, we present and derive new expressions for the extension of an algorithm classically used for single-factor LMM parameter estimation, Fisher Scoring, to multiple, crossed-factor designs. Through simulation and real data examples, we compare five variants of the Fisher Scoring algorithm with one another, as well as against a baseline established by the R package lme4, and find evidence of correctness and strong computational efficiency for four of the five proposed approaches. Additionally, we provide a new method for LMM Satterthwaite degrees of freedom estimation based on analytical results, which does not require iterative gradient estimation. Via simulation, we find that this approach produces estimates with both lower bias and lower variance than the existing methods.

**Supplementary Information:**

The online version supplementary material available at 10.1007/s11222-021-10026-6.

## Introduction

### Background

Since its conception in the seminal work of Laird and Ware (1982), the literature on linear mixed model (LMM) estimation and inference has evolved rapidly. At present, many software packages exist which are capable of performing LMM estimation and inference for large and complex LMMs in an incredibly quick and memory-efficient manner. For some packages, this exceptional speed and efficiency arise from simplifying model assumptions, while for others, complex mathematical operations such as sparse matrix methodology and sweep operators are utilized to improve performance (Wolfinger et al. 1994; Bates et al. 2015).

However, due to practical implementation concerns, the current methodology cannot be applied in certain situations. For example, in the mass-univariate analyses used in medical imaging, standard practice involves estimating hundreds of thousands of models concurrently. To efficiently perform a mass-univariate analysis within a practical time-frame, the use of vectorized computation which exploits the repetitive nature of simplistic operations to streamline calculation must be employed (Smith and Nichols 2018; Li et al. 2019). Unfortunately, many existing LMM tools utilize complex operations, for which vectorized support does not currently exist. As a result, alternative methodology, using more conceptually simplistic mathematical operations for which vectorized support exists, is required.

The complex nature of LMM computation has partly arisen from the gradual expansion of the definition of “linear mixed model”. Previously, the term was primarily used to refer to an extension of the linear regression model containing random effects grouped by a single random factor. Examples of this definition can be seen in Laird and Ware (1982), in which “linear mixed model” refers only to “single-factor” longitudinal models, and in Lindstrom and Bates (1988), where more complex, multi-factor models are described as an “extension” of the “linear mixed model”. Consequently, throughout the late 1970s and 1980s, one of the main focuses of the LMM literature was to provide parameter estimation methods such as Fisher Scoring, Newton–Raphson and expectation maximization for the single-factor LMM (e.g. Dempster et al. 1977; Jennrich and Schluchter 1986; Laird et al. 1987). By exploiting structural features of the single-factor model, implementation of these methods required only conceptually simplistic mathematical operations. For instance, the Fisher Scoring algorithm proposed by Jennrich and Schluchter (1986) relies only upon vector addition and matrix multiplication, inversion, and reshaping operations.

More recently, usage of the term “linear mixed models” has grown substantially to include models which contain random effects grouped by multiple random factors. Examples of this more general definition are found in Pinheiro and Bates (2009) and Tibaldi et al. (2007). For this more general “multi-factor” LMM definition, models can be described as exhibiting either a hierarchical factor structure (i.e. factor groupings are nested inside one another) or crossed factor structure (i.e. factor groupings are not nested inside one another). For instance, a study involving students grouped by the factors “school” and “class” contains hierarchical factors (as every class belongs to a specific school). In contrast, a study involving observations grouped by “subject” and “location”, where subjects change location between recorded measurements, contains crossed factors (as each subject is not generally associated with a specific location or vice versa). In either case, the computational approaches used in the single-factor setting cannot be directly applied to the more complex multi-factor model. For this reason, parameter estimation of the multi-factor LMM has often been viewed as a much more “difficult” problem than its single-factor counterpart (see, for example, the discussion in chapters 2 and 8 of West et al. 2014).

Several authors have proposed and implemented methods for multi-factor LMM estimation. However, such methods typically require conceptually complex mathematical operations, which are not naturally amenable to vectorized computation, or restrictive simplifying model assumptions, which prevent some designs from being estimated. For example, the popular R package lme4 performs LMM estimation via minimization of a penalized least squares cost function based on a variant of Henderson’s mixed model equations (Henderson et al. 1959; Bates et al. 2015). However, sparse matrix methods are required to achieve fast evaluation of the cost function, and advanced numerical approximation methods are needed for optimization (e.g. the Bound Optimization BY Quadratic Approximation, BOBYQA, algorithm, Powell 2009). The commonly used SAS and SPSS packages, PROC-MIXED and MIXED, employ a Newton–Raphson algorithm proposed by Wolfinger et al. (1994). In this approach, though, derivatives and Hessians must be computed using the sweep operator, W-transformation, and Cholesky decomposition updating methodology (SAS Institute Inc 2015; IBM Corp 2015). The hierarchical linear models (HLM) package takes an alternative option and restricts its inputs to only LMMs which contain hierarchical factors (Raudenbush and Bryk 2002), although the hierarchical cross-classified models (HCM) sub-module does make allowances for specific use-case crossed LMMs. To perform parameter estimation, HLM employs a range of different methods, each tailored to a particular model design. As a result, there are many models for which HLM does not provide support.

Methods have also been proposed for evaluating the score vector (the derivative of the log-likelihood function) and the Fisher Information matrix (the expected Hessian of the negative log-likelihood function) required to perform Fisher Scoring for the multi-factor LMM. For example, alongside the Newton–Raphson approach adopted by PROC-MIXED and MIXED, Wolfinger et al. (1994) also describe a Fisher Scoring algorithm. However, evaluation of the expressions they provide requires the use of the sweep operator, W-transformation, and Cholesky decomposition updating methodology, operations for which widespread vectorized support do not yet exist. More recently, expressions for score vectors and Fisher Information matrices were provided by Zhu and Wathen (2018). However, the approach (Zhu and Wathen 2018) adopt to derive these expressions produces an algorithm that requires independent computation for each variance parameter in the LMM. This algorithm’s serialized nature results in substantial overheads in terms of computation time, thus limiting the method’s utility in practical situations where time-efficiency is a crucial consideration.

To our knowledge, no approach has yet been provided for multi-factor LMM parameter estimation, which utilizes only simplistic, universally available operations which are naturally amenable to vectorized computation. In this work, we revisit a Fisher Scoring approach suggested for the single-factor LMM, described in Demidenko (2013), extending it to the multi-factor setting, with the intention of revisiting the motivating example of the mass-univariate model in later work.

The novel contribution of this work is to provide new derivations and closed-form expressions for the score vector and Fisher Information matrix of the multi-factor LMM. We show how these expressions can be employed for LMM parameter estimation via the Fisher Scoring algorithm and can be further adapted for constrained optimization of the random effects covariance matrix. Additionally, we demonstrate that such closed-form expressions are also of use in the setting of mixed model inference, where the degrees of freedom of the approximate *T*-statistic are not known and must be estimated (Verbeke and Molenberghs 2001). We show how our derived results may be combined with the Satterthwaite-based method for approximating degrees of freedom for the LMM by using an approach based on the work of Kuznetsova et al. (2017).

In this paper, we first propose five variants of the Fisher Scoring algorithm. Following this, we provide a discussion of initial starting values for the algorithm and methods for improving the algorithm’s computational efficiency during implementation. Detail on constrained optimization, allowing for structural assumptions to be placed on the random effects covariance matrix, is then provided. Proceeding this, new expressions for Satterthwaite estimation of the degrees of freedom of the approximate *T*-statistic for the multi-factor LMM are given. Finally, we verify the correctness of the proposed algorithms and degrees of freedom estimates via simulation and real data examples, benchmarking the performance against the R package lme4.

### Preliminaries

#### The model

Both the single-factor and multi-factor LMM, for *n* observations, take the following form:

1 $$\begin{aligned} \begin{aligned}&Y=X\beta + Zb + \epsilon \\&\epsilon \sim N(0,\sigma ^2 I_n), \quad b \sim N(0, \sigma ^2 D), \\ \end{aligned} \end{aligned}$$

The known quantities in the model are; *Y* (the $(n \times 1)$ vector of responses), *X* (the $(n \times p)$ fixed effects design matrix) and *Z* (the $(n \times q)$ random effects design matrix). The unknown model parameters are: $\beta$ (the $(p \times 1)$ fixed effects parameter vector), $\sigma ^2$ (the scalar fixed effects variance) and *D* (the $(q \times q)$ random effects covariance matrix). From (1), the marginal distribution of the response vector, *Y*, can be seen to be $N(X\beta ,\sigma ^2 (I_n + ZDZ'))$. The log-likelihood for the LMM specified by (1) is derived from the marginal distribution of *Y*. Dropping constant terms, this log-likelihood is given by:

2 $$\begin{aligned} l(\theta )=-\frac{1}{2}\bigg \{ n\log (\sigma ^2)+\sigma ^{-2}e'V^{-1}e+\log |V|\bigg \}, \end{aligned}$$

where $\theta$ is shorthand for all the parameters $(\beta , \sigma ^2, D)$, $V=I_n+ZDZ'$ and $e=Y-X\beta$. Throughout the main body of this work, we shall consider parameter estimation performed via maximum likelihood (ML) estimation of (2). However, we note that the approaches we describe for ML estimation can also be easily adapted to use a restricted maximum likelihood (ReML) criterion. Further detail on ReML estimation is provided in Appendix 6.3.

The distinction between the multi-factor and single-factor LMMs lies in the specification of the random effects in the model. Random effects are often described in terms of factors, categorical variables that group the random effects, and levels, individual instances of such a categorical variable. We highlight here that the term “factor”, in this work, refers only to categorical variables which group random effects and does not refer to groupings of fixed effects. We denote the total number of factors in the model as *r* and denote the *k*th factor in the model as $f_k$ for $k \in \{1,\ldots ,r\}$. For a given factor $f_k$, $l_k$ will be used to denote the number of levels possessed by $f_k$, and $q_k$ the number of random effects which $f_k$ groups. The single-factor LMM corresponds to the case $r=1$, while the multi-factor setting corresponds to the case $r>1$.

An example of how this notation may be used in practice is given as follows. Suppose an LMM contains observations that are grouped by “subject” (i.e. the participant whose observation was recorded) and “location” (i.e. the place the observation was recorded). Further, suppose that the LMM includes a random intercept and random slope which each model subject-specific behaviour, and a random intercept which models location-specific behaviour. Two factors are present in this design: the factor $f_1$ is “subject” and the factor $f_2$ is “location”. Therefore, $r=2$. The number of subjects is $l_1$ and the number of locations is $l_2$. The number of covariates grouped by the first factor, $q_1$, is 2 (i.e. the random intercept and the random slope) and the number grouped by the second factor, $q_2$, is 1 (i.e. the random intercept).

The values of *r*, $\{q_k\}_{k\in \{1,\ldots ,r\}}$ and $\{l_k\}_{k\in \{1,\ldots ,r\}}$ determine the structure of the random effects design matrix, *Z*, and random effects covariance matrix, *D*. To specify *Z* formally is notationally cumbersome and of little relevance to the aims of this work. For this reason, the reader is referred to the work of Bates et al. (2015) for further detail on the construction of *Z*. Here, it suffices to note that, under the assumption that its columns are appropriately ordered, *Z* is comprised of *r* horizontally concatenated blocks. The *k*th block of *Z*, denoted $Z_{(k)}$, has dimension $(n \times l_kq_k)$ and describes the random effects which are grouped by the *k*th factor. Additionally, each block, $Z_{(k)}$, can be further partitioned column-wise into $l_k$ blocks of dimension $(n \times q_k)$. The *j*th block of $Z_{(k)}$, denoted $Z_{(k,j)}$, corresponds to the random effects which belong to the *j*th level of the *k*th factor. In summary,

$$\begin{aligned} \begin{aligned}&Z = [Z_{(1)},Z_{(2)},\ldots Z_{(r)}], \\&Z_{(k)} = [Z_{(k,1)},Z_{(k,2)},\ldots Z_{(k,l_k)}] \quad \text {(for }k\in \{1,\ldots , r\}{)} \end{aligned} \end{aligned}$$

An important property of the matrix *Z* is that for any arbitrary factor $f_k$, the rows of $Z_{(k)}$ can be permuted in order to obtain a block diagonal matrix. As, for the single-factor LMM, $Z\equiv Z_{(1)}$, it follows that the observations of the single-factor LMM can be arranged such that *Z* is block diagonal. This feature of the single-factor LMM simplifies the derivation of the Fisher Information matrix and score vector required for Fisher Scoring. However, this simplification cannot be generalized to the multi-factor LMM. In general, it is not true that the rows of *Z* can be permuted in such a way that the resultant matrix is block-diagonal. As emphasized in Sect. 1.1, due to this, many of the results derived in the single-factor LMM have not been generalized to the multi-factor setting.

To describe the random effects covariance matrix, *D*, it is assumed that factors are independent from one another and that for each factor, factor $f_k$, there is a $(q_k \times q_k)$ unknown covariance matrix, $D_k$, representing the “within-factor” covariance for the random effects grouped by $f_k$. The random effects covariance matrix, *D*, appearing in (1), can now be given as $D= \bigoplus _{k=1}^r (I_{l_k} \otimes D_k)$ where $\oplus$ represents the direct sum, and $\otimes$ the Kronecker product. Note that, while *D* is large in dimension (having dimension $(q \times q)$ where $q=\sum _i q_il_i$), *D* contains $q_u =\sum _i\frac{1}{2}q_i(q_i+1)$ unique elements. Typically, it is true that $q_u<<q^2$. As a result, the task of describing the random effects covariance matrix *D* reduces in practice to specifying only a small number of parameters.

#### Notation

In this section, we introduce notation which will be used throughout the remainder of this work. The hat operator is used to denote estimators resulting from likelihood maximisation procedures (e.g. $\beta$ represents the true fixed effects parameter vector while the maximum likelihood estimate of $\beta$ is denoted ${\hat{\beta }}$). Subscript notation, $A_{[X,Y]}$, is used to denote the sub-matrix of matrix *A*, composed of all elements of *A* with row indices $x \in X$ and column indices $y \in Y$. The replacement of *X* or *Y* with a colon,  : , represents all rows or all columns of *A*, respectively. If a scalar, *x* or *y*, replaces *X* or *Y*, this represents the elements with row indices $x=X$ or column indices $y=Y$, respectively. The notation (*k*) may also replace *X* and *Y* where (*k*) represents the indices of the columns of *Z* which correspond to factor $f_k$. Similarly, *X* and *Y* may be substituted for the ordered pair (*k*, *j*) where (*k*, *j*) represents the indices of the columns of *Z* which correspond to level *j* of factor $f_k$. We highlight again our earlier notation, $Z_{(k,j)}$, which, due to its frequent occurrence acts as a shorthand for $Z_{[:,(k,j)]}$, i.e. the columns of *Z* corresponding to level *j* of factor $f_k$.

Finally, we shall also adopt the notations “vec”, “vech”, $N_k$, $K_{m,n}$, ${\mathcal {D}}_k$ and ${\mathcal {L}}_k$ as used in Magnus and Neudecker (1980), defined as follows:

- “vec” represents the mathematical vectorization operator which transforms an arbitrary $(k \times k)$ matrix, *A*, to a $(k^2 \times 1)$ column vector, vec(*A*), composed of the columns of *A* stacked into a column vector. (Note: this is different to the concept of computational vectorization discussed in Sect. 1.1).
- “vech” represents the half-vectorization operator which transforms an arbitrary square matrix, *A*, of dimension $(k \times k)$ to a $(k(k+1)/2 \times 1)$ column vector, vech(*A*), composed by stacking the elements of *A* which fall on and below the diagonal into a column vector.
- $N_k$ is defined as the unique matrix of dimension $(k^2 \times k^2)$ which implements symmetrization for any arbitrary square matrix *A* of dimension $(k \times k)$ in vectorized form, i.e. $N_k$ satisfies the following relation:
  $$\begin{aligned} N_k\text {vec}(A)=\text {vec}(A+A')/2. \end{aligned}$$
- $K_{m,n}$ is the unique “Commutation” matrix of dimension $(mn \times mn)$, which permutes, for any arbitrary matrix *A* of dimension $(m \times n)$, the vectorization of *A* to obtain the vectorization of the transpose of *A*, i.e. $K_{m,n}$ satisfies the following relation:
  $$\begin{aligned} \text {vec}(A)=K_{m,n}\text {vec}(A'). \end{aligned}$$
- ${\mathcal {D}}_k$ is the unique “Duplication” matrix of dimension $(k^2 \times k(k+1)/2)$, which maps the half-vectorization of any arbitrary symmetric matrix *A* of dimension $(k \times k)$ to its vectorization, i.e. ${\mathcal {D}}_k$ satisfies the following relation:
  $$\begin{aligned} \text {vec}(A)={\mathcal {D}}_k\text {vech}(A). \end{aligned}$$
- ${\mathcal {L}}_k$ is the unique $1-0$ “elimination” matrix of dimension $(k(k+1)/2 \times k^2)$, which maps the vectorization of any arbitrary lower triangular matrix *A* of dimension $(k \times k)$ to its half-vectorization, i.e. ${\mathcal {L}}_k$ satisfies the following relation:
  $$\begin{aligned} \text {vech}(A)={\mathcal {L}}_k\text {vec}(A). \end{aligned}$$

To help track the notational conventions employed in this work, an index of notation is provided in the Supplementary Material Section S10.

## Methods

### Fisher Scoring algorithms

In this section, we employ the Fisher Scoring algorithm for ML estimation of the parameters $(\beta , \sigma ^2, D)$. The Fisher Scoring algorithm update rule takes the following form:

3 $$\begin{aligned} \theta _{s+1} = \theta _{s} + \alpha _s{\mathcal {I}}(\theta _{s})^{-1}\frac{dl(\theta _s)}{d\theta }_, \end{aligned}$$

where $\theta _s$ is the vector of parameter estimates given at iteration *s*, $\alpha _s$ is a scalar step size, the score vector of $\theta _s$, $\frac{dl(\theta _s)}{d\theta }$, is the derivative of the log-likelihood with respect to $\theta$ evaluated at $\theta =\theta _s$, and ${\mathcal {I}}(\theta _{s})$ is the Fisher Information matrix of $\theta _s$;

$$\begin{aligned} {\mathcal {I}}(\theta _{s})=E\bigg [\bigg (\frac{dl(\theta )}{d \theta }\bigg )\bigg (\frac{dl(\theta )}{d \theta }\bigg )'\bigg |\theta =\theta _s\bigg ]. \end{aligned}$$

A more general formulation of Fisher Scoring, which allows for low-rank Fisher Information matrices, is given by Rao and Mitra (1972):

4 $$\begin{aligned} \theta _{s+1} = \theta _{s} + \alpha _s{\mathcal {I}}(\theta _{s})^{+}\frac{dl(\theta _s)}{d\theta }_, \end{aligned}$$

where superscript plus, $^+$, is the Moore–Penrose (or “pseudo”) inverse. For notational brevity, when discussing algorithms of the form (3) and (4) in the following sections, the subscript *s*, representing iteration number, will be suppressed unless its inclusion is necessary for clarity.

For the LMM, several different representations of the parameters of interest, $(\beta , \sigma ^2,D)$, can be used for numerical optimization and result in different Fisher Scoring iteration schemes. In this section, we consider the following three representations for $\theta$:

$$\begin{aligned} \theta ^h = \begin{bmatrix}\beta \\ \sigma ^2\\ \text {vech}(D_1)\\ \vdots \\ \text {vech}(D_r)\\ \end{bmatrix}, \theta ^f = \begin{bmatrix}\beta \\ \sigma ^2\\ \text {vec}(D_1)\\ \vdots \\ \text {vec}(D_r)\\ \end{bmatrix}, \theta ^c = \begin{bmatrix}\beta \\ \sigma ^2\\ \text {vech}(\Lambda _1)\\ \vdots \\ \text {vech}(\Lambda _r)\\ \end{bmatrix}, \end{aligned}$$

where $\Lambda _k$ represents the lower triangular Cholesky factor of $D_k$, such that $D_k=\Lambda _k\Lambda _k'$. We will refer to the representations ($\theta ^h$, $\theta ^f$ and $\theta ^c$) as the “half”, “full” and “Cholesky” representations of $(\beta , \sigma ^2, D)$, respectively. In a slight abuse of notation, the function *l* will be allowed to take any representation of $\theta$ as input, with the interpretation unchanged (i.e. $l(\theta ^f)=l(\theta ^h)=l(\theta ^c)$). For example, if the full representation is being used, the log-likelihood will be denoted $l(\theta ^f)$, but if the half representation is being used, the log likelihood will be denoted $l(\theta ^h)$.

In the following sections, the score vectors and Fisher Information matrices required to perform five variants of Fisher Scoring for the multi-factor LMM will be stated with proofs provided in Appendices 6.1 and 6.2. For notational convenience, we denote the sub-matrix of the Fisher Information matrix of $\theta ^{h}$ with row indices corresponding to parameter vector *a* and column indices corresponding to parameter vector *b* as ${\mathcal {I}}^{h}_{a,b}$. In other words, ${\mathcal {I}}^{h}_{a,b}$ is the sub-matrix of ${\mathcal {I}}(\theta ^{h})$, defined by:

$$\begin{aligned} {\mathcal {I}}^{h}_{a,b}=E\bigg [\bigg (\frac{dl(\theta ^{h})}{d a}\bigg )\bigg (\frac{dl(\theta ^{h})}{d b}\bigg )'\bigg |\theta =\theta _s\bigg ]. \end{aligned}$$

For further simplification of notation, when *a* and *b* are equal, the second subscript will be dropped and the matrix ${\mathcal {I}}^{h}_{a,b}={\mathcal {I}}^{h}_{a,a}$ will be denoted simply as ${\mathcal {I}}^{h}_{a}$. Analogous notation is used for the full and Cholesky representations.

#### Fisher Scoring

The first variant of Fisher Scoring we provide uses the “half”-representation for $(\beta , \sigma ^2, D)$, $\theta ^h$, and is based on the standard form of Fisher Scoring given by (3). This may be considered the most natural approach for a Fisher Scoring algorithm as $\theta ^h$ is an unmodified vector of the unique parameters of the LMM and (3) is the standard update rule. For this approach, the elements of the score vector are:

5 $$\begin{aligned}&\frac{d l(\theta ^h)}{d \beta } = \sigma ^{-2}X'V^{-1}e, \end{aligned}$$

6 $$\begin{aligned}&\frac{d l(\theta ^h)}{d \sigma ^2} = -\frac{n}{2}\sigma ^{-2}+\frac{1}{2}\sigma ^{-4}e'V^{-1}e. \end{aligned}$$

For $$k \in \{1,\ldots , r\}$$:

7 $$\begin{aligned}&\frac{d l(\theta ^h)}{d \text {vech}(D_k)} =\nonumber \\&\quad \frac{1}{2}{\mathcal {D}}_{q_k}'\text {vec}\bigg ( \sum _{j=1}^{l_k}Z'_{(k,j)}V^{-1}\bigg (\frac{ee'}{\sigma ^2}-V\bigg )V^{-1}Z_{(k,j)}\bigg ). \end{aligned}$$

and the entries of the Fisher Information matrix are given by:

8 $$\begin{aligned} {\mathcal {I}}^h_{\beta }=\sigma ^{-2}X'V^{-1}X,\quad {\mathcal {I}}^h_{\beta ,\sigma ^2}={\mathbf {0}}_{p,1},\quad {\mathcal {I}}^h_{\sigma ^2}=\frac{n}{2}\sigma ^{-4}. \end{aligned}$$

For $$k \in \{1,\ldots , r\}$$:

9 $$\begin{aligned} \begin{aligned}&{\mathcal {I}}^h_{\beta ,\text {vech}(D_k)}={\mathbf {0}}_{p,q_k(q_k+1)/2},\\&{\mathcal {I}}^h_{\sigma ^2,\text {vech}(D_k)}= \frac{1}{2}\sigma ^{-2}\text {vec}'\bigg (\sum _{j=1}^{l_k} Z_{(k,j)}'V^{-1}Z_{(k,j)}\bigg ){\mathcal {D}}_{q_k}. \\ \end{aligned} \end{aligned}$$

For $$k_1,k_2\in \{1,\ldots ,r\}$$:

10 $$\begin{aligned} \begin{aligned}&{\mathcal {I}}^h_{\text {vech}(D_{k_1}),\text {vech}(D_{k_2})}\\&\quad =\frac{1}{2}{\mathcal {D}}'_{q_{k_1}}\sum _{j=1}^{l_{k_2}}\sum _{i=1}^{l_{k_1}}(Z'_{(k_1,i)}V^{-1}Z_{(k_2,j)}\otimes Z'_{(k_1,i)}V^{-1}Z_{(k_2,j)}){\mathcal {D}}_{q_{k_2}}. \end{aligned}\nonumber \\ \end{aligned}$$

where ${\mathbf {0}}_{n,k}$ denotes the $(n \times k)$-dimensional matrix of zeros. Due to its to natural approach to Fisher Scoring, this algorithm is referred to as FS in the remainder of this text. Pseudocode for the FS algorithm is given in Algorithm 1. Discussion of the initial estimates used in the algorithm is deferred to Sect. 2.2. To ensure *D* is non-negative definite, a commonly employed Eigendecomposition-based approach is used (c.f. Supplementary Material Section S11).
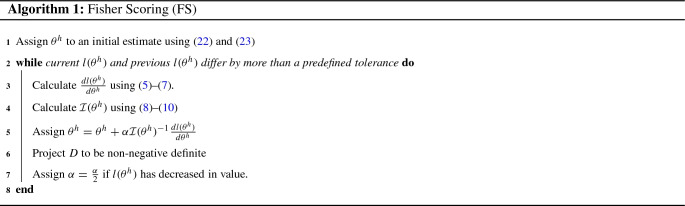


#### Full Fisher Scoring

The second variant of Fisher Scoring considered in this work uses the “full”, $\theta ^f$, representation of the model parameters, and shall therefore be referred to as “Full Fisher Scoring” (FFS). In this approach, for each factor, $f_k$, the elements of vec$(D_k)$ are to be treated as distinct from one another with numerical optimization for $D_k$ performed over the space of all $(q_k \times q_k)$ matrices. This approach differs from the FS method proposed in the previous section, in which optimization was performed on the space of only those $(q_k \times q_k)$ matrices that are symmetric. This optimization procedure is realized by treating symmetric matrix elements of $D_k$ as distinct and, for a given element, using the partial derivative with respect to the element during optimization instead of the total derivative with respect to both the element and its symmetric counterpart. This change is reflected by denoting the elements of the score vector which correspond to vec$(D_k)$ using a partial derivative operator, $\partial$, rather than the total derivative operator, *d*. The primary motivation for the inclusion of the FFS approach is that it serves as a basis for which the constrained covariance approaches of Sect. 2.4 can be built upon. However, it should be noted that as it does not require the construction or use of duplication matrices, FFS also provides simplified expressions and potential improvement in terms of computation speed. As a result, FFS is of some theoretical and practical interest and is detailed in full here.

An immediate obstacle to this approach is that the Fisher Information matrix of $\theta ^f$ is rank-deficient and, therefore, cannot be inverted. Intuitively, this is to be expected, as repeated entries in $\theta ^f$ result in repeated rows in ${\mathcal {I}}(\theta ^f)$. Mathematically, this can be seen by noting that ${\mathcal {I}}^f_{\text {vec}(D_{k})}$ can be expressed as a product containing the matrix $N_{q_k}$ (defined in Sect. 1.2.2), which is low-rank by construction. Consequently, the Fisher Scoring update rule for $\theta ^f$ must be based on the pseudo-inverse formulation of Fisher Scoring given in (4).

As the derivatives of the log-likelihood with respect to $\beta$ and $\sigma ^2$ do not depend upon the parameterisation of *D*, both FFS and FS employ the same expressions for the elements of the score vector which correspond to $\beta$ and $\sigma ^2$, given by (5) and (6), respectively. The score vector for $\{$vec($D_k)\}_{k\in \{1,\dots r\}}$ used by FFS is given by:

11 $$\begin{aligned} \frac{\partial l(\theta ^f)}{\partial \text {vec}(D_k)} {=} \frac{1}{2}\text {vec}\bigg ( \sum _{j=1}^{l_k}Z'_{(k,j)}V^{-1}\bigg (\frac{ee'}{\sigma ^2}-V\bigg )V^{-1}Z_{(k,j)}\bigg ).\nonumber \\ \end{aligned}$$

The entries of the Fisher Information matrix of $\theta ^f$, based on the derivatives given in (5), (6) and (11), are given by:

$$\begin{aligned} {\mathcal {I}}^f_{\beta }={\mathcal {I}}^h_{\beta }, \quad {\mathcal {I}}^f_{\beta ,\sigma ^2}={\mathcal {I}}^h_{\beta ,\sigma ^2},\quad {\mathcal {I}}^f_{\sigma ^2}={\mathcal {I}}^h_{\sigma ^2}. \end{aligned}$$

For $$k \in \{1,\ldots , r\}$$:

12 $$\begin{aligned} \begin{aligned}&{\mathcal {I}}^f_{\beta ,\text {vec}(D_k)}={\mathbf {0}}_{p,q_k^2},\\&{\mathcal {I}}^f_{\sigma ^2,\text {vec}(D_k)}= \frac{1}{2}\sigma ^{-2}\text {vec}'\bigg (\sum _{j=1}^{l_k} Z_{(k,j)}'V^{-1}Z_{(k,j)}\bigg ). \end{aligned} \end{aligned}$$

For $$k_1,k_2\in \{1,\ldots ,r\}$$:

13 $$\begin{aligned} \begin{aligned}&{\mathcal {I}}^f_{\text {vec}(D_{k_1}),\text {vec}(D_{k_2})}\\&\qquad =\frac{1}{2}\sum _{j=1}^{l_{k_2}}\sum _{i=1}^{l_{k_1}}(Z'_{(k_1,i)}V^{-1}Z_{(k_2,j)}\otimes Z'_{(k_1,i)}V^{-1}Z_{(k_2,j)})N_{q_k}. \end{aligned} \end{aligned}$$

Derivations for the above can be found in Appendices 6.1 and 6.2. It can also be seen that the Full Fisher Scoring algorithm can also be expressed in the form:

14 $$\begin{aligned} \theta ^f_{s+1} = \theta ^f_{s} + \alpha _s F(\theta _{s}^f)^{-1}\frac{\partial l(\theta _s^f)}{\partial \theta }_, \end{aligned}$$

where, unlike in (3) and (4), $F(\theta ^f)$ is not the Fisher Information matrix. Rather, $F(\theta ^f)$ is a matrix of equal dimensions to ${\mathcal {I}}(\theta ^f)$ with all of its elements equal to those of ${\mathcal {I}}(\theta ^f)$, apart from those which were specified by (13), which instead are, for $k_1,k_2\in \{1,\ldots ,r\}$:

15 $$\begin{aligned} \begin{aligned}&F_{\text {vec}(D_{k_1}),\text {vec}(D_{k_2})}\\&\quad =\frac{1}{2}\sum _{j=1}^{l_{k_2}}\sum _{i=1}^{l_{k_1}}(Z'_{(k_1,i)}V^{-1}Z_{(k_2,j)}\otimes Z'_{(k_1,i)}V^{-1}Z_{(k_2,j)}) \end{aligned}, \end{aligned}$$

where the same subscript notation has been adopted to index $F(\theta ^f)$ as was adopted for ${\mathcal {I}}(\theta ^f)$. This alternative representation of the FFS algorithm can be derived directly using well-known properties of the commutation matrix (c.f. Supplementary Material Section S13). Pseudocode for the FFS algorithm using the representation of the update rule given by (14) is provided by Algorithm 2.
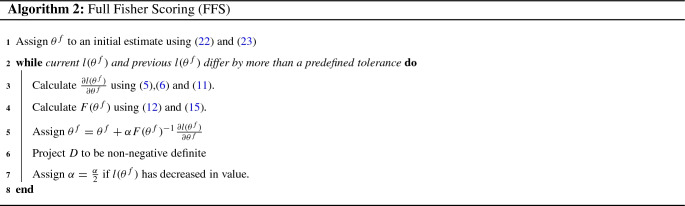


#### Simplified Fisher Scoring

The third Fisher Scoring algorithm proposed in this work relies on the half-representation of the parameters $(\beta ,\sigma ^2,D)$ and takes an approach, similar to that of coordinate ascent, which is commonly adopted in the single-factor setting (c.f. Demidenko 2013). In this approach, instead of performing a single update step upon the entire vector $\theta ^h$ in the form of (3), updates for $\beta , \sigma ^2$ and $\{D_k\}_{k \in \{1,\ldots , r\}}$ are performed individually in turn. For $\beta$ and $\sigma ^2$, each iteration uses the standard generalized least squares (GLS) estimators given by:

16 $$\begin{aligned} \beta _{s+1} = (X'V_s^{-1}X)^{-1}X'V_s^{-1}Y,\quad \sigma ^2_{s+1} = \frac{e_{s+1}'V^{-1}_{s}e_{s+1}}{n}.\nonumber \\ \end{aligned}$$

To update the random effects covariance matrix, $D_k$, for each factor, $f_k$, individual Fisher Scoring updates are applied to vech$(D_k)$. These updates are performed using the block of the Fisher Information matrix corresponding to vech$(D_k)$, given by (10), and take the following form for $k \in \{1,\ldots ,r\}$:

17 $$\begin{aligned}&\text {vech}(D_{k,s+1})\nonumber \\&\quad =\text {vech}(D_{k,s})+\alpha _s\big ({\mathcal {I}}^{h}_{\text {vech}(D_{k,s})}\big )^{-1}\frac{dl(\theta ^h_s)}{d\text {vech}(D_{k,s})}. \end{aligned}$$

In line with the naming convention used in Demidenko (2013), this method shall be referred to as “simplified” Fisher Scoring (SFS). This is due to the relative simplicity, both in terms of notational and computational complexity, of the updates (16) and (17) used in the SFS algorithm in comparison to those used in the FS algorithm of Sect. 2.1.1, given by (5)–(10). Pseudocode for the SFS algorithm is given by Algorithm 3.
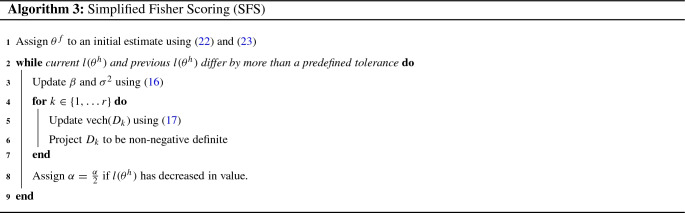


#### Full simplified Fisher Scoring

The Full Simplified Fisher Scoring algorithm (FSFS) combines the “Full” and “Simplified” approaches described in Sects. 2.1.2 and 2.1.3. In the FSFS algorithm, individual updates are applied to $\beta$ and $\sigma ^2$ using (16) and to $\{\text {vec}(D_k)\}_{k\in \{1,\ldots , r\}}$ using a Fisher Scoring update step, based on the matrix $F_{\text {vec}(D_{k})}$ given by (15). The update rule for $\{\text {vec}(D_k)\}_{k\in \{1,\ldots , r\}}$ takes the following form:

18 $$\begin{aligned} \text {vec}(D_{k,s+1})=\text {vec}(D_{k,s})+\alpha _s F^{-1}_{\text {vec}(D_{k,s})}\frac{\partial l(\theta ^f_s)}{\partial \text {vec}(D_{k,s})}. \end{aligned}$$

We note that the above can be seen to be equivalent to an update rule of the form (4), given by:

$$\begin{aligned} \text {vec}(D_{k,s+1})=\text {vec}(D_{k,s})+\alpha _s\big ({\mathcal {I}}^{f}_{\text {vec}(D_{k,s})}\big )^{+}\frac{\partial l(\theta ^f_s)}{\partial \text {vec}(D_{k,s})}. \end{aligned}$$

Justification of this claim can be found in Supplementary Material Section S13. Pseudocode for the FSFS algorithm is given by Algorithm 4.
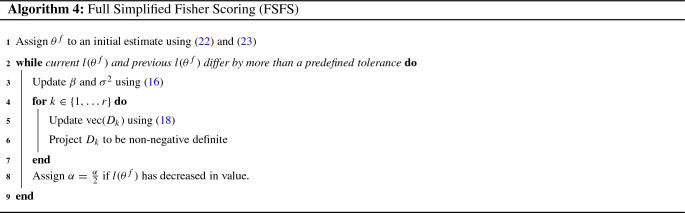


#### Cholesky simplified Fisher Scoring

The final variant of the Fisher Scoring algorithm we consider is based on the “simplified” approach described in Sect. 2.1.3 and uses the Cholesky parameterisation of $(\beta ,\sigma ^2,D)$, $\theta ^c$. This approach follows directly from the below application of the chain rule of differentiation for vector-valued functions,

$$\begin{aligned} \frac{dl(\theta ^c)}{d\text {vech}(\Lambda _k)}=\frac{\partial \text {vech}(D_k)}{\partial \text {vech}(\Lambda _k)}\frac{\partial l(\theta ^c)}{\partial \text {vech}(D_k)}. \end{aligned}$$

An expression for the derivative which appears second in the above product was given by (7). It therefore follows that in order to evaluate the score vector of $\text {vech}(\Lambda _k)$ (i.e. the derivative of *l* with respect to $\text {vech}(\Lambda _k)$), only an expression for the first term of the above product is required. This term can be evaluated to ${\mathcal {L}}_{q_k}(\Lambda _k' \otimes I_{q_k})(I_{q_k^2}+K_{q_k}){\mathcal {D}}_{q_k}$, proof of which is provided in Appendix 6.4.

Through similar arguments to those used to prove Corollaries 4–6 of Appendix 6.2, it can be shown that the Fisher Information matrix for $\theta ^c$ is given by:

$$\begin{aligned} {\mathcal {I}}^c_{\beta }={\mathcal {I}}^h_{\beta },\quad {\mathcal {I}}^c_{\beta ,\sigma ^2}={\mathcal {I}}^h_{\beta ,\sigma ^2},\quad {\mathcal {I}}^c_{\sigma ^2}={\mathcal {I}}^h_{\sigma ^2}. \end{aligned}$$

For $$k \in \{1,\ldots , r\}$$:

19 $$\begin{aligned} \begin{aligned}&{\mathcal {I}}^c_{\beta ,\text {vech}(\Lambda _k)}={\mathbf {0}}_{p,q_k(q_k+1)/2}, \\&{\mathcal {I}}^c_{\sigma ^2,\text {vech}(\Lambda _k)}= {\mathcal {I}}^h_{\sigma ^2,\text {vech}(D_k)}\bigg (\frac{\partial \text {vech}(D_k)}{\partial \text {vech}(\Lambda _k)}\bigg )'.\\ \end{aligned} \end{aligned}$$

For $$k_1,k_2\in \{1,\ldots ,r\}$$:

20 $$\begin{aligned} \begin{aligned}&{\mathcal {I}}^c_{\text {vech}(\Lambda _{k_1}),\text {vech}(\Lambda _{k_2})}\\&\quad =\bigg (\frac{\partial \text {vech}(D_{k_1})}{\partial \text {vech}(\Lambda _{k_1})}\bigg ){\mathcal {I}}^h_{\text {vech}(D_{k_1}),\text {vech}(D_{k_2})}\bigg (\frac{\partial \text {vech}(D_{k_2})}{\partial \text {vech}(\Lambda _{k_2})}\bigg )'. \end{aligned}\nonumber \\ \end{aligned}$$

From the above, it can be seen that a non-“simplified” Cholesky-based variant of the Fisher Scoring algorithm, akin to the FS and FFS algorithms described in Sects. 2.1.1 and 2.1.2, could be constructed. However, preliminary tests have indicated that the performance of such an approach, in terms of computation time, is significantly worse than the previously proposed algorithms. For this reason, we only consider the “simplified” version of the Cholesky Fisher Scoring algorithm, analogous to the “simplified” approaches described in Sects. 2.1.3 and 2.1.4, here. The Cholesky Simplified Fisher Scoring (CSFS) algorithm adopts (16) as the update rule for $\beta$ and $\sigma ^2$, and employs the following update rule for $\{\text {vech}(\Lambda _k)\}_{k \in \{1,\ldots ,r\}}$.

21 $$\begin{aligned}&\text {vech}(\Lambda _{k,s+1})=\nonumber \\&\text {vech}(\Lambda _{k,s})+\alpha _s\big ({\mathcal {I}}^{c}_{\text {vech}(\Lambda _{k,s})}\big )^{-1}\frac{dl(\theta ^c_s)}{d \text {vech}(\Lambda _{k,s})}. \end{aligned}$$

Pseudocode summarizing the CSFS approach is given by Algorithm 5.
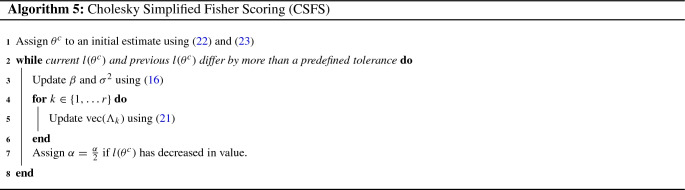


### Initial values

Choosing which initial values of $\beta$, $\sigma ^2$ and *D* will be used as starting points for optimization is an important consideration for the Fisher Scoring algorithm. Denoting these initial values as $\beta _0$, $\sigma ^2_0$ and $D_0$, respectively, this work follows the recommendations of Demidenko (2013) and evaluates $\beta _0$ and $\sigma ^2_0$ using the OLS estimates given by,

22 $$\begin{aligned} \beta _0 = (X'X)^{-1}X'Y, \quad \sigma ^2_0 = \frac{e_0'e_0}{n}. \end{aligned}$$

where $e_0$ is defined as $e_0=Y-X\beta _0$. For the initial estimate of $\{D_k\}_{k\in \{1,\dots ,r\}}$, an approach similar to that suggested in Demidenko (2013) is also adopted, which substitutes *V* for $I_n$ in the update rule for vec($D_k$), equation (18). The resulting initial estimate for $\{D_k\}_{k\in \{1,\dots ,r\}}$ is given by

23 $$\begin{aligned} \begin{aligned} \text {vec}(D_{k,0})&= \bigg (\sum _{j=1}^{l_k}Z_{(k,j)}'Z_{(k,j)} \otimes Z_{(k,j)}'Z_{(k,j)}\bigg )^{-1} \\&\quad \times \text {vec}\bigg (\sum _{j=1}^{l_k}Z_{(k,j)}'\bigg (\frac{e_0e_0'}{\sigma _0^2}-I_n\bigg )Z_{(k,j)}\bigg ) \end{aligned}. \end{aligned}$$

### Computational efficiency

This section provides discussion on the computational efficiency of evaluating the Fisher Information matrices and score vectors of Sects. 2.1.1–2.1.5. This discussion is, in large part, motivated by the mass-univariate setting (c.f. Sect. 1.1), in which not one, but rather hundreds of thousands of models must be estimated concurrently. As a result, this discussion prioritizes both time efficiency and memory consumption concerns and, further, operates under the assumption that sparse matrix methodology, such as that employed by the R package lme4, cannot be employed. This assumption is motivated by the current lack of support for computationally vectorized sparse matrix operations.

A primary concern, for both memory consumption and time efficiency, stems from the fact that many of the matrices used in the evaluation of the score vectors and Fisher Information matrices possess dimensions which scale with *n*, the number of observations. For example, *V* has dimensions $(n \times n)$ and is frequently inverted in the FSFS algorithm. In practice, it is not uncommon for studies to have *n* ranging into the thousands. As such, inverting *V* directly may not be computationally feasible. To address this issue, we define the “product forms” as;

$$\begin{aligned}&P=X'X,\quad Q=X'Y,\quad R=X'Z,\quad S=Y'Y,\quad T=Y'Z,\quad U=Z'Z. \end{aligned}$$

Working with the product forms is preferable to using the original matrices *X*, *Y* and *Z*, as the dimensions of the product forms do not scale with *n*, but instead scale with *p* and *q*. As an example, consider the expressions below, which appear frequently in (11) and (15) and have been reformulated in terms of the product forms:

$$\begin{aligned} \begin{aligned}&Z_{(k_1,i)}'V^{-1}Z_{(k_2,j)} = (U - UD(I_q+DU)^{-1}U)_{[(k_1,i),(k_2,j)]},\\&Z_{(k,j)}'V^{-1}e = ((I_q - UD)(I_q+DU)^{-1}(T'-R'\beta ))_{[(k,j),:]}. \end{aligned} \end{aligned}$$

For computational purposes, the right-hand side of the above expressions is much more convenient than the left-hand side. In order to evaluate the left-hand side in both cases, an $(n \times n)$ inversion of the matrix *V* must be performed. In contrast, the right-hand side is expressible solely in terms of the product forms and $(\beta , \sigma ^2, D)$, with the only inversion required being that of the $(q \times q)$ matrix $(I_q+DU)$. These examples can be generalized further. In fact, all of the previous expressions (5)–(23) can be rewritten in terms of only the product forms and $(\beta , \sigma ^2, D)$. This observation is important as it implies that an algorithm for mixed model parameter estimation may begin by taking the matrices *X*, *Y* and *Z* as inputs, but discard them entirely once the product forms have been constructed. As a result, both computation time and memory consumption no longer scale with *n*.

Another potential source of concern regarding computation speed arises from noting that the algorithms we have presented contain many summations of the following two forms:

24 $$\begin{aligned} \sum _{i=1}^{c_0} A_iB_i' \quad \text { and } \quad \sum _{i=1}^{c_1}\sum _{i=1}^{c_2} G_{i,j} \otimes H_{i,j}. \end{aligned}$$

where matrices $\{A_i\}$ and $\{B_i\}$ are of dimension $(m_1 \times m_2)$, and matrices $\{G_{i,j}\}$ and $\{H_{i,j}\}$ are of dimension $(n_1 \times n_2)$. We denote the matrices formed from vertical concatenation of the $\{A_i\}$ and $\{B_i\}$ matrices as *A* and *B*, respectively, and *G* and *H* the matrices formed from block-wise concatenation of $\{G_{i,j}\}$ and $\{H_{i,j}\}$, respectively. Instances of such summations can be found, for example, in Eqs. (7) and (10).

From a computational standpoint, summations of the forms shown in (24) are typically realized by “for” loop. This cumulative approach to computation can cause a potential issue for LMM computation since typically the number of summands corresponds to the number of levels, $l_k$, of some factor, $f_k$. In particular applications of the LMM, such as repeated measures and longitudinal studies, some factors may possess large quantities of levels. As this means “for” loops of this kind could hinder computation and result in slow performance, we provide alternative methods for calculating summations of the forms shown in (24).

For the former summation shown in (24), we utilize the “generalized vectorization”, or “vec$_m$” operator, defined by Turkington (2013) as the operator which performs the below mapping for a horizontally partitioned matrix *M*:

$$\begin{aligned} M = \begin{bmatrix} M_{1}&M_{2}&...&M_{c_0} \end{bmatrix} \rightarrow \text {vec}_{m}(M) = \begin{bmatrix} M'_{1}&M'_{2}&...&M'_{c_0} \end{bmatrix}' \end{aligned}$$

where the partitions $\{M_i\}$ are evenly sized and contain *m* columns. Using the definition of the generalized vectorization operator, the former summation in (24) can be reformulated as:

$$\begin{aligned} \sum _{i=1}^l A_iB_i'=\text {vec}_{m_2}(A')'\text {vec}_{m_2}(B'). \end{aligned}$$

The right-hand side of the expression above is of practical utility as the “vec$_m$” operator can be implemented efficiently. The “vec$_m$” operation can be performed, for instance, using matrix resizing operations such as the “reshape” operators commonly available in many programming languages such as MATLAB and Python. The computational costs associated with this approach are significantly lesser than those experienced when evaluating the summation directly using “for” loops.

For the latter summation in (24), we first define the notation ${\tilde{M}}$ to denote the transformation below, for the block-wise partitioned matrix *M*:

25 $$\begin{aligned} M = \begin{bmatrix} M_{1,1} &{} M_{1,2} &{} ... &{} M_{1,c_2} \\ M_{2,1} &{} M_{2,2} &{} ... &{} M_{2,c_2} \\ \vdots &{} \vdots &{} \ddots &{} \vdots \\ M_{c_1,1} &{} M_{c_1,2} &{} ... &{} M_{c_1,c_2} \end{bmatrix} \rightarrow {\tilde{M}} = \begin{bmatrix} \text {vec}(M_{1,1})' \\ \vdots \\ \text {vec}(M_{1,c_2})' \\ \text {vec}(M_{2,1})' \\ \vdots \\ \text {vec}(M_{c_1,c_2})'\end{bmatrix}.\nonumber \\ \end{aligned}$$

Using a modification of Lemma 3.1 (i) of Neudecker and Wansbeek (1983), we obtain the below identity:

26 $$\begin{aligned} \text {vec}\bigg (\sum _{i,j} G_{i,j}\otimes H_{i,j}\bigg ) = (I_{n_2} \otimes K_{n_1,n_2} \otimes I_{n_1})\text {vec}({\tilde{H}}'{\tilde{G}}),\nonumber \\ \end{aligned}$$

where $K_{n_1,n_2}$ is the $(n_1n_2\times n_1n_2)$ Commutation matrix (c.f. Sect. 1.2.2). The matrices ${\tilde{H}}$ and ${\tilde{G}}$ can be obtained from *H* and *G* using resizing operators with little computation time. The matrix $(I_{n_2} \otimes K_{n_1,n_2} \otimes I_{n_1})$ can be calculated via simple means and is a permutation matrix depending only on the dimensions of *H* and *G*. As a result, this matrix can be calculated once and stored as a vector. Therefore, the matrix multiplication in the above expression does not need to be performed directly, but instead can be evaluated by permuting the elements of vec$({\tilde{H}}'{\tilde{G}})$ according to the permutation vector representing $(I_{n_2} \otimes K_{n_1,n_2} \otimes I_{n_1})$. To summarize, evaluation of expressions of the latter form shown in (24) can be performed by using only reshape operations, a matrix multiplication, and a permutation. This method of evaluation provides notably faster computation time than the corresponding “for” loop evaluated over all values of *i* and *j*.

The above method, combined with the product form approach, was used to obtain the results of Sects. 3.1–4.2.

### Constrained covariance structure

In many applications involving the LMM, it is often desirable to use a constrained parameterisation for $D_k$. Examples include compound symmetry, first-order auto-regression and a Toeplitz structure. A more comprehensive list of commonly employed covariance structures in LMM analyses can be found, for example, in Wolfinger (1996). In this section, we describe how the Fisher Scoring algorithms of the previous sections can be adjusted to model dependence between covariance elements.

When a constraint is placed on the covariance matrix $D_k$, it is assumed that the elements of $D_k$ can be defined as continuous, differentiable functions of some smaller parameter vector, vecu$(D_k)$. Colloquially, vecu$(D_k)$ may be thought of as the vector of “unique” parameters required to specify the constrained parameterization of $D_k$. To perform constrained optimization, we adopt a similar approach to the previous sections and define the constrained representation of $\theta$ as $\theta ^{con}=[\beta ', \sigma ^2, \text {vecu}(D_1)',...\text {vecu}(D_k)']'$. Denoting the Jacobian matrix $\partial$vec$(D_k)/\partial$vecu$(D_k)$ as ${\mathcal {C}}_k$, the score vector and Fisher Information matrix of $\theta ^{con}$ can be constructed as follows; for $k \in \{1,..., r\}$,

27 $$\begin{aligned} \begin{aligned}&\frac{dl(\theta ^{con})}{d\text {vecu}(D_k)}={\mathcal {C}}_k\frac{\partial l(\theta ^{con})}{\partial \text {vec}(D_k)}, \\&{\mathcal {I}}^{con}_{\beta ,\text {vecu}({\tilde{D}}_k)}={\mathbf {0}}_{p,{\tilde{q}}_k}, \quad {\mathcal {I}}^{con}_{\sigma ^2,\text {vecu}({\tilde{D}}_k)}= {\mathcal {I}}^f_{\sigma ^2,\text {vec}(D_k)}{\mathcal {C}}_k', \end{aligned} \end{aligned}$$

and, for $$k_1,k_2\in \{1,\ldots ,r\}$$,

$$\begin{aligned} {\mathcal {I}}^{con}_{\text {vecu}({\tilde{D}}_{k_1}),\text {vecu}({\tilde{D}}_{k_2})}= {\mathcal {C}}_{k_1}{\mathcal {I}}^f_{\text {vec}(D_{k_1}),\text {vec}(D_{k_2})}{\mathcal {C}}_{k_2}', \end{aligned}$$

where ${\tilde{q}}_k$ is the length of vecu$(D_k)$ and ${\mathcal {I}}^f$ is the “full” Fisher Information matrix defined in Sect. 2.1.2. The above expressions can be derived trivially using the definition of the Fisher Information matrix and the chain rule. In the remainder of this work, the matrix ${\mathcal {C}}_k$ is referred to as a “constraint matrix” due to the fact it “imposes” constraints during optimization. A fuller discussion of constraint matrices, alongside examples, is provided in Supplementary Material Section S14.

We note here that this approach can be extended to situations in which all covariance parameters can be expressed in terms of one set of parameters, $\rho _D$, common to all $\{\text {vec}(D_k)\}_{k \in \{1,\ldots , r\}}$. In such situations, a constraint matrix, ${\mathcal {C}}$, may be defined as the Jacobian matrix $\partial [\text {vec}(D_1)',\ldots \text {vec}(D_1)']'/\partial \rho _D$ and Fisher Information matrices and score vectors may be derived in a similar manner to the above. Models requiring this type of commonality between factors are rare in the LMM literature, since the covariance matrices $\{D_k\}_{k \in \{1,\ldots r\}}$ are typically defined independently of one another. However, one such example is provided by the ACE model employed for twin studies, in which the elements of *v*(*D*) can all be expressed in terms of three variance components; $\rho _D=[\sigma ^2_a, \sigma ^2_c, \sigma ^2_e]'$. Further information on the ACE model is presented in Sect. 4.1.2.

### Degrees of freedom estimation

Several methods exist for drawing inference on the fixed effects parameter vector, $\beta$. Often, research questions can be expressed as null hypothesis tests of the below form:

$$\begin{aligned} H_0: L\beta = 0, \quad H_1 : L\beta \ne 0, \end{aligned}$$

where *L* is a fixed and known $(1 \times p)$-sized contrast vector specifying a hypothesis, or prior belief, about linear relationships between the elements of $\beta$, upon which an inference is to be made. In the setting of the LMM, a commonly employed statistic for testing hypotheses of this form is the approximate *T*-statistic, given by:

$$\begin{aligned} T = \frac{L{\hat{\beta }}}{\sqrt{{\hat{\sigma }}^2L(X'{\hat{V}}^{-1}X)^{-1}L'}}, \end{aligned}$$

where ${\hat{V}}=I_n+Z{\hat{D}}Z'$. As noted in Dempster et al. (1981), using an estimate of *D* in this setting results in an underestimation of the true variability in ${\hat{\beta }}$. For this reason, the relation $T \sim t_{v}$ is not exact and is treated only as an approximating distribution (i.e. an “approximate T statistic”) where the degrees of freedom, *v*, must be estimated empirically. A standard method for estimating the degrees of freedom, *v*, utilizes the Welch–Satterthwaite equation, originally described in Satterthwaite (1946) and Welch (1947), given by:

28 $$\begin{aligned} v({\hat{\eta }})=\frac{2(S^2({\hat{\eta }}))^2}{\text {Var}(S^2({\hat{\eta }}))}, \end{aligned}$$

where ${\hat{\eta }}$ represents an estimate of the variance parameters $\eta =(\sigma ^2,D_1,\ldots D_r)$ and $S^2({\hat{\eta }})={\hat{\sigma }}^2L(X'{\hat{V}}^{-1}X)^{-1}L'$. Typically, as the variance estimates obtained under ML estimation are biased downwards, ReML estimation is employed to obtain the estimate of $\eta$ employed in the above expression. If ML were used to estimate $\eta$ instead, the degrees of freedom, $v({\hat{\eta }})$, would be underestimated and, consequently, conservative *p*-values and a reduction in statistical power for resulting hypothesis tests would be observed.

To obtain an approximation for $v({\hat{\eta }})$, a second-order Taylor expansion is applied to the unknown variance on the denominator of (28):

29 $$\begin{aligned} \text {Var}(S^2({\hat{\eta }})) \approx \bigg (\frac{d S^2({\hat{\eta }})}{d {\hat{\eta }}}\bigg )'\text {Var}({\hat{\eta }})\bigg (\frac{d S^2({\hat{\eta }})}{d {\hat{\eta }}}\bigg ). \end{aligned}$$

This approach is well documented and has been adopted, most notably, by the R package lmerTest, first presented in Kuznetsova et al. (2017). To obtain the derivative of $S^2$ and asymptotic variance covariance matrix, lmerTest utilizes numerical estimation methods. As an alternative, we define the “half” representation of ${\hat{\eta }}$, ${\hat{\eta }}^h$, by ${\hat{\eta }}^h=[{\hat{\sigma }}^2, \text {vech}({\hat{D}}_1)',\ldots ,\text {vech}({\hat{D}}_r)']'$ and present the below exact closed-form expression for the derivative of $S^2$ in terms of ${\hat{\eta }}^h$;

$$\begin{aligned}&\frac{d S^2({\hat{\eta }}^h)}{d {\hat{\sigma }}^2}=L(X'{\hat{V}}^{-1}X)^{-1}L',\\&\frac{d S^2({\hat{\eta }}^h)}{d \text {vech}({\hat{D}}_k)} = {\hat{\sigma }}^{2}{\mathcal {D}}_{q_k}'\bigg (\sum _{j=1}^{l_k}{\hat{B}}_{(k,j)}\otimes {\hat{B}}_{(k,j)}\bigg ), \end{aligned}$$

where ${\hat{B}}_{(k,j)} = Z_{(k,j)}'{\hat{V}}^{-1}X(X'{\hat{V}}^{-1}X)^{-1}L'$. We obtain an expression for var$({\hat{\eta }}^h)$ by noting that the asymptotic variance of ${\hat{\eta }}^h$ is given by ${\mathcal {I}}({\hat{\eta }}^h)^{-1}$ where ${\mathcal {I}}({\hat{\eta }}^h)$ is a sub-matrix of ${\mathcal {I}}({\hat{\theta }}^h)$, given by equations (8)–(10).

In summary, we have provided all the closed-form expressions necessary to perform the Satterthwaite degrees of freedom estimation method for any LMM described by (1). For the remainder of this work, estimation of *v* using the above expressions is referred to as the “direct-SW” method. This name reflects the direct approach taken for the evaluation of the right-hand side of (29). We note that this approach may also be extended to models using the constrained covariance structures of Sect. 2.4 by employing the Fisher Information matrix given by Eq. (27) and transforming the derivative of $S^2(\eta )$ appropriately (details of which are provided by Theorem 7 of Appendix 6.5). We conclude this section by noting that this method can also be used in a similar manner for estimating the degrees of freedom of an approximate F-statistic based on the multi-factor LMM. Additional material describing how the Welch–Satterthwaite equation may be applied for approximate F-statistics is provided in Supplementary Material Section S15.

## Simulations

To assess the accuracy and efficiency of each of the proposed LMM parameter estimation methods described in Sects. 2.1.1–2.1.5 and the direct-SW degrees of freedom estimation method described in Sect. 2.5, extensive simulations were conducted. These simulations are described fully in Sect. 3.1 and the results are presented in Sect. 3.2. All reported results were obtained using an Intel(R) Xeon(R) Gold 6126 2.60GHz processor with 16GB RAM.

### Simulation methods

#### Parameter estimation

The algorithms of Sects. 2.1.1–2.1.5 have been implemented in the programming language Python. Under three different simulation settings, each with a different design structure, the algorithms are compared against one another, the ‘lmer’ function from the R package lme4, and the baseline truth used to generate the simulations. All methods are contrasted in terms of output, computation time and, for the methods presented in this paper, the number of iterations until convergence. Convergence of each method was assessed by verifying whether successive log-likelihood estimates differed by more than a predefined tolerance of $10^{-6}$. 1000 individual simulation instances were run for each simulation setting.

All model parameters were held fixed across all runs. In every simulation setting, test data were generated according to model (1). Each of the three simulation settings imposed a different structure on the random effects design and covariance matrices, *Z* and *D*. The first simulation setting employed a single factor design ($r = 1$) with two random effects (i.e. $q_1 = 2$) and 50 levels (i.e. $l_1 = 50$). The second simulation setting employed two crossed factors ($r=2$) where the number of random effects and numbers of levels for each factor were given by $q_1=3$, $q_2 =2$, $l_1=100$ and $l_2=50$, respectively. The third simulation setting used three crossed factors (i.e. $r= 3$) with the number of random effects and levels for each of the factors given by $q_1=4$, $q_2 =3$, $q_3 =2$, $l_1=100$, $l_2=50$ and $l_3=10$, respectively. In all simulations, the number of observations, *n*, was held fixed at 1000. In each simulated design, the first column of the fixed effects design matrix, *X*, and the first random effect in the random effects design matrix, *Z*, were treated as intercepts. Within each simulation instance, the remaining (nonzero) elements of the variables *X* and *Z*, as well as those of the error vector $\epsilon$, were generated at random according to the standard univariate normal distribution. The random effects vector *b* was simulated according to a normal distribution with covariance *D*, where *D* was predefined, exhibited no particular constrained structure and contained a mixture of both zero and nonzero off-diagonal elements. The assignment of observations to levels for each factor was performed at random with the probability of an observation belonging to any specific level held constant and uniform across all levels.

Fisher Scoring methods for the single-factor design have been well studied (c.f. Demidenko 2013), and the inclusion of the first simulation setting is only for comparison purposes. The second and third simulation settings represent more complex crossed factor designs for which the proposed Fisher Scoring-based parameter estimation methods did not previously exist. To assess the performance of the proposed algorithms, the mean absolute error (*MAE*) and mean relative difference (*MRD*) were used as performance metrics. The methods considered for parameter estimation were those described in Sects. 2.1.1–2.1.5, and the baseline truth used for comparison was either the baseline truth used to generate the simulated data or the lmer computed estimates.

All methods were contrasted in terms of the *MAE* and *MRD* of both $\beta$ and the variance product $\sigma ^2D$. The variance product $\sigma ^2D$ was chosen for comparison instead of the individual components $\sigma ^2$ and *D* as the variance product $\sigma ^2D$ is typically of intrinsic interest during the inference stage of conventional statistical analyses and is often employed for further computation. In all simulations, both ML and ReML estimation variants of the methods were assessed. The computation time for each method was also recorded and contrasted against that of lmer. To ensure a fair comparison of computation time, the recorded computation times for lmer were based only on the performance of the “optimizeLmer” function, which is the module employed for parameter estimation by lmer. The specific values of $\beta , \sigma ^2,$ and *D* employed for each simulation setting can be found in Section S1 of the Supplementary Material. Formal definitions of MAE and MRD measures used for comparison are given in Section S2 of the Supplementary Material.

#### Degrees of freedom estimation

The accuracy and validity of the direct-SW degrees of freedom estimation method proposed in Sect. 2.5 were assessed through further simulation. To achieve this, as LMM degrees of freedom are assumed to be specific to the experiment design, a single design was chosen at random from each of the three simulation settings described in Sect. 3.1.1. With the fixed effects and random effects matrices, *X* and *Z*, now held constant across simulations, 1000 simulations were run for each simulation setting. The random effects vector, *b*, and random error vector, $\epsilon$, were allowed to vary across simulations according to normal distributions with the appropriate variances. In each simulation, degrees of freedom were estimated via the direct-SW method for a predefined contrast vector, corresponding to a fixed effect that was truly zero.

The direct-SW estimated degrees of freedom were compared to a baseline truth and the degrees of freedom estimates produced by the R package lmerTest. Baseline truth was established in each simulation setting using 1,000,000 simulations to empirically estimate $\text {Var}(S^2({\hat{\eta }}))$, giving a single value for the denominator of $v({\hat{\eta }})$ from Equation (28). Following this, in each of the 1000 simulation instances described above, the numerator of (28) was recorded, giving 1000 estimates of $v({\hat{\eta }})$. The final estimate was obtained as an average of these 1000 values. All lmerTest degrees of freedom estimates were obtained using the same simulated data as was used by the direct-SW method and were computed using the “contest1D” function from the lmerTest package. While all baseline truth measures were computed using parameter estimates obtained using the FSFS algorithm of Sect. 2.1.4, we believe this has not induced bias into the simulations as, as discussed in Sect. 3.2.1, all simulated FSFS-derived parameter estimates considered in this work agreed with those provided by lmer within a tolerance level on the scale of machine error.

### Simulation results

#### Parameter estimation results

The results of the parameter estimation simulations of 3.1.1 were identical. In all three settings, all parameter estimates and maximised likelihood criteria produced by the FS, FFS, SFS and FSFS methods were identical to those produced by lmer up to a machine error level of tolerance. In terms of the maximisation of likelihood criteria, there was no evidence to suggest that lmer outperformed any of the FS, FFS, SFS or FSFS methods, or vice versa. The CSFS method provided worse performance, however, with maximised likelihood criteria which were lower than those reported by lmer in approximately $2.5\%$ of the simulations run for simulation setting 3. The reported maximised likelihood criteria for these simulations were all indicative of convergence failure. For all methods considered during simulation, the observed performance was unaffected by the choice of likelihood estimation criteria (i.e. ML or ReML) employed.

**Table 1:** The average time in seconds and the number of iterations reported for maximum likelihood estimation performed using the FS, FFS, SFS, FSFS and CSFS methods. For each simulation setting, results displayed are taken from averaging across 1000 individual simulations. Also given are the average times taken by lmer to perform maximum likelihood parameter estimation on the same simulated data. Standard deviations are given in brackets below each entry in the table

Method	FS	FFS	SFS	FSFS	CSFS	lmer
*Simulation* 1						
*t* (Time/s)	0.041	0.037	0.026	0.055	0.057	0.059
	(0.012)	(0.011)	(0.007)	(0.058)	(0.017)	(0.021)
$n_{it}$ (No. of iterations)	5.243	5.243	6.048	6.048	10.461	–
	(0.613)	(0.613)	(0.256)	(0.256)	(1.851)	–
*Simulation* 2						
*t* (Time/s)	0.129	0.112	0.105	0.125	0.220	0.648
	(0.047)	(0.042)	(0.036)	(0.061)	(0.070)	(0.050)
$n_{it}$ (No. of iterations)	6.694	6.694	8.323	8.323	14.281	–
	(0.964)	(0.964)	(0.534)	(0.534)	(1.381)	–
*Simulation* 3						
*t* (Time/s)	1.081	0.889	1.872	1.941	3.970	5.979
	(0.434)	(0.429)	(0.915)	(0.869)	(1.094)	(0.299)
$n_{it}$ (No. of iterations)	8.133	8.133	16.054	16.054	33.437	–
	(0.743)	(0.743)	(0.835)	(0.835)	(24.265)	–

The *MRD* and *MAE* values provided evidence for strong agreement between the parameter estimates produced by lmer and the FS, FFS, SFS and FSFS methods. The largest *MRD* values observed for these methods, taken relative to lmer, across all simulations and likelihood criteria, were $1.03 \times 10^{-3}$ and $2.12 \times 10^{-3}$ for $\beta$ and $\sigma ^2D$, respectively. The largest observed *MAE* values for these methods, taken relative to lmer, across all simulations, were given by $1.02 \times 10^{-5}$ and $4.30 \times 10^{-4}$ for $\beta$ and $\sigma ^2D$, respectively. Due to the extremely small magnitudes of these values, *MAE* and *MRD* values are not reported in detail here. For further detail, see Supplementary Material Sections S3–S6.

To compare the proposed methods in terms of computational performance, Tables 1 and 2 present the average time and number of iterations performed for each of the 5 methods, using ML and ReML likelihood criteria, respectively. Corresponding computation times are also provided for lmer. For both ML and ReML likelihood criteria, all Fisher Scoring methods demonstrated considerable efficiency in terms of computation speed. While the improvement in speed is minor for simulation setting 1, for multi-factor simulation settings 2 and 3, the performance gains can be seen to be considerable. Overall, the only method that consistently demonstrated notably worse performance than the others was the CSFS algorithm. In addition to requiring a longer computation time, the CSFS algorithm employed many more iterations.

Within a setting where *q* (i.e. the number of columns in the random effects design matrix) is small, the results of these simulations demonstrate strong computational efficiency for the FS, FFS, SFS and FSFS methods. However, we emphasise that no claim is made to suggest that the observed results will generalise to larger values of *q* or all possible designs. For large sparse LMMs that include a large number of random effects, without further adaptation of the methods presented in this work to employ sparse matrix methodology, it is expected that lmer will provide superior performance. We again emphasise here that our purpose in undertaking this work is not to compete with the existing LMM parameter estimation tools. Instead, the focus of this work is to develop methodology, which provides performance comparable to the existing tools, for use in situations where the existing tools may not be applicable due to practical implementation considerations.

**Table 2:** The average time in seconds and the number of iterations reported for restricted maximum likelihood estimation performed using the FS, FFS, SFS, FSFS and CSFS methods. For each simulation setting, results displayed are taken from averaging across 1000 individual simulations. Also given are the average times taken by lmer to perform restricted maximum likelihood parameter estimation on the same simulated data. Standard deviations are given in brackets below each entry in the table

Method	FS	FFS	SFS	FSFS	CSFS	lmer
*Simulation* 1						
*t* (Time/s)	0.057	0.052	0.043	0.064	0.088	0.071
	(0.014)	(0.011)	(0.008)	(0.042)	(0.021)	(0.021)
$n_{it}$ (No. of iterations)	5.261	5.261	6.053	6.053	10.404	–
	(0.591)	(0.591)	(0.257)	(0.257)	(1.829)	–
*Simulation* 2						
*t* (Time/s)	0.175	0.154	0.157	0.169	0.306	0.810
	(0.043)	(0.039)	(0.035)	(0.041)	(0.067)	(0.065)
$n_{it}$ (No. of iterations)	6.758	6.758	8.413	8.413	14.283	–
	(0.984)	(0.984)	(0.561)	(0.561)	(1.404)	–
*Simulation* 3						
*t* (Time/s)	1.615	1.378	2.968	3.002	6.604	7.457
	(0.404)	(0.344)	(0.752)	(0.814)	(5.455)	(0.442)
$n_{it}$ (No. of iterations)	8.084	8.084	16.018	16.018	34.611	–
	(0.730)	(0.730)	(0.798)	(0.798)	(25.526)	–

It must be stressed that reported computation times do not solely reflect theoretical differences between the proposed methods and those employed by lmer. Practical considerations related to implementation, such as which programming language and software libraries are used, also influence computational performance. Again, we emphasize that the aim of these simulations is not to perform a like-for-like comparison between software packages but instead, to demonstrate the viability and efficacy of our proposed methods as assessed in relation to the tools currently available.

Two potential causes for the poor performance of the CSFS method have been identified. The first cause is presented in Demidenko (2013), in which it is argued that the Cholesky parameterised algorithm will provide slower convergence than that of the unconstrained alternative methods due to structural differences between the likelihood surfaces over which parameter estimation is performed. This reasoning offers a likely explanation for the higher number of iterations and computation time observed for the CSFS method across all simulations. However, this does not explain the small number of simulations in simulation setting 3 in which evidence of convergence failure was observed.

The second possible cause also provides an explanation for the observed convergence failure. Pinheiro and Bates (1996) note that Cholesky parameterisations are not unique and the number of possible choices for the Cholesky factorisation of a positive definite matrix of dimension $(q \times q)$ increases with the dimension *q*. This means that, for each covariance matrix $D_k$, there are multiple Cholesky factors, $\Lambda _k$, which satisfy $\Lambda _k\Lambda _k'=D_k$. The larger $D_k$ is in dimension, the greater the number of $\Lambda _k$ that correspond to $D_k$ there are. Pinheiro and Bates (1996) argue that when optimal solutions are numerous and close together in the parameter space, numerical problems can arise during optimisation. We note that in comparison to the other simulation settings considered here, the design for simulation setting 3 contains the largest number of factors and random effects. Consequently, the covariance matrices, $D_k$, are large for this simulation setting and numerous Cholesky factors which correspond to the same optimal solution for the covariance matrix, *D*, exist. For this reason, simulation setting 3 is the most susceptible to numerical problems of the kind described by Pinheiro and Bates (1996). We suggest that this is a likely accounting for the 2.5% of simulations in which convergence failure was observed. In summary, the simulation results offer strong evidence for the correctness and efficiency of the Fisher Scoring methods proposed, with the exception of the CSFS method, which experiences slower performance and convergence failure in rare cases.

**Table 3:** The mean, standard deviation and mean squared error for 1, 000 degrees of freedom estimates in each simulation setting. Results are displayed for both the lmerTest and direct-SW methods, alongside “true” mean values, which were established using the moment-matching based approach outlined in Sect. 3.1.2 and computed using 1, 000, 000 simulation instances

Method	Truth	Direct-SW	lmerTest
*Simulation* 1			
Mean	910.93	910.68	906.62
Standard deviation	0.0	2.36	2.39
Mean squared error	0.0	5.64	24.23
*Simulation* 2			
Mean	844.61	842.17	837.79
Standard deviation	0.0	7.16	7.26
Mean squared error	0.0	57.20	99.21
*Simulation* 3			
Mean	707.01	700.96	695.08
Standard deviation	0.0	17.19	17.67
Mean squared error	0.0	332.01	454.53

#### Degrees of freedom estimation results

Across all degrees of freedom simulations, results indicated that the degrees of freedom estimates produced by the direct-SW method possessed both lower bias and lower variance than those produced by lmerTest. The degrees of freedom estimates, for each of the three simulation settings, are summarized in Table 3.

It can be seen from Table 3 that throughout all simulation settings both direct-SW and lmerTest appear to underestimate the true value of the degrees of freedom. However, the bias observed for the lmerTest estimates is notably more severe that of the direct-SW method, suggesting that the estimates produced by direct-SW have a higher accuracy than those produced by lmerTest. The observed difference in the standard deviation of the degrees of freedom estimates between the lmerTest and direct-SW methods is less pronounced. However, in all simulation settings, lower standard deviations are reported for direct-SW, suggesting that the estimates produced by direct-SW have a higher precision than those produced by lmerTest.

For both lmerTest and direct-SW, the observed bias and variance increase with simulation complexity. The simulations provided here indicate that the severity of disagreement between direct-SW and lmerTest increases as the complexity of the random effects design increases. This observation matches the expectation that the accuracy of the numerical gradient estimation employed by lmerTest will worsen as the complexity of the parameter space increases. Reported mean squared errors are also provided, indicating further that the direct-SW method outperformed lmerTest in terms of accuracy and precision in all simulation settings.

## Real data examples

To illustrate the usage of the methodology presented in Sects. 2.1–2.5 in practical situations, we provide two real data examples. These examples are described fully in Sect. 4.1, and the results are presented in Sect. 4.2. Again, all reported results were obtained using an Intel(R) Xeon(R) Gold 6126 2.60 GHz processor with 16GB RAM. For each reported computation time, averages were taken across 50 repeated runs of the given analysis.

### Real data methods

#### The SAT score example

The first example presented here is based on data from the longitudinal evaluation of school change and performance (LESCP) dataset (Turnbull et al. 1999). This dataset has notably been previously analysed by Hong and Raudenbush (2008) and was chosen for inclusion in this work because it previously formed the basis for between-software comparisons of LMM software packages by West et al. (2014). The LESCP study was conducted in 67 American schools in which SAT (student aptitude test) math scores were recorded for randomly selected samples of students. As in West et al. (2014), one of the 67 schools from this dataset was chosen as the focus for this analysis. For each individual SAT score, unique identifiers (ID’s) for the student who took the test and for the teacher who prepared the student for the test were recorded. As many students were taught by multiple teachers and all teachers taught multiple students, the grouping of SAT scores by student ID and the grouping of SAT scores by teacher ID constitute two crossed factors. In total, $n=234$ SAT scores were considered for analysis. The SAT scores were taken from 122 students taught by a total of 12 teachers, with each student sitting between 1 and 3 tests.

The research question in this example concerns how well a student’s grade (i.e. year of schooling) predicted their mathematics SAT score (i.e. did students improve in mathematics over the course of their education?). For this question, between-student variance and between-teacher variance must be taken into consideration as different students possess different aptitudes for mathematics exams and different teachers possess different aptitudes for teaching mathematics. In practice, this is achieved by employing an LMM which includes random intercept terms for both the grouping of SAT scores according to student ID and the grouping of SAT scores according to teacher ID. For the *k*th mathematics SAT test taken by the *i*th student, under the supervision of the *j*th teacher, such a model could be stated as follows:

$$\begin{aligned} \text {MATH}_{i,j,k} = \beta _0 + \beta _1 \times \text {YEAR}_{i,j,k} + s_i + t_j + \epsilon _{i,j,k}, \end{aligned}$$

where MATH$_{i,j,k}$ is the SAT score achieved and YEAR$_{i,j,k}$ is the grade of the student at the time the test was taken. In the above model, $\beta _0$ and $\beta _1$ are unknown parameters and $s_i$, $t_j$ and $\epsilon _{i,j,k}$ are independent mean-zero random variables which differ only in terms of their covariance. $s_i$ is the random intercept which models between-student variance, $t_j$ is the random intercept which models between-teacher variance, and $\epsilon _{i,j,k}$ is the random error term. The random variables $s_i$, $t_j$ and $\epsilon _{i,j,k}$ are assumed to be mutually independent and follow the below distributions:

$$\begin{aligned} \begin{aligned}&s_i \sim N(0, \sigma ^2_s), \quad t_j \sim N(0, \sigma ^2_t), \quad \epsilon _{i,j,k} \sim N(0,\sigma ^2), \end{aligned} \end{aligned}$$

where the parameters $\sigma ^2_s$, $\sigma ^2_t$ and $\sigma ^2$ are the unknown student, teacher and residual variance parameters, respectively.

The random effects in the SAT score model can be described using the notation presented in previous sections as follows; $r=2$ (i.e. observations are grouped by two factors, student ID and teacher ID), $q_1=q_2=1$ (i.e. one random effect is included for each factor, the random intercepts $s_i$ and $t_j$, respectively), and $l_1=122,l_2=12$ (i.e. there are 122 students and 12 teachers). When the model is expressed in the form described by (1), the random effects design matrix *Z* is a $0-1$ matrix. In this setting, the positioning of the nonzero elements in *Z* indicates the student and teacher associated with each test score. The random effects covariance matrices for the two factors, student ID and teacher ID, are given by $D_0=[\sigma ^2_s]$ and $D_1=[\sigma ^2_t]$, respectively.

For the SAT score model, the estimated parameters obtained using each of the methods detailed in Sects. 2.1.1–2.1.5 are reported. For comparison, the parameter estimates obtained by lmer are also given. As the estimates for the fixed effects parameters, $\beta _0$ and $\beta _1$, are of primary interest, ML was employed to obtain all reported parameter estimates. For this example, methods are contrasted in terms of output, computation time and the number of iterations performed.

#### The twin study example

The second example presented in this work aims to demonstrate the flexibility of the constrained covariance approaches described in Sect. 2.4. This example is based on the ACE model, an LMM commonly employed to analyse the results of twin studies by accounting for the complex covariance structure exhibited between related individuals. The ACE model achieves this by separating between-subject response variation into three categories: variance due to additive genetic effects ($\sigma ^2_a$), variance due to common environmental factors ($\sigma ^2_c$), and residual error ($\sigma ^2_e$). For this example, we utilize data from the Wu-Minn Human Connectome Project (HCP) (Van Essen et al. 2013). The HCP dataset contains brain imaging data collected from 1, 200 healthy young adults, aged between 22 and 35, including data from 300 twin pairs and their siblings. We do not make use the imaging data in the HCP dataset but, instead, focus on the baseline variables for cognition and alertness.

The primary research question considered in this example focuses on how well a subject’s quality of sleep predicts their English reading ability. The variables of primary interest used to address this question are subject scores in the Pittsburgh Sleep Quality Index (PSQI) questionnaire and an English language recognition test (ENG). Other variables included the subjects’ age in years (AGE) and sex (SEX), as well as an age–sex interaction effect. A secondary research question considered asks “How much of the between-subject variance observed in English reading ability test scores can be explained by additive genetic and common environmental factors?”. To address this question, the covariance parameters $\sigma ^2_a$, $\sigma ^2_c$ and $\sigma ^2_e$ must be estimated.

To model the covariance components of the ACE model, a simplifying assumption that all family units share common environmental factors was made. Following the work of Winkler et al. (2015), family units were first categorized by their internal structure into what shall be referred to as “family structure types” (i.e. unique combinations of full-siblings, half-siblings and identical twin pairs which form a family unit present in the HCP dataset). In the HCP dataset, 19 such family structure types were identified. In the following text, each family structure type shall be treated as a factor in the model. For the *i*th observation to belong to the *j*th level of the *k*th factor in the model may be interpreted as the *i*th subject belonging to the *j*th family exhibiting family structure of type *k*. The model employed for this example is given by:

$$\begin{aligned} \text {ENG}_{k,j,i}= & {} \beta _0 + \beta _1 \times \text {AGE}_{i} + \beta _2 \times \text {SEX}_{i} + \ldots \beta _3\\&\times \text {AGE}_{i} \times \text {SEX}_{i} + \beta _4 \times \text {PSQI}_{i} + \gamma _{k,j,i} + \epsilon _{k,j,i}, \end{aligned}$$

where both $\gamma _{k,j,i}$ and $\epsilon _{k,j,i}$ are mean-zero random variables. The random error, $\epsilon _{k,j,i}$, is distributed $N(0,\sigma ^2_e)$, and the random term $\gamma _{k,j,i}$ models the within-“family unit” covariance. Further detail on the specification of $\gamma _{k,j,i}$ can be found in Appendix (6.6.1).

**Table 4:** Performance metrics, parameter estimates and approximate *T*-test results for the twin study example. Standard errors for the fixed effects parameter estimates are given in brackets alongside the corresponding estimates. For each model parameter, a *T*-statistic, direct-SW degrees of freedom estimate, and *p*-value are provided, corresponding to the approximate *t*-test for a nonzero effect. *p*-values that are significant at the 5% level are indicated using a $^*$ symbol

Estimation method		OLS	Powell	FS
*Performance*				
*l* (Log-likelihood)		$-2133.49$	$-2005.83$	$-2005.81$
*t* (Time in seconds)		$<.001$	93.32	2.31
*Fixed effects parameters (Standard Errors)*				
$\beta _0$ (Intercept)		121.46 (3.61)	118.43 (3.40)	118.34 (3.42)
$\beta _1$ (Age)		$-0.11$ (0.12)	$-0.04$ (0.11)	$-0.04$ (0.11)
$\beta _2$ (Sex)		$-10.74$ (5.07)	$-7.22$ (4.64)	$-7.61$ (4.66)
$\beta _3$ (Age/sex)		0.45 (0.18)	0.33 (0.16)	0.34 (0.16)
$\beta _4$ (PSQI score)		$-0.49$ (0.12)	$-0.31$ (0.10)	$-0.30$ (0.10)
*Covariance parameters*				
$\sigma ^2_a$ (Additive genetic)		0.00	48.20	50.67
$\sigma ^2_c$ (Common environment)		0.00	27.53	26.89
$\sigma ^2_e$ (Residual error)		110.04	33.57	32.71
*Tests for Fixed Effects*				
	*T* statistic	33.67	34.83	34.65
Intercept	*degrees of freedom*	1105.00	920.59	921.57
	*p* value	$<.001^*$	$<.001^*$	$<.001^*$
	*T* statistic	$-0.94$	$-0.350$	$-0.320$
Age	*degrees of freedom*	1105.00	907.44	908.42
	*p* value	.350	.726	.749
	*T* statistic	$-2.12$	$-1.56$	$-1.63$
Sex	*degrees of freedom*	1105.00	833.01	832.96
	*p* value	$.034^*$	.120	.103
	*t-statistic*	2.58	2.03	2.10
Age/sex	*degrees of freedom*	1105.00	830.53	830.61
	*p* value	$.010^*$	$.043^*$	$.036^*$
	*T* statistic	$-4.25$	$-3.19$	$-3.17$
PSQI score	*degrees of freedom*	1105.00	933.67	928.87
	*p* value	$<.001^*$	$.001^*$	$.001^*$

In the notation of the previous sections, the number of factors in the ACE model, *r*, is equal to the number of family structure types present in the model (i.e. 19). For each family structure type present in the model, family structure type *k*, $l_k$ is the number of families who exhibit such structure and $q_k$ is the number of subjects present in any given family unit with such structure. As there is a unique random effect (i.e. a unique random variable, $\gamma _{k,j,i}$) associated with each individual subject, none of which are scaled by any coefficients, the random effects design matrix, *Z*, is the $(n \times n)$ identity matrix. To describe $\{D_k\}_{k \in \{1,\ldots ,r\}}$ requires the known matrices ${\mathbf {K}}^a_k$ and ${\mathbf {K}}^c_k$, which specify the kinship (expected genetic material) and environmental effects shared between individuals, respectively (See Supplementary Material Section S16.1 for more details). Given ${\mathbf {K}}^a_k$ and ${\mathbf {K}}^c_k$, the covariance components $\sigma ^2$ and $\{D_k\}_{k \in \{1,\ldots ,r\}}$ are given as $\sigma ^2=\sigma ^2_e$ and $D_k = \sigma ^{-2}_e(\sigma ^2_a{\mathbf {K}}^a_k + \sigma ^2_c{\mathbf {K}}^c_k)$, respectively.

As the covariance components $\sigma _a$ and $\sigma _c$ are of practical interest in this example, optimization is performed according to the ReML criterion using the adjustments described in Appendix 6.3 and covariance structure is constrained via the methods outlined in Sect. 2.4. Further detail on the constrained approach for the ACE model can be found in Appendix 6.6.2. Discussion of computational efficiency for the ACE model is also provided in Supplementary Material Section S16.2.

Section 4.2.2 reports the maximized restricted log-likelihood values and parameter estimates obtained using the Fisher Scoring method. Also given are approximate *T*-statistics for each fixed effects parameter, alongside corresponding degrees of freedom estimates and *p*-values obtained via the methods outlined in Sect. 2.5. To verify correctness, the restricted log-likelihood of the ACE model was also maximized numerically using the implementation of Powell’s bi-directional search-based optimization method (Powell 1964) provided by the SciPy Python package. The maximized restricted log-likelihood values and parameter estimates produced were then contrasted against those provided by the Fisher Scoring method. The OLS (ordinary least squares) estimates, which would be obtained had the additive genetic and common environmental variance components not been accounted for in the LMM analysis, are also provided for comparison.

### Real data results

#### The SAT score results

For the SAT score example described in Sect. 4.1.1, the log-likelihood, fixed effect parameters and variance components estimates produced by the Fisher Scoring algorithms were identical to those produced by lmer. Further, the reported computation times for this example suggested little difference between all methods considered in terms of computational efficiency. Of the Fisher Scoring methods considered, the FS and FFS methods took the fewest iterations to converge, while the SFS and FSFS methods took the most iterations to converge. The results presented here exhibit strong agreement with those reported in West et al. (2014), in which the same model was used as the basis for a between-software comparison of LMM software packages. For completeness, the full table of results can be found in Supplementary Material Section S9.

#### The twin study results

The results for the twin study example described in Sect. 4.1.2 are presented in Table 4. It can be seen from Table 4 that the Powell optimizer and Fisher Scoring method attained extremely similar optimized likelihood values, with Fisher Scoring converging notably faster. This result offers further evidence for the correctness of the parameter estimates produced by the Fisher Scoring method as it is unlikely that both Powell estimation and Fisher Scoring would converge to the same solution if the solution were suboptimal. The parameter estimates produced by Fisher Scoring and Powell optimization can be seen to be smaller in magnitude than those estimated by OLS, highlighting how the inclusion of additional variance terms in the model can have a meaningful impact on the conclusion of the analysis.

Also provided in Table 4 are approximate *t*-tests based on the Fisher Scoring and Powell optimizer parameter estimates, with corresponding standard *t*-tests given based on the OLS estimates. At the 5% significance level, the approximate *t*-tests conclude that the fixed effects parameters corresponding to the “Intercept”, “Age and Sex Interaction” and the “PSQI Score” are nonzero in value. While it may be expected that age should affect reading ability, no significant effect was observed for the “Age” covariate. This lack of observed effect may be explained by the narrow age range of subjects present in the HCP dataset, with subjects ranging from 22 to 35 years in age, and by the fact that individual observations were recorded in units of years. In general, the OLS-based *t*-tests produced similar conclusions to those produced by the FS-based approximate *t*-tests. A notable exception, however, is given by the *t*-tests for the fixed effect associated with the “Sex” covariate. While the standard OLS *t*-test reported at the 5% significance level that the “Sex” fixed effect was nonzero in value, the FS-based approximate *t*-test concluded that there was not evidence to support this claim. This result further highlights the importance of modelling all relevant variance terms.

## Discussion

In this work, we have presented derivations for and demonstrated potential applications of, score vector and Fisher Information matrix expressions for the LMMs containing multiple random factors. While many of the examples presented in this paper were benchmarked against existing software, it is not the authors’ intention to suggest that the proposed methods are superior to existing software packages. Instead, this work aims to complement existing LMM parameter estimation research. This aim is realized through careful exposition of the score vectors and Fisher Information matrices and detailed description of methodology and algorithms.

Modern approaches to LMM parameter estimation typically depend upon conceptually complex mathematical operations which require support from a range of software packages and infrastructure. This work has been, in large part, motivated by current deficiencies in vectorized support for such operations. Vectorized computation is a crucial requirement for medical imaging applications, in which hundreds of thousands of mixed models must be estimated concurrently. The methods proposed in this work take advantage over the existing tools in such situations, where many LMM’s must be run concurrently, as the theoretically simplistic mathematical operations they employ are more naturally amenable to vectorized computation. It is with such applications in mind that this work has been undertaken. We intend to pursue the application of mass-univariate analysis itself in future work.

Although the methods presented in this work are well suited for the desired application, we stress that there are many situations where current software packages will likely provide superior performance. One such situation can be found by observing that the Fisher Scoring method requires the storage and inversion of a matrix of dimensions $(q \times q)$. This is problematic, both in terms of memory and computation accuracy, for designs which involve very large numbers of random effects, grouping factors or factor levels. While such applications have not been considered extensively in this work, we note that many of the expressions provided in this document may benefit from combination with sparse matrix methodology to overcome this issue. We suggest that the potential for improved computation time via the combination of the Fisher Scoring approaches described in this paper with sparse matrix methodology may also be the subject of future research.

An active area of LMM research that has not been discussed extensively in this work is hypothesis testing for random effects covariance parameters. In general, development of hypothesis testing procedures for random effects is a more complex task than for that of fixed effects. This additional complexity stems from the fact that the random effects parameters can lie on the boundary of the parameter space and must be modelled using mixture distributions. Many methods are available which provide approximate testing procedures for random effects covariance parameters (c.f. Scheipl et al. 2008). However, it is not immediately apparent to us whether the derivations we have provided may be used in conjunction with such methods to improve the testing procedures for random effects. We suggest this idea may form a potential basis for future investigation.

## Appendix

### Score vectors

In this appendix, we provide full derivations for the derivatives (7) and (11). For derivatives (5) and (6), we note that the derivations for the multi-factor LMM are identical to those given for the single-factor setting in Demidenko (2013). As a result, we refer the reader to this source for proofs. To obtain the derivatives (7) and (11), two lemmas, Lemma 1 and Lemma 2, are required. Proofs for Lemma 1 and Lemma 2, alongside discussion of the definition of derivative employed throughout this paper, are provided by Supplementary Material Section S12.

#### Lemma 1

Let *g* be a column vector, *A* be a square matrix and $\{B_s\}$ be a set of arbitrary matrices of equal size. Let *K* be an unstructured matrix which none of *g*, *A* or any of the $\{B_s\}$ depend on. Further, assume $A,\{B_s\}, g$ and *K* can be multiplied as required. The below matrix derivative expression now holds;

30 $$\begin{aligned} \begin{aligned}&\frac{\partial }{\partial K} \bigg [ g'(A+\sum _t B_tKB_t')^{-1}g \bigg ]\\&\quad =-\sum _s B_s'\big (A'+\sum _t B_tKB_t'\big )^{-1}gg'\big (A'+\sum _t B_tKB_t'\big )^{-1}B_s. \end{aligned}\nonumber \\ \end{aligned}$$

#### Lemma 2

Let $A, \{B_s\}$ and *K* be defined as in Lemma 1. Then, the following is true:

31 $$\begin{aligned} \frac{\partial }{\partial K} \log |A+\sum _t B_tKB_t'| = \sum _s B_s'(A+\sum _t B_tK'B_t')^{-1}B_s.\nonumber \\ \end{aligned}$$

We first derive the partial derivative matrix $\frac{\partial l}{\partial D_k}$ (i.e. the matrix derivative with respect to $D_k$ which does not account for the equal elements of $D_k$ induced by symmetry).

#### Theorem 1

The partial derivative matrix $\frac{\partial l}{\partial D_k}$ is given by the following.

32 $$\begin{aligned} \frac{\partial l(\theta )}{\partial D_k} = \frac{1}{2}\sum _{j=1}^{l_k}Z_{(k,j)}'V^{-1}\bigg (\frac{ee'}{\sigma ^2}-V\bigg )V^{-1}Z_{(k,j)}. \end{aligned}$$

#### Proof

To derive (32), we use the expression for the log-likelihood of the LMM, given by (2). As the first term inside the brackets of (2) does not depend on $D_k$, we need only consider the second and third term for differentiation. We now note that, by the construction of the random effects design matrix, *Z*, and the block diagonal structure of *D*, it can be seen that:

33 $$\begin{aligned} V= I+ZDZ'=I+\sum _{k=1}^{r}\sum _{j=1}^{l_r}Z_{(k,j)}D_kZ_{(k,j)}'. \end{aligned}$$

By substituting (33) into the second term of (2), and taking the partial derivative matrix with respect to $D_k$ using Lemma 1, the below can be obtained:

$$\begin{aligned} \frac{\partial }{\partial D_k}\bigg [\sigma ^{-2}e'V^{-1}e\bigg ]=\sigma ^{-2}\sum _{j=1}^{l_k}Z_{(k,j)}'V^{-1}ee'V^{-1}Z_{(k,j)}. \end{aligned}$$

Similarly, by substituting (33) into the third term of (2), and taking the partial derivative matrix with respect to $D_k$ using Lemma 2, the below can be obtained:

$$\begin{aligned} \frac{\partial }{\partial D_k}\big [\log |V|\big ]=\sum _{j=1}^{l_k}Z_{(k,j)}'V^{-1}Z_{(k,j)}. \end{aligned}$$

By combining the previous two derivative expressions, (32) is obtained.$\square$

Through applying the vectorization operator to (32), the following corollary is obtained. This is the result stated by (11) in Sect. 2.1.2.

#### Corollary 1

The partial derivative vector of the log-likelihood with respect to vec$(D_k)$ is given as:

$$\begin{aligned} \frac{\partial l(\theta )}{\partial \text {vec}(D_k)} = \frac{1}{2}\text {vec}\bigg (\sum _{j=1}^{l_k}Z_{(k,j)}'V^{-1}\bigg (\frac{ee'}{\sigma ^2}-V\bigg )V^{-1}Z_{(k,j)}\bigg ). \end{aligned}$$

Using Theorem 5.12 of Turkington (2013), which states that, in our notation:

$$\begin{aligned} \frac{dl(\theta )}{d\text {vec}(D_k)}={\mathcal {D}}_{q_k}{\mathcal {D}}_{q_k}'\frac{\partial l(\theta )}{\partial \text {vec}(D_k)}, \end{aligned}$$

the following corollary is now obtained.

#### Corollary 2

The total derivative vector of the log-likelihood with respect to vec($D_k$) is given as:

$$\begin{aligned}&\frac{d l(\theta )}{d \text {vec}(D_k)} \\&\quad =\frac{1}{2}{\mathcal {D}}_{q_k}{\mathcal {D}}'_{q_k}\text {vec}\bigg (\sum _{j=1}^{l_k}Z_{(k,j)}'V^{-1}\bigg (\frac{ee'}{\sigma ^2}-V\bigg )V^{-1}Z_{(k,j)}\bigg ). \end{aligned}$$

Finally, by noting that the vectorization and half-vectorization operators satisfy $\text {vec}(D_k)={\mathcal {D}}^+_{q_k}\text {vech}(D_k)$, the following corollary is obtained. This is the result stated by (7) in Sect. 2.1.1.

#### Corollary 3

The total derivative vector of the log-likelihood with respect to vech$(D_k)$ is given as:

$$\begin{aligned}&\frac{d l(\theta )}{d \text {vech}(D_k)} \\&\quad =\frac{1}{2}{\mathcal {D}}'_{q_k}\text {vec}\bigg (\sum _{j=1}^{l_k}Z_{(k,j)}'V^{-1}\bigg (\frac{ee'}{\sigma ^2}-V\bigg )V^{-1}Z_{(k,j)}\bigg ). \end{aligned}$$

### Fisher Information matrix

In this appendix, we provide derivations of the components of the Fisher Information matrix of $\theta ^f$ which relate to $D_k$, for some factor $f_k$. To derive these results, we will follow a similar argument to that of Demidenko (2013) in the single-factor setting. The derivation of the elements ${\mathcal {I}}^f_\beta$, ${\mathcal {I}}^f_{\beta ,\sigma ^2}$ and ${\mathcal {I}}^f_{\sigma ^2}$ for the multi-factor LMM is identical to that used for the single factor LMM given in Demidenko (2013) and, therefore, will not be repeated here. Theorems 2 and 3 provide derivation of equation (12) and Theorem 4 provides derivation of equation (13). Following this, Corollaries 4–6 detail the derivation of equations (9) and (10).

#### Theorem 2

For any arbitrary integer *k* between 1 and *r*, the covariance of the partial derivatives of $l(\theta ^f)$ with respect to $\beta$ and vec$(D_k)$ is given by:

$$\begin{aligned} {\mathcal {I}}^f_{\beta ,\text {vec}(D_k)}=\text {cov}\bigg (\frac{\partial l(\theta ^f)}{\partial \beta },\frac{\partial l(\theta ^f)}{\partial \text {vec}(D_k)}\bigg )={\mathbf {0}}_{p,q_k^2}. \end{aligned}$$

#### Proof

First, let *u* and $T_{(k,j)}$ denote the following quantities:

34 $$\begin{aligned} u = \sigma ^{-1}V^{-\frac{1}{2}}e, \quad T_{(k,j)}=Z_{(k,j)}'V^{-\frac{1}{2}}. \end{aligned}$$

As $e\sim N(0, \sigma ^2 V)$, it follows that $u\sim N(0,I_n)$. Let *c* be an arbitrary column vector of length $q_k$. Noting that the derivative of the log-likelihood function has mean zero, the below can be seen to be true:

$$\begin{aligned} \text {cov}\bigg (\frac{\partial l(\theta | y)}{\partial \beta },c'\frac{\partial l(\theta | y)}{\partial \text {vec}(D_k)}c \bigg ) = {\mathbb {E}}\bigg [ \frac{\partial l(\theta | y)}{\partial \beta }c'\frac{\partial l(\theta | y)}{\partial \text {vec}(D_k)}c\bigg ]. \end{aligned}$$

By rewriting in terms of *u* and $T_{(k,j)}$, and noting that ${\mathbb {E}}[u]=0$, the right hand side of the above can be seen to simplify to:

$$\begin{aligned} \begin{aligned}&{\mathbb {E}}\bigg [\sigma ^{-1}XV^{-\frac{1}{2}}u c'\bigg (\frac{1}{2}\sum _{j=1}^{l_k}(T_{(k,j)}u)(T_{(k,j)}u)'\bigg )c\bigg ] \\&\quad =\frac{1}{2}\sigma ^{-1}XV^{-\frac{1}{2}}\sum _{j=1}^{l_k}{\mathbb {E}}\bigg [u c'T_{(k,j)}uu'\bigg ]T_{(k,j)}'c={\mathbf {0}}_{p,q_k^2}. \end{aligned} \end{aligned}$$

That the above is equal to a matrix of zeros follows directly from the third moment of the Normal distribution being 0. The result of the theorem now follows. $\square$

#### Theorem 3

For any arbitrary integer *k* between 1 and *r*, the covariance of the partial derivatives of $l(\theta ^f)$ with respect to $\sigma ^2$ and vec$(D_k)$ is given by:

$$\begin{aligned} \begin{aligned}&{\mathcal {I}}^f_{\sigma ^2, \text {vec}(D_k)}=\text {cov}\bigg (\frac{\partial l(\theta ^f)}{\partial \sigma ^2},\frac{\partial l(\theta ^f)}{\partial \text {vec}(D_k)}\bigg ) \\&\quad = \frac{1}{2\sigma ^2}\text {vec}'\bigg (\sum _{j=1}^{l_k}Z'_{(k,j)}V^{-1}Z_{(k,j)}\bigg ). \end{aligned} \end{aligned}$$

#### Proof

To begin, we rewrite the covariance in Theorem 3 in terms of (34) and remove constant terms to obtain:

$$\begin{aligned} \frac{1}{4\sigma ^2}\text {cov}\bigg (u'u,\text {vec}\bigg (\sum _{j=1}^{l_k}(T_{(k,j)}u)(T_{(k,j)}u)'\bigg )\bigg ). \end{aligned}$$

Noting that the Kronecker product satisfies the property vec$(aa')=a\otimes a$, for all column vectors *a*, we obtain that the above is equal to:

$$\begin{aligned} \frac{1}{4\sigma ^2}\text {cov}\bigg (u'u,\sum _{j=1}^{l_k}\bigg [(T_{(k,j)}u)\otimes (T_{(k,j)}u)\bigg ]\bigg ). \end{aligned}$$

We now note that the Kronecker product satisfies vec$(ABC)=(C'\otimes A)$vec(*B*) for arbitrary matrices *A*, *B* and *C* of appropriate dimensions. Utilizing this, applying the mixed product property of the Kronecker product and then moving constant values outside of the covariance function now gives:

$$\begin{aligned} \frac{1}{4\sigma ^2}\text {vec}'(I_n)\text {cov}(u \otimes u)\sum _{j=1}^{l_k}\bigg [(T_{(k,j)}\otimes T_{(k,j)})\bigg ]^{'}. \end{aligned}$$

Noting that $u \sim N(0,I_n)$ we now employ a result from Magnus and Neudecker (1986) which states that $\text {cov}(u \otimes u)=2N_n$. Substituting this result into the previous expression gives the below:

$$\begin{aligned} \frac{1}{2\sigma ^2}\text {vec}'(I_n)N_n\sum _{j=1}^{l_k}\bigg [(T_{(k,j)}\otimes T_{(k,j)})\bigg ]'. \end{aligned}$$

By a result given in Magnus and Neudecker (1986), the matrix $N_n$ satisfies $(A \otimes A)N_k = N_n(A \otimes A)$ for all *A*, *n* and *k* such that the resulting matrix multiplications are well defined. Applying this result to the above expression, and again using the relationship vec$(ABC)=(C'\otimes A)$vec(*B*), the above can be seen to reduce to:

$$\begin{aligned} \frac{1}{2\sigma ^2}\text {vec}'\bigg (\sum _{j=1}^{l_k}T_{(k,j)}T_{(k,j)}'\bigg )N_{q_k}. \end{aligned}$$

By the definition of $T_{(k,j)}$, and noting that the matrix $N_{q_k}$ satisfies the relationship $\text {vec}'(A)N_{q_k}=\text {vec}'(A)$ for all appropriately sized symmetric matrices *A*, the result now follows. $\square$

#### Theorem 4

For any arbitrary integers $k_1$ and $k_2$ between 1 and *r*, the covariance of the partial derivatives of $l(\theta ^f)$ with respect to vec$(D_{k_1})$ and vec$(D_{k_2})$ is given by:

$$\begin{aligned} \begin{aligned}&{\mathcal {I}}^f_{\text {vec}(D_{k_1}),\text {vec}(D_{k_2})}=\text {cov}\bigg (\frac{\partial l(\theta ^f)}{\partial \text {vec}(D_{k_1})},\frac{\partial l(\theta ^f)}{\partial \text {vec}(D_{k_2})}\bigg )\\&\quad =\frac{1}{2}N_{q_{k_1}}\sum _{j=1}^{l_{k_2}}\sum _{i=1}^{l_{k_1}}(Z'_{(k_1,i)}V^{-1}Z_{(k_2,j)}\otimes Z'_{(k_1,i)}V^{-1}Z_{(k_2,j)}). \end{aligned} \end{aligned}$$

#### Proof

We begin by substituting the result of Corollary 1 into the covariance expression appearing in Theorem 4. By converting to the notation introduced in (34) and removing constant terms, the below is obtained:

$$\begin{aligned} \begin{aligned}&\frac{1}{4}\text {cov}\bigg (\sum _{j=1}^{l_{k_1}}\text {vec}\bigg [(T_{(k_1,j)}u)(T_{(k_1,j)}u)'\bigg ],\\&\quad \sum _{j=1}^{l_{k_2}}\text {vec}\bigg [(T_{(k_2,j)}u)(T_{(k_2,j)}u)'\bigg ]\bigg ). \end{aligned} \end{aligned}$$

Through similar arguments to those used in the proof of the previous theorem, Theorem 3, the above can be seen to be equal to:

$$\begin{aligned} \frac{1}{2}N_{q_{k_1}}\sum _{j=1}^{l_{k_2}}\sum _{i=1}^{l_{k_1}}\bigg [(T_{(k_1,i)}T_{(k_2,j)}')\otimes (T_{(k_1,i)}T_{(k_2,j)}')\bigg ]. \end{aligned}$$

From the definition of $T_{(k,j)}$, it can be seen that the above is equal to the result of Theorem 4. $\square$

We now turn attention to the derivation of (9) and (10). As in the proofs of Corollaries 2 and 3 of Appendix 6.1, we begin by noting that:

$$\begin{aligned} \begin{aligned} \frac{dl(\theta )}{d\text {vech}(D_k)}&= {\mathcal {D}}^+_{q_k}\frac{dl(\theta )}{d\text {vec}(D_k)} ={\mathcal {D}}^+_{q_k}{\mathcal {D}}_{q_k}{\mathcal {D}}_{q_k}'\frac{\partial l(\theta )}{\partial \text {vec}(D_k)}\\&={\mathcal {D}}_{q_k}'\frac{\partial l(\theta )}{\partial \text {vec}(D_k)}, \end{aligned} \end{aligned}$$

where the first equality follows from the definition of the duplication matrix and the second equality follows from Theorem 5.12 of Turkington (2013). Applying the above identity to Theorems 2, 3 and 4 and moving the matrix ${\mathcal {D}}_{q_k}'$ outside the covariance function in each, leads to the following three corollaries which, when taken in combination, provide equations (9) and (10).

#### Corollary 4

For any arbitrary integer *k* between 1 and *r*, the covariance of the total derivatives of $l(\theta ^h)$ with respect to $\beta$ and vech$(D_k)$ is given by:

$$\begin{aligned} {\mathcal {I}}^h_{\beta ,\text {vech}(D_k)}={\mathbf {0}}_{p,q_k(q_k+1)/2}. \end{aligned}$$

#### Corollary 5

For any arbitrary integer *k* between 1 and *r*, the covariance of the total derivatives of $l(\theta ^h)$ with respect to $\sigma ^2$ and vech$(D_k)$ is given by:

$$\begin{aligned} {\mathcal {I}}^h_{\sigma ^2, \text {vech}(D_k)}= \frac{1}{2\sigma ^2}\text {vec}'\bigg (\sum _{j=1}^{l_k}Z'_{(k,j)}V^{-1}Z_{(k,j)}\bigg ){\mathcal {D}}_{q_k}. \end{aligned}$$

#### Corollary 6

For any arbitrary integers $k_1$ and $k_2$ between 1 and *r*, the covariance of the total derivatives of $l(\theta ^h)$ with respect to vech$(D_{k_1})$ and vech$(D_{k_2})$ is given by:

$$\begin{aligned} \begin{aligned}&{\mathcal {I}}^h_{\text {vech}(D_{k_1}),\text {vech}(D_{k_2})}\\&\quad =\frac{1}{2}{\mathcal {D}}'_{q_{k_1}}\sum _{j=1}^{l_{k_2}}\sum _{i=1}^{l_{k_1}}(Z'_{(k_1,i)}V^{-1}Z_{(k_2,j)}\otimes Z'_{(k_1,i)}V^{-1}Z_{(k_2,j)}){\mathcal {D}}_{q_{k_2}}. \end{aligned} \end{aligned}$$

We note that the results of Corollaries 4, 5 and 6 do not contain the matrix $N_{q_k}$, which appears in the corresponding theorems (Theorems 2, 3 and 4). This is due to another result of Magnus and Neudecker (1986), which states that ${\mathcal {D}}'_{k}N_k={\mathcal {D}}'_{k}$ for any integer *k*. This concludes the derivations of Fisher Information matrix expressions given in Sects. 2.1.1–2.1.4.

### Restricted maximum likelihood estimation

In this appendix, we describe how the methods from Sect. 2.1 may be adapted to use an alternative likelihood criteria: the criteria employed by Restricted Maximum Likelihood (ReML) estimation. A well-documented issue for ML estimation is that the variance estimates produced using ML are biased. ReML addresses this issue by maximizing the log-likelihood function of the residual vector, *e*, instead of the response vector, *Y*. Neglecting constant terms, the restricted maximum log-likelihood function, $l_R$, is given by:

$$\begin{aligned} l_R(\theta ^h)=l(\theta ^h)-\frac{1}{2}\bigg ( -p\log (\sigma ^2)+\log |X'V^{-1}X| \bigg ), \end{aligned}$$

where $l(\theta ^h)$ is given in (2). To derive the ReML-based FS algorithm, akin to that described in Sect. 2.1.1, the following adjustments to the score vectors given by (6) and (7) must be used.

$$\begin{aligned} \begin{aligned}&\frac{dl_R(\theta ^h)}{d\beta } = \frac{dl(\theta ^h)}{d\beta }, \quad \frac{dl_R(\theta ^h)}{d\sigma ^2}=\frac{dl(\theta ^h)}{d\sigma ^2}+\frac{1}{2}p\sigma ^{-2},\\&\frac{dl_R(\theta ^h)}{d\text {vech}(D_k)}=\frac{dl(\theta ^h)}{d\text {vech}(D_k)}+\\&\frac{1}{2}{\mathcal {D}}'_{q_k}\text {vec}\bigg (\sum _{j=1}^{l_k}Z_{(k,j)}'V^{-1}X(X'V^{-1}X)^{-1}X'V^{-1}Z_{(k,j)}\bigg ). \end{aligned} \end{aligned}$$

The latter result can be derived through similar means to that of Theorem 1 and Corollaries 1–3. Derivation of the ReML score vectors of $\beta$ and $\sigma ^2$ can be found in Demidenko (2013) where proofs may be found for the single-factor LMM. As these proofs do not depend on the number of factors in the model, they can be seen to also apply in the multi-factor LMM setting without further adjustment.

Due to the asymptotic equivalence of the ML and ReML estimates, the parameter estimates produced by ML and ReML have the same asymptotic covariance matrix. Consequently, the Fisher Information matrix for the parameter estimates produced by ReML, which is the inverse of the asymptotic covariance matrix, is identical to that of the parameter estimates produced by ML. As a result, the ReML-based FS algorithm utilizes the Fisher Information matrix specified by (8)-(10) and requires no further derivation. To summarize, the ReML-based FS algorithm for the multi-factor LMM is almost identical to that given in Algorithm 1. The only adaptations required occur on line 3 of Algorithm 1 where the score vectors must be substituted for their ReML counterparts, provided above. Analogous adjustments can be made for the algorithms presented in Sects. 2.1.2–2.1.5.

### Cholesky Fisher Scoring

In this appendix, we prove the identity stated by the below theorem, Theorem 5, which was utilized in Sect. 2.1.5.

#### Theorem 5

Let $D_k$ be a square symmetric positive-definite matrix with Cholesky decomposition given by $D_k=\Lambda _k\Lambda _k'$. The below expression gives the derivative of vech$(D_k)$ with respect to vech$(\Lambda _k)$.

$$\begin{aligned} \frac{\partial \text {vech}(D_k)}{\partial \text {vech}(\Lambda _k)}={\mathcal {L}}_{q_k}(\Lambda '_k \otimes I_{q_k})(I_{q_k^2}+K_{q_k}){\mathcal {D}}_{q_k}. \end{aligned}$$

#### Proof

By the chain rule for vector-valued functions, as stated by Turkington (2013), the derivative in Theorem 5 can be expanded in the following manner:

$$\begin{aligned} \frac{\partial \text {vech}(D_k)}{\partial \text {vech}(\Lambda _k)}=\frac{\partial \text {vec}(\Lambda _k)}{\partial \text {vech}(\Lambda _k)}\frac{\partial \text {vec}(D_k)}{\partial \text {vec}(\Lambda _k)}\frac{\partial \text {vech}(D_k)}{\partial \text {vec}(D_k)}. \end{aligned}$$

The first and third derivatives in above the product are given by Theorems 5.9 and 5.10 of Turkington (2013), respectively, which state:

$$\begin{aligned} \frac{\partial \text {vec}(\Lambda _k)}{\partial \text {vech}(\Lambda _k)}={\mathcal {L}}_{q_k}, \quad \frac{\partial \text {vech}(D_k)}{\partial \text {vec}(D_k)}={\mathcal {D}}_{q_k}. \end{aligned}$$

The second derivative in the product is given by a result of Magnus and Neudecker (1999), which states that:

$$\begin{aligned} \frac{\partial \text {vec}(\Lambda _k\Lambda _k')}{\partial \text {vec}(\Lambda _k)}=(\Lambda '_k \otimes I_{q_k})(I_{q_k^2}+K_{q_k}). \end{aligned}$$

Combining the above derivatives yields the desired result. $\square$

### Derivative of $S^2({\hat{\eta }}^h)$ with respect to vech$({\hat{D}}_k)$

In this appendix, proof of the derivative result which was given in Sect. 2.5 is provided. Following this, an extension of this result is provided for models containing constrained covariance matrices of the form described in Sect. 2.4.

#### Theorem 6

Define ${\hat{\eta }}^h$ as in section 2.5 and define the function $S^2({\hat{\eta }}^h)$ by:

$$\begin{aligned} S^2({\hat{\eta }}^h)=\sigma ^2L(X'{\hat{V}}^{-1}X)^{-1}L'. \end{aligned}$$

The derivative of $S^2({\hat{\eta }}^h)$ with respect to the half vectorization of ${\hat{D}}_k$ is given by:

$$\begin{aligned} \frac{d S^2({\hat{\eta }}^h)}{d \text {vech}({\hat{D}}_k)} = {\hat{\sigma }}^{2}{\mathcal {D}}_{q_k}'\bigg (\sum _{j=1}^{l_k}{\hat{B}}_{(k,j)}\otimes {\hat{B}}_{(k,j)}\bigg ), \end{aligned}$$

where $B_{(k,j)}$ is given by ${\hat{B}}_{(k,j)} = Z_{(k,j)}'{\hat{V}}^{-1}X(X'{\hat{V}}^{-1}X)^{-1}L'$.

#### Proof

By the chain rule for vector valued functions, as stated by Turkington (2013), and by noting that the function $S^2({\hat{\eta }}^h)$ outputs a $(1 \times 1)$ scalar value, it can be seen that:

$$\begin{aligned} \begin{aligned}&\frac{\partial S^2({\hat{\eta }}^h)}{\partial \text {vec}({\hat{D}}_k)}={\hat{\sigma }}^2\frac{\partial \big (L(X'{\hat{V}}^{-1}X)^{-1}L'\big )}{\partial \text {vec}({\hat{D}}_k)} \\&= {\hat{\sigma }}^2 \frac{\partial \text {vec}({\hat{V}})}{\partial \text {vec}({\hat{D}}_k)} \frac{\partial \text {vec}({\hat{V}}^{-1})}{\partial \text {vec}({\hat{V}})} \frac{\partial \text {vec}(X'{\hat{V}}^{-1}X)}{\partial \text {vec}({\hat{V}}^{-1})} \frac{\partial \big (L(X'{\hat{V}}^{-1}X)^{-1}L'\big )}{\partial \text {vec}(X'{\hat{V}}^{-1}X)}. \end{aligned} \end{aligned}$$

Using the expansion given in (33) the first derivative in the product is evaluated to the below.

$$\begin{aligned} \frac{\partial \text {vec}({\hat{V}})}{\partial \text {vec}({\hat{D}}_k)}=\sum _{j=1}^{l_k}Z'_{(k,j)}\otimes Z'_{(k,j)}. \end{aligned}$$

For the second derivative in the product, we apply result (4.17) from Turkington (2013), which states:

$$\begin{aligned} \frac{\partial \text {vec}({\hat{V}}^{-1})}{\partial \text {vec}({\hat{V}})}=-{\hat{V}}^{-1}\otimes {\hat{V}}^{-1}. \end{aligned}$$

For the third term of the product, a result stated in Chapter 5.7 of Turkington (2013) gives the following:

$$\begin{aligned} \frac{\partial \text {vec}(X'{\hat{V}}^{-1}X)}{\partial \text {vec}({\hat{V}}^{-1})}=X \otimes X. \end{aligned}$$

By a variant of (4.17) from Turkington (2013), given on the line following the statement of (4.17), the below is obtained:

$$\begin{aligned}&\frac{\partial \big (L(X'{\hat{V}}^{-1}X)^{-1}L'\big )}{\partial \text {vec}(X'{\hat{V}}^{-1}X)} \\&\quad =-((X'{\hat{V}}^{-1}X)^{-1}L')\otimes ((X'{\hat{V}}^{-1}X)^{-1}L'). \end{aligned}$$

Following this, application of the mixed product property of the Kronecker product and multiplication by the transposed duplication matrix yields the result of Theorem 6. $\square$

#### Theorem 7

Define $\rho _{{\hat{D}}}$ and ${\mathcal {C}}$ as in Sect. (2.4) and ${\hat{B}}_{(k,j)}$ and ${\hat{\eta }}^h$ as in Theorem 6. The derivative of $S^2({\hat{\eta }}^h)$ with respect to $\rho _{{\hat{D}}}$ is given by:

$$\begin{aligned} \frac{d S^2({\hat{\eta }}^h)}{d \rho _{{\hat{D}}}} = \sigma ^{2}{\mathcal {C}}\hat{{\mathcal {B}}}, \end{aligned}$$

where $\hat{{\mathcal {B}}}$ is defined by:

$$\begin{aligned} \hat{{\mathcal {B}}}= \left[ \bigg (\sum _{j=1}^{l_1}{\hat{B}}_{(1,j)}'\otimes {\hat{B}}_{(1,j)}'\bigg ),\ldots ,\bigg (\sum _{j=1}^{l_r}{\hat{B}}_{(r,j)}'\otimes {\hat{B}}_{(r,j)}'\bigg )\right] '. \end{aligned}$$

#### Proof

From the proof of Theorem 6, it can be seen that the partial derivative of $S^2({\hat{\eta }}^h)$ with respect to vec$({\hat{D}}_k)$ is given by:

$$\begin{aligned} \frac{\partial S^2({\hat{\eta }}^h)}{\partial \text {vec}({\hat{D}}_k)} = {\hat{\sigma }}^{2}\sum _{j=1}^{l_k}{\hat{B}}_{(k,j)}\otimes {\hat{B}}_{(k,j)}. \end{aligned}$$

By defining $v({\hat{D}})$ as in Sect. 2.4, it can be seen from the above that the partial derivative of $S^2({\hat{\eta }}^h)$ with respect to $v({\hat{D}})$ is ${\hat{\sigma }}^{2}\hat{{\mathcal {B}}}$. By the chain rule for vector valued functions, as stated by Turkington (2013), the below can now be obtained;

$$\begin{aligned} \frac{d S^2({\hat{\eta }}^h)}{d \rho _{{\hat{D}}}} = \frac{\partial v({\hat{D}})}{\partial \rho _{{\hat{D}}}}\frac{\partial S^2({\hat{\eta }}^h)}{\partial v({\hat{D}})}={\mathcal {C}}\frac{\partial S^2({\hat{\eta }}^h)}{\partial v({\hat{D}})}, \end{aligned}$$

where the second equality follows from the definition of ${\mathcal {C}}$. Substituting the partial derivative of $S^2({\hat{\eta }}^h)$ with respect to $\rho _{{\hat{D}}}$ into the above completes the proof. $\square$

### The ACE model

#### Specification of random effects

In this appendix, we provide detail on how the random effects vector, *b*, and covariance matrix, *D*, are defined for the ACE model. To do so, we first describe the covariance of the random terms $\gamma _{k,j,i}$ from Sect. 4.1.2. Following this, we use the $\gamma _{k,j,i}$ terms to construct the random effects vector, *b*. To simplify notation, we assume that for each family structure type, subjects within family units which exhibit such a structure are ordered consistently. For example, if the family structure describes “families containing one twin-pair and one half-sibling”, we assume that the members of every family who exhibit such a structure are given in the same order: (twin, twin, half-sibling).

As noted in Sect. 4.1.2, $\gamma _{k,j,i}$ models the within-“family unit” covariance. As such, $\text {cov}(\gamma _{k,j_1,i},\gamma _{k,j_2,i})=0$, for any two distinct family units, $j_1\ne j_2$. Within any individual family unit (e.g. family unit *j* of structure type *k*), the random effects $\{\gamma _{k,j,i}\}_{i \in \{1,\ldots , q_k\}}$ are defined to have the below covariance matrix:

$$\begin{aligned} \begin{aligned} \text {cov}\left( \begin{bmatrix} \gamma _{k,j,1}&...&\gamma _{k,j,q_k} \end{bmatrix}'\right) = \sigma ^2_a{\mathbf {K}}^a_k + \sigma ^2_c{\mathbf {K}}^c_k, \end{aligned} \end{aligned}$$

where ${\mathbf {K}}^a_k$ and ${\mathbf {K}}^c_k$ are the known, predefined kinship (additive genetic) and common environmental effects matrices, respectively (see Supplementary Material Section S16.1 for more details). The random effects vector, *b*, is constructed by vertical concatenation of the random $\gamma _{k,i,j}$ terms, i.e. $b = [\gamma _{1,1,1},\gamma _{1,1,2},\ldots ,\gamma _{r,l_r,q_r}]'$. To derive an expression for the covariance matrix of *b*, we note from equation (1) that $\sigma ^2_eD$ is equal to cov(*b*). Equating this with the previous expression, it may now be seen that *D* is block diagonal, with its *k*th unique diagonal block given by $D_k = \sigma ^{-2}_e(\sigma ^2_a{\mathbf {K}}^a_k + \sigma ^2_c{\mathbf {K}}^c_k)$.

#### Constrained optimization for the ACE model

In this appendix, a brief overview of the constrained optimization procedure which was adopted for parameter estimation of the ACE model is provided. A derivation of the constraint matrix, ${\mathcal {C}}$ is then provided by Theorem 8.

To perform parameter estimation for the ACE model, an approach based on the ReML criterion described in Appendix 6.3 and the constrained covariance structure methods outlined in Sect. 2.4 was adopted. The resulting optimization procedure was performed in terms of the parameter vector $\theta ^{ACE}=(\beta , \tau _a, \tau _c, \sigma ^2_e)$, where $\tau _a=\sigma ^{-1}_e\sigma _a$ and $\tau _c=\sigma ^{-1}_e\sigma _c$. $\beta$ and $\sigma ^2_e$ were updated according to the GLS update rules provided by equation (16), while updates for the parameter vector $[\tau _a, \tau _c]'$ were performed via a Fisher Scoring update rule. The Fisher Scoring update rule employed was of the form (3) with $\theta$ substituted for $[\tau _a, \tau _c]'$. To obtain the gradient vector and information matrix required to perform this update step, a constraint-based approach (c.f. Sect. 2.4) was employed. An expression for the required constraint matrix, ${\mathcal {C}}$, alongside derivation, is given by Theorem 8 below.

##### Theorem 8

For the ACE model, the constraint matrix (Jacobian) which maps a partial derivative vector taken with respect to *v*(*D*) to a total derivative vector taken with respect to $\tau =[\tau _a,\tau _c]'$ is given by:

$$\begin{aligned} \begin{aligned} {\mathcal {C}} = \bigg (\mathbb {1}_{(1,r)} \otimes \begin{bmatrix} 2\tau _a &{} 0 \\ 0 &{} 2\tau _c \end{bmatrix}\bigg ) \bigg (\bigoplus _{k=1}^r \begin{bmatrix} \text {vec}({\mathbf {K}}^a_k)' \\ \text {vec}({\mathbf {K}}^c_k)' \end{bmatrix}\bigg ), \end{aligned} \end{aligned}$$

where $\oplus$ represents the direct sum.

##### Proof

We first define ${\tilde{\tau }}_{a,1},\ldots {\tilde{\tau }}_{a,r}$ and ${\tilde{\tau }}_{c,1},\ldots {\tilde{\tau }}_{c,r}$ as variables which satisfy the below expressions, for all $k \in \{1,\ldots ,r\}$.

$$\begin{aligned}&\tau _a = {\tilde{\tau }}_{a,k},\\&\quad \tau _c = {\tilde{\tau }}_{c,k},\quad \text {vec}(D_k) = {\tilde{\tau }}^2_{a,k}\text {vec}({\mathbf {K}}^a_k) + {\tilde{\tau }}^2_{c,k}\text {vec}({\mathbf {K}}^c_k). \end{aligned}$$

We now define the vector ${\tilde{\tau }}=[{\tilde{\tau }}_{a,1},{\tilde{\tau }}_{c,1}, \ldots , {\tilde{\tau }}_{a,r},{\tilde{\tau }}_{c,r}]'$. By the chain rule and definition of ${\mathcal {C}}$, it can be seen that:

35 $$\begin{aligned} {\mathcal {C}}=\frac{d v(D)}{d\tau }=\frac{\partial {\tilde{\tau }}}{\partial \tau }\frac{\partial v(D)}{\partial {\tilde{\tau }}}. \end{aligned}$$

The first partial derivative in the product is given by:

$$\begin{aligned} \frac{\partial {\tilde{\tau }}}{\partial \tau } = \begin{bmatrix} 1 &{} 0 &{} 1 &{} 0 &{} 1 &{} ...&{} 0 \\ 0 &{} 1 &{} 0 &{} 1 &{} 0 &{} ...&{} 1 \\ \end{bmatrix} = \mathbb {1}_{(1,r)} \otimes I_2. \end{aligned}$$

To evaluate the second derivative in the product, we first consider the partial derivative of vec$(D_k)$ with respect to $[{\tilde{\tau }}_{a,k},{\tilde{\tau }}_{c,k}]'$ for arbitrary $k \in \{1,\ldots ,r\}$. From the definitions of ${\tilde{\tau }}_{a,k}$ and ${\tilde{\tau }}_{c,k}$, it can be seen that:

$$\begin{aligned} \begin{aligned} \frac{\partial \text {vec}(D_k)}{\partial [{\tilde{\tau }}_{a,k}, {\tilde{\tau }}_{c,k}]'}&=\begin{bmatrix} 2{\tilde{\tau }}_{a,k}\text {vec}({\mathbf {K}}^a_k)' \\ 2{\tilde{\tau }}_{c,k}\text {vec}({\mathbf {K}}^c_k)'\\ \end{bmatrix}. \end{aligned} \end{aligned}$$

By similar reasoning it can be seen, for arbitrary $k_1,k_2 \in \{1,\ldots ,r\}$, such that $k_1 \ne k_2$, that the below is true:

$$\begin{aligned} \frac{\partial \text {vec}(D_{k_1})}{\partial [{\tilde{\tau }}_{a,k_2}, {\tilde{\tau }}_{c,k_2}]'} = {\mathbf {0}}_{(2,q^2_{k_1})}, \end{aligned}$$

where ${\mathbf {0}}_{(2,q^2_{k_1})}$ is the $(2 \times q^2_{k_1})$ dimensional matrix of zero elements. By combining the above expressions and noting the definitions of *v*(*D*) and ${\tilde{\tau }}$, it can now be seen that the derivative of *v*(*D*) with respect to ${\tilde{\tau }}$ is given by the below.

$$\begin{aligned} \begin{aligned}&\begin{bmatrix} \begin{bmatrix} 2{\tilde{\tau }}_{a,1}\text {vec}({\mathbf {K}}^a_1)' \\ 2{\tilde{\tau }}_{c,1}\text {vec}({\mathbf {K}}^c_1)'\\ \end{bmatrix} &{} {\mathbf {0}}_{(2,q^2_2)} &{} ... &{} {\mathbf {0}}_{(2,q^2_r)} \\ {\mathbf {0}}_{(2,q^2_1)} &{}\begin{bmatrix} 2{\tilde{\tau }}_{a,2}\text {vec}({\mathbf {K}}^a_2)' \\ 2{\tilde{\tau }}_{c,2}\text {vec}({\mathbf {K}}^c_2)'\\ \end{bmatrix} &{} ... &{} {\mathbf {0}}_{(2,q^2_r)} \\ \vdots &{} \vdots &{} \ddots &{} \vdots \\ {\mathbf {0}}_{(2,q^2_1)} &{} {\mathbf {0}}_{(2,q^2_2)} &{} ... &{} \begin{bmatrix} 2{\tilde{\tau }}_{a,r}\text {vec}({\mathbf {K}}^a_r)' \\ 2{\tilde{\tau }}_{c,r}\text {vec}({\mathbf {K}}^c_r)'\\ \end{bmatrix}\\ \end{bmatrix} \\&\quad = \bigoplus _{k=1}^r \begin{bmatrix} 2{\tilde{\tau }}_{a,k}\text {vec}({\mathbf {K}}^a_k)' \\ 2{\tilde{\tau }}_{c,k}\text {vec}({\mathbf {K}}^c_k)' \end{bmatrix}. \\ \end{aligned} \end{aligned}$$

By substituting the above partial derivative results into (35), substituting $\tau _a = {\tilde{\tau }}_{a,k}$ and $\tau _c = {\tilde{\tau }}_{c,k}$ and rearranging, the result stated in Theorem 8 can now be obtained. $\square$

## Supplementary Information

Below is the link to the electronic supplementary material.

Supplementary material 1 (pdf 205 KB)

## Data Availability

Data were provided in part by the Human Connectome Project, WU-Minn Consortium (Principal Investigators: David Van Essen and Kamil Ugurbil; 1U54MH091657) funded by the 16 NIH Institutes and Centers that support the NIH Blueprint for Neuroscience Research; and by the McDonnell Center for Systems Neuroscience at Washington University. The longitudinal evaluation of school change and performance (LESCP) dataset employed in Sects. 4.1.1 and 4.2.1 is publicly available and can be found, for example, as one of the example datasets included in the hierarchical linear models (HLM) software package (Raudenbush and Bryk 2002).

## Code availability

All code used to generate the results of the simulations and real data examples presented in Sects. 3.1–4.2.2 is publicly available and can be found in the below GitHub repository: https://github.com/TomMaullin/LMMPaper Included in this repository are detailed notebooks demonstrating how the code may be executed in order to reproduce the results presented in this work.
