# Supplementary material for: Fisher Scoring for crossed factor linear mixed models
Source: Stat Comput. 2021 Jul 19;31(5):53. doi: 10.1007/s11222-021-10026-6 (PMC8550113; doi:10.1007/s11222-021-10026-6)
Supplement: Supplementary file 1 — Supplementary material 1 (pdf 205 KB) [file 11222_2021_10026_MOESM1_ESM.pdf]

Fisher Scoring for crossed factor Linear Mixed Models: Supplementary material

Thomas Maullin-Sapey*,[1] and Thomas E. Nichols[1]

[1] Big Data Institute, Li Ka Shing Centre for Health Information and Discovery, Old Road Campus, Oxford, OX3 7LF, UK
*Corresponding Author: Thomas, TM, Maullin-Sapey, Thomas.Maullin-Sapey@bdi.ox.ac.uk
Submission for Statistics and Computing

S1 Baseline truth for parameter estimation simulations

**Parameter**	Simulation Setting 1	Simulation Setting 2	Simulation Setting 3
β (Fixed effects parameters)	$\begin{bmatrix}5 & 4 & 3 & 2 & 1 & 0\end{bmatrix}'$	$\begin{bmatrix}5 & 4 & 3 & 2 & 1 & 0\end{bmatrix}'$	$\begin{bmatrix}5 & 4 & 3 & 2 & 1 & 0\end{bmatrix}'$
σ^2 (Fixed effects variance)	1	1	1
D_1 (Random effects covariance matrix for the 1st random factor):	$\begin{bmatrix}1 & 0\\0 & 1\end{bmatrix}$	$\begin{bmatrix}1 & 0.7 & 0.5\\0.7 & 0.9 & 0.6\\0.5 & 0.6 & 0.8\end{bmatrix}$	$\begin{bmatrix}1 & 0.75 & 0.5 & 0.25\\0.75 & 1 & 0.75 & 0.5\\0.5 & 0.75 & 1 & 0.75\\0.25 & 0.5 & 0.75 & 1\end{bmatrix}$
D_2 (Random effects covariance matrix for the 2nd random factor):	–	$\begin{bmatrix}1 & 0\\0 & 1\end{bmatrix}$	$\begin{bmatrix}1 & 0.7 & 0.5\\0.7 & 0.9 & 0.6\\0.5 & 0.6 & 0.8\end{bmatrix}$
D_3 (Random effects covariance matrix for the 3rd random factor):	–	–	$\begin{bmatrix}1 & 0\\0 & 1\end{bmatrix}$

Table S1: Parameters chosen as baseline truth for the parameter estimation simulations. Entries marked as '–' indicate that the design for the corresponding simulation setting did not include the specified parameter.

S2 MAE and MRD definition

Here, we formally define the Mean Absolute Error (MAE) and Mean Relative Difference (MRD) which were used in the main text as performance metrics for between-method comparison. For a model parameter γ, these were defined as:

$$MAE(\gamma) = \frac{1}{n_{sim}} \sum_i \|\hat{\gamma}_i - \bar{\gamma}_i\|_\infty,$$

$$MRD(\gamma) = \frac{1}{n_{sim}} \sum_i \left\| \frac{\hat{\gamma}_i - \bar{\gamma}_i}{\frac{1}{2}(\hat{\gamma}_i + \bar{\gamma}_i)} \right\|_\infty,$$

where n_{sim} is the total number of simulations run, $\hat{\gamma}_i$ is an estimate of the parameter vector γ obtained during the ith simulation using the parameter estimation method of interest, and $\bar{\gamma}_i$ is a baseline truth to which $\hat{\gamma}_i$ is to be compared.

S3 MAE results for ML estimation

Method ($\mathbf{m_1}$)	FS	FFS	SFS	FSFS	CSFS	lmer
Simulation 1						
$MAE_l(\beta)$	7.12×10^{-6}	7.12×10^{-6}	3.41×10^{-6}	3.41×10^{-6}	1.95×10^{-5}	-
	(1.08×10^{-5})	(1.08×10^{-5})	(8.01×10^{-6})	(8.01×10^{-6})	(1.74×10^{-5})	-
$MAE_t(\beta)$	1.28×10^{-1}	1.28×10^{-1}	1.28×10^{-1}	1.28×10^{-1}	1.28×10^{-1}	1.28×10^{-1}
	(8.39×10^{-2})	(8.39×10^{-2})	(8.39×10^{-2})	(8.39×10^{-2})	(8.39×10^{-2})	(8.39×10^{-2})
$MAE_l(\sigma^2D)$	2.12×10^{-5}	2.12×10^{-5}	1.96×10^{-5}	1.96×10^{-5}	9.07×10^{-5}	-
	(3.72×10^{-5})	(3.72×10^{-5})	(3.72×10^{-5})	(3.72×10^{-5})	(3.48×10^{-5})	-
$MAE_t(\sigma^2D)$	2.58×10^{-1}	2.58×10^{-1}	2.58×10^{-1}	2.58×10^{-1}	2.58×10^{-1}	2.58×10^{-1}
	(1.21×10^{-1})	(1.21×10^{-1})	(1.21×10^{-1})	(1.21×10^{-1})	(1.21×10^{-1})	(1.21×10^{-1})
Simulation 2						
$MAE_l(\beta)$	9.75×10^{-6}	9.75×10^{-6}	5.40×10^{-6}	5.40×10^{-6}	1.30×10^{-5}	-
	(1.38×10^{-5})	(1.38×10^{-5})	(1.25×10^{-5})	(1.25×10^{-5})	(1.69×10^{-5})	-
$MAE_t(\beta)$	9.77×10^{-2}	9.77×10^{-2}	9.77×10^{-2}	9.77×10^{-2}	9.77×10^{-2}	9.77×10^{-2}
	(5.92×10^{-2})	(5.92×10^{-2})	(5.92×10^{-2})	(5.92×10^{-2})	(5.92×10^{-2})	(5.92×10^{-2})
$MAE_l(\sigma^2D)$	1.05×10^{-4}	1.05×10^{-4}	1.04×10^{-4}	1.04×10^{-4}	1.08×10^{-4}	-
	(1.96×10^{-4})	(1.96×10^{-4})	(1.96×10^{-4})	(1.96×10^{-4})	(1.95×10^{-4})	-
$MAE_t(\sigma^2D)$	3.93×10^{-1}	3.93×10^{-1}	3.93×10^{-1}	3.93×10^{-1}	3.93×10^{-1}	3.93×10^{-1}
	(1.52×10^{-1})	(1.52×10^{-1})	(1.52×10^{-1})	(1.52×10^{-1})	(1.52×10^{-1})	(1.52×10^{-1})
Simulation 3						
$MAE_l(\beta)$	7.50×10^{-6}	7.50×10^{-6}	5.30×10^{-6}	5.30×10^{-6}	1.54×10^{-4}	-
	(9.08×10^{-6})	(9.08×10^{-6})	(8.06×10^{-6})	(8.06×10^{-6})	(5.61×10^{-4})	-
$MAE_t(\beta)$	8.34×10^{-2}	8.34×10^{-2}	8.34×10^{-2}	8.34×10^{-2}	8.34×10^{-2}	8.34×10^{-2}
	(3.99×10^{-2})	(3.99×10^{-2})	(3.99×10^{-2})	(3.99×10^{-2})	(3.99×10^{-2})	(3.99×10^{-2})
$MAE_l(\sigma^2D)$	4.08×10^{-4}	4.08×10^{-4}	4.08×10^{-4}	4.08×10^{-4}	2.96×10^{-3}	-
	(6.23×10^{-4})	(6.23×10^{-4})	(6.23×10^{-4})	(6.23×10^{-4})	(7.97×10^{-3})	-
$MAE_t(\sigma^2D)$	5.76×10^{-1}	5.76×10^{-1}	5.76×10^{-1}	5.76×10^{-1}	5.76×10^{-1}	5.76×10^{-1}
	(2.54×10^{-1})	(2.54×10^{-1})	(2.54×10^{-1})	(2.54×10^{-1})	(2.54×10^{-1})	(2.54×10^{-1})

Table S2: Average MAE values for the β and σ^2D parameter estimates, taken relative to the baseline truth used for simulation (t) and the parameter estimates produced by lmer (l). All reported results were obtained through averages taken from 1000 simulations performed for each simulation setting, with parameter estimation performed using the ML likelihood criteria. Empirical standard errors are also given in brackets underneath each entry in the table.

S4 MAE results for ReML estimation

**Method ($\mathbf{m_1}$)**	**FS**	**FFS**	**SFS**	**FSFS**	**CSFS**	**lmer**
Simulation 1						
$MAE_l(\beta)$	7.47×10^{-6}	7.47×10^{-6}	3.39×10^{-6}	3.39×10^{-6}	2.00×10^{-5}	-
	(1.14×10^{-5})	(1.14×10^{-5})	(8.18×10^{-6})	(8.18×10^{-6})	(1.66×10^{-5})	-
$MAE_t(\beta)$	1.21×10^{-1}	1.21×10^{-1}	1.21×10^{-1}	1.21×10^{-1}	1.21×10^{-1}	1.21×10^{-1}
	(8.10×10^{-2})	(8.10×10^{-2})	(8.10×10^{-2})	(8.10×10^{-2})	(8.10×10^{-2})	(8.10×10^{-2})
$MAE_l(\sigma^2 D)$	2.08×10^{-5}	2.08×10^{-5}	1.87×10^{-5}	1.87×10^{-5}	9.30×10^{-5}	-
	(4.12×10^{-5})	(4.12×10^{-5})	(4.13×10^{-5})	(4.13×10^{-5})	(4.13×10^{-5})	-
$MAE_t(\sigma^2 D)$	2.59×10^{-1}	2.59×10^{-1}	2.59×10^{-1}	2.59×10^{-1}	2.59×10^{-1}	2.59×10^{-1}
	(1.17×10^{-1})	(1.17×10^{-1})	(1.17×10^{-1})	(1.17×10^{-1})	(1.17×10^{-1})	(1.17×10^{-1})
Simulation 2						
$MAE_l(\beta)$	1.02×10^{-5}	1.02×10^{-5}	5.57×10^{-6}	5.57×10^{-6}	1.29×10^{-5}	-
	(1.18×10^{-5})	(1.18×10^{-5})	(9.62×10^{-6})	(9.62×10^{-6})	(1.43×10^{-5})	-
$MAE_t(\beta)$	9.37×10^{-2}	9.37×10^{-2}	9.37×10^{-2}	9.37×10^{-2}	9.37×10^{-2}	9.37×10^{-2}
	(5.25×10^{-2})	(5.25×10^{-2})	(5.25×10^{-2})	(5.25×10^{-2})	(5.25×10^{-2})	(5.25×10^{-2})
$MAE_l(\sigma^2 D)$	1.14×10^{-4}	1.14×10^{-4}	1.14×10^{-4}	1.14×10^{-4}	1.17×10^{-4}	-
	(2.24×10^{-4})	(2.24×10^{-4})	(2.24×10^{-4})	(2.24×10^{-4})	(2.23×10^{-4})	-
$MAE_t(\sigma^2 D)$	3.87×10^{-1}	3.87×10^{-1}	3.87×10^{-1}	3.87×10^{-1}	3.87×10^{-1}	3.87×10^{-1}
	(1.51×10^{-1})	(1.51×10^{-1})	(1.51×10^{-1})	(1.51×10^{-1})	(1.51×10^{-1})	(1.51×10^{-1})
Simulation 3						
$MAE_l(\beta)$	8.01×10^{-6}	8.01×10^{-6}	5.57×10^{-6}	5.57×10^{-6}	2.38×10^{-4}	-
	(1.21×10^{-5})	(1.21×10^{-5})	(1.10×10^{-5})	(1.10×10^{-5})	(1.18×10^{-3})	-
$MAE_t(\beta)$	8.61×10^{-2}	8.61×10^{-2}	8.61×10^{-2}	8.61×10^{-2}	8.61×10^{-2}	8.61×10^{-2}
	(4.15×10^{-2})	(4.15×10^{-2})	(4.15×10^{-2})	(4.15×10^{-2})	(4.15×10^{-2})	(4.15×10^{-2})
$MAE_l(\sigma^2 D)$	4.30×10^{-4}	4.30×10^{-4}	4.30×10^{-4}	4.30×10^{-4}	4.01×10^{-3}	-
	(8.40×10^{-4})	(8.40×10^{-4})	(8.40×10^{-4})	(8.40×10^{-4})	(1.42×10^{-2})	-
$MAE_t(\sigma^2 D)$	5.88×10^{-1}	5.88×10^{-1}	5.88×10^{-1}	5.88×10^{-1}	5.88×10^{-1}	5.88×10^{-1}
	(2.57×10^{-1})	(2.57×10^{-1})	(2.57×10^{-1})	(2.57×10^{-1})	(2.57×10^{-1})	(2.57×10^{-1})

Table S3: Average MAE values for the β and $\sigma^2 D$ parameter estimates, taken relative to the baseline truth used for simulation (t) and the parameter estimates produced by lmer (l). All reported results were obtained through averages taken from 1000 simulations performed for each simulation setting, with parameter estimation performed using the ReML likelihood criteria. Empirical standard errors are also given in brackets underneath each entry in the table.

S5 MRD results for ML estimation

**Method ($\mathbf{m_1}$)**	**FS**	**FFS**	**SFS**	**FSFS**	**CSFS**	**lmer**
Simulation 1						
$MRD_l(\beta)$	1.63×10^{-5}	1.63×10^{-5}	1.09×10^{-5}	1.09×10^{-5}	4.17×10^{-5}	-
	(1.79×10^{-4})	(1.79×10^{-4})	(1.13×10^{-4})	(1.13×10^{-4})	(3.75×10^{-4})	-
$MRD_t(\beta)$	4.17×10^{-2}	4.17×10^{-2}	4.17×10^{-2}	4.17×10^{-2}	4.17×10^{-2}	4.17×10^{-2}
	(2.09×10^{-2})	(2.09×10^{-2})	(2.09×10^{-2})	(2.09×10^{-2})	(2.09×10^{-2})	(2.09×10^{-2})
$MRD_l(\sigma^2D)$	5.18×10^{-4}	5.18×10^{-4}	4.63×10^{-4}	4.63×10^{-4}	2.81×10^{-3}	-
	(8.58×10^{-3})	(8.58×10^{-3})	(9.52×10^{-3})	(9.52×10^{-3})	(2.53×10^{-2})	-
$MRD_t(\sigma^2D)$	2.42×10^{-1}	2.42×10^{-1}	2.42×10^{-1}	2.42×10^{-1}	2.42×10^{-1}	2.42×10^{-1}
	(1.35×10^{-1})	(1.35×10^{-1})	(1.35×10^{-1})	(1.35×10^{-1})	(1.35×10^{-1})	(1.35×10^{-1})
Simulation 2						
$MRD_l(\beta)$	3.92×10^{-5}	3.92×10^{-5}	4.51×10^{-5}	4.51×10^{-5}	7.20×10^{-5}	-
	(3.15×10^{-4})	(3.15×10^{-4})	(4.46×10^{-4})	(4.46×10^{-4})	(6.62×10^{-4})	-
$MRD_t(\beta)$	3.73×10^{-2}	3.73×10^{-2}	3.73×10^{-2}	3.73×10^{-2}	3.73×10^{-2}	3.73×10^{-2}
	(1.85×10^{-2})	(1.85×10^{-2})	(1.85×10^{-2})	(1.85×10^{-2})	(1.85×10^{-2})	(1.85×10^{-2})
$MRD_l(\sigma^2D)$	5.35×10^{-4}	5.35×10^{-4}	5.34×10^{-4}	5.34×10^{-4}	5.65×10^{-4}	-
	(2.30×10^{-3})	(2.30×10^{-3})	(2.31×10^{-3})	(2.31×10^{-3})	(2.35×10^{-3})	-
$MRD_t(\sigma^2D)$	3.25×10^{-1}	3.25×10^{-1}	3.25×10^{-1}	3.25×10^{-1}	3.25×10^{-1}	3.25×10^{-1}
	(1.96×10^{-1})	(1.96×10^{-1})	(1.96×10^{-1})	(1.96×10^{-1})	(1.96×10^{-1})	(1.96×10^{-1})
Simulation 3						
$MRD_l(\beta)$	7.03×10^{-4}	7.03×10^{-4}	1.03×10^{-3}	1.03×10^{-3}	2.89×10^{-3}	-
	(1.25×10^{-2})	(1.25×10^{-2})	(2.47×10^{-2})	(2.47×10^{-2})	(3.90×10^{-2})	-
$MRD_t(\beta)$	3.96×10^{-2}	3.96×10^{-2}	3.96×10^{-2}	3.96×10^{-2}	3.95×10^{-2}	3.96×10^{-2}
	(2.09×10^{-2})	(2.09×10^{-2})	(2.09×10^{-2})	(2.09×10^{-2})	(2.09×10^{-2})	(2.09×10^{-2})
$MRD_l(\sigma^2D)$	2.12×10^{-3}	2.12×10^{-3}	2.12×10^{-3}	2.12×10^{-3}	4.99×10^{-3}	-
	(1.92×10^{-2})	(1.92×10^{-2})	(1.91×10^{-2})	(1.91×10^{-2})	(2.10×10^{-2})	-
$MRD_t(\sigma^2D)$	2.44×10^{-1}	2.44×10^{-1}	2.44×10^{-1}	2.44×10^{-1}	2.44×10^{-1}	2.44×10^{-1}
	(1.43×10^{-1})	(1.43×10^{-1})	(1.43×10^{-1})	(1.43×10^{-1})	(1.43×10^{-1})	(1.43×10^{-1})

Table S4: Average MRD values for the β and σ^2D parameter estimates, taken relative to the baseline truth used for simulation (t) and the parameter estimates produced by lmer (l). All reported results were obtained through averages taken from 1000 simulations performed for each simulation setting, with parameter estimation performed using the ML likelihood criteria. Empirical standard errors are also given in brackets underneath each entry in the table.

S6 MRD results for ReML estimation

Method ($\mathbf{m_1}$)	FS	FFS	SFS	FSFS	CSFS	lmer
Simulation 1						
$MRD_l(\beta)$	2.55×10^{-5}	2.55×10^{-5}	1.93×10^{-5}	1.93×10^{-5}	4.32×10^{-5}	-
	(2.81×10^{-4})	(2.81×10^{-4})	(2.39×10^{-4})	(2.39×10^{-4})	(3.24×10^{-4})	-
$MRD_t(\beta)$	4.05×10^{-2}	4.05×10^{-2}	4.05×10^{-2}	4.05×10^{-2}	4.05×10^{-2}	4.05×10^{-2}
	(1.99×10^{-2})	(1.99×10^{-2})	(1.99×10^{-2})	(1.99×10^{-2})	(1.99×10^{-2})	(1.99×10^{-2})
$MRD_l(\sigma^2D)$	2.42×10^{-4}	2.42×10^{-4}	2.05×10^{-4}	2.05×10^{-4}	3.99×10^{-3}	-
	(1.89×10^{-3})	(1.89×10^{-3})	(1.78×10^{-3})	(1.78×10^{-3})	(8.35×10^{-2})	-
$MRD_t(\sigma^2D)$	2.42×10^{-1}	2.42×10^{-1}	2.42×10^{-1}	2.42×10^{-1}	2.42×10^{-1}	2.42×10^{-1}
	(1.27×10^{-1})	(1.27×10^{-1})	(1.27×10^{-1})	(1.27×10^{-1})	(1.27×10^{-1})	(1.27×10^{-1})
Simulation 2						
$MRD_l(\beta)$	1.08×10^{-4}	1.08×10^{-4}	7.74×10^{-5}	7.74×10^{-5}	7.45×10^{-5}	-
	(2.16×10^{-3})	(2.16×10^{-3})	(1.45×10^{-3})	(1.45×10^{-3})	(5.37×10^{-4})	-
$MRD_t(\beta)$	3.61×10^{-2}	3.61×10^{-2}	3.61×10^{-2}	3.61×10^{-2}	3.61×10^{-2}	3.61×10^{-2}
	(1.75×10^{-2})	(1.75×10^{-2})	(1.75×10^{-2})	(1.75×10^{-2})	(1.75×10^{-2})	(1.75×10^{-2})
$MRD_l(\sigma^2D)$	5.02×10^{-4}	5.02×10^{-4}	5.01×10^{-4}	5.01×10^{-4}	5.16×10^{-4}	-
	(2.35×10^{-3})	(2.35×10^{-3})	(2.35×10^{-3})	(2.35×10^{-3})	(2.30×10^{-3})	-
$MRD_t(\sigma^2D)$	3.27×10^{-1}	3.27×10^{-1}	3.27×10^{-1}	3.27×10^{-1}	3.27×10^{-1}	3.27×10^{-1}
	(1.86×10^{-1})	(1.86×10^{-1})	(1.86×10^{-1})	(1.86×10^{-1})	(1.86×10^{-1})	(1.86×10^{-1})
Simulation 3						
$MRD_l(\beta)$	1.22×10^{-4}	1.22×10^{-4}	1.22×10^{-4}	1.22×10^{-4}	5.45×10^{-3}	-
	(7.38×10^{-4})	(7.38×10^{-4})	(8.57×10^{-4})	(8.57×10^{-4})	(6.22×10^{-2})	-
$MRD_t(\beta)$	3.86×10^{-2}	3.86×10^{-2}	3.86×10^{-2}	3.86×10^{-2}	3.86×10^{-2}	3.86×10^{-2}
	(1.93×10^{-2})	(1.93×10^{-2})	(1.93×10^{-2})	(1.93×10^{-2})	(1.92×10^{-2})	(1.93×10^{-2})
$MRD_l(\sigma^2D)$	1.82×10^{-3}	1.82×10^{-3}	1.82×10^{-3}	1.82×10^{-3}	5.56×10^{-3}	-
	(1.14×10^{-2})	(1.14×10^{-2})	(1.14×10^{-2})	(1.14×10^{-2})	(1.65×10^{-2})	-
$MRD_t(\sigma^2D)$	2.54×10^{-1}	2.54×10^{-1}	2.54×10^{-1}	2.54×10^{-1}	2.54×10^{-1}	2.54×10^{-1}
	(1.53×10^{-1})	(1.53×10^{-1})	(1.53×10^{-1})	(1.53×10^{-1})	(1.53×10^{-1})	(1.53×10^{-1})

Table S5: Average MRD values for the β and σ^2D parameter estimates, taken relative to the baseline truth used for simulation (t) and the parameter estimates produced by lmer (l). All reported results were obtained through averages taken from 1000 simulations performed for each simulation setting, with parameter estimation performed using the ReML likelihood criteria. Empirical standard errors are also given in brackets underneath each entry in the table.

S7 ML likelihood criteria

Method	FS	FFS	SFS	FSFS	CSFS	lmer
Simulation 1						
$\hat{l}$ (Mean log-likelihood)	-1564.860	-1564.860	-1564.860	-1564.860	-1564.860	-1564.860
	(22.203)	(22.203)	(22.203)	(22.203)	(22.203)	(22.203)
$\hat{l}-\hat{l}_{lmer}$ (Mean difference from	0.030	0.030	0.031	0.031	-0.166	-
`lmer` log-likelihood, $\times 10^6$)	(0.306)	(0.306)	(0.306)	(0.306)	(0.318)	-
Simulation 2						
$\hat{l}$ (Mean log-likelihood)	-1683.238	-1683.238	-1683.238	-1683.238	-1683.238	-1683.238
	(22.598)	(22.598)	(22.598)	(22.598)	(22.598)	(22.598)
$\hat{l}-\hat{l}_{lmer}$ (Mean difference from	0.756	0.756	0.749	0.749	0.562	-
`lmer` log-likelihood, $\times 10^6$)	(12.977)	(12.977)	(12.977)	(12.977)	(12.975)	-
Simulation 3						
$\hat{l}$ (Mean log-likelihood)	-1918.952	-1918.952	-1918.952	-1918.952	-1918.974	-1918.952
	(22.925)	(22.925)	(22.925)	(22.925)	(22.916)	(22.925)
$\hat{l}-\hat{l}_{lmer}$ (Mean difference from	5.696	5.696	5.543	5.543	-2000.163	-
`lmer` log-likelihood, $\times 10^6$)	(50.795)	(50.795)	(50.793)	(50.793)	(22942.480)	-

Table S6: Maximized ML log-likelihood criteria averaged across the 1000 simulations performed for each simulation setting. Mean differences observed between each of the Fisher Scoring methods and lmer are reported on a scale of 10^{-6}. Underneath each entry in the table, empirical standard errors are displayed in brackets. All reported likelihoods can be seen to be indistinguishable from one another, apart from the CSFS method, which experienced convergence failure in a small number of instances.

S8 ReML likelihood criteria

Method	FS	FFS	SFS	FSFS	CSFS	lmer
Simulation 1						
$\hat{l}_R$ (Mean log-likelihood)	-1574.069	-1574.069	-1574.069	-1574.069	-1574.069	-1574.069
	(22.602)	(22.602)	(22.602)	(22.602)	(22.602)	(22.602)
$\hat{l}_R - \hat{l}_{R,lmer}$ (Mean difference from	0.024	0.024	0.024	0.024	-0.174	-
`lmer` log-likelihood, $\times 10^6$)	(0.327)	(0.327)	(0.326)	(0.326)	(0.336)	-
Simulation 2						
$\hat{l}_R$ (Mean log-likelihood)	-1694.464	-1694.464	-1694.464	-1694.464	-1694.464	-1694.464
	(22.721)	(22.721)	(22.721)	(22.721)	(22.721)	(22.721)
$\hat{l}_R - \hat{l}_{R,lmer}$ (Mean difference from	0.990	0.990	0.985	0.985	0.801	-
`lmer` log-likelihood, $\times 10^6$)	(9.905)	(9.905)	(9.903)	(9.903)	(9.909)	-
Simulation 3						
$\hat{l}_R$ (Mean log-likelihood)	-1930.486	-1930.486	-1930.486	-1930.486	-1930.530	-1930.486
	(22.460)	(22.460)	(22.460)	(22.460)	(22.468)	(22.460)
$\hat{l}_R - \hat{l}_{R,lmer}$ (Mean difference from	6.022	6.022	5.865	5.865	-43966.471	-
`lmer` log-likelihood, $\times 10^6$)	(43.037)	(43.037)	(43.037)	(43.037)	(345579.800)	-

Table S7: Maximized ReML log-likelihood criteria averaged across the 1000 simulations performed for each simulation setting. Mean differences observed between each of the Fisher Scoring methods and lmer are reported on a scale of 10^{-6}. Underneath each entry in the table, empirical standard errors are displayed in brackets. All reported likelihoods can be seen to be indistinguishable from one another, apart from the CSFS method, which experienced convergence failure in a small number of instances.

S9 The SAT score results

Estimation Method	FS	FFS	SFS	FSFS	CSFS	lmer
Fixed effects parameters						
β_0 (Intercept)	597.715	597.715	597.714	597.714	597.714	597.714
(Standard Error)	(7.534)	(7.534)	(7.535)	(7.535)	(7.535)	(7.535)
β_1 (Year)	28.556	28.556	28.557	28.557	28.557	28.557
(Standard Error)	(8.638)	(8.638)	(8.639)	(8.639)	(8.639)	(8.639)
Covariance parameters						
σ_i^2 (Student)	340.712	340.712	340.684	340.684	340.688	340.690
σ_j^2 (Teacher)	604.877	604.877	604.954	604.954	604.956	604.973
σ^2 (Residual)	237.943	237.973	237.972	237.972	237.966	237.962
Performance						
$\lvert l - l_{lmer} \rvert$ (Log-likelihood $\times 10^{-7}$)	0.313	0.313	3.321	3.321	1.933	–
t (Time in seconds)	0.216	0.176	0.200	0.175	0.282	0.178
n_{it} (Number of Iterations)	7	7	16	16	14	–

Table S8: Parameter estimates and performance metrics for the SAT score model. Provided are the results for the FS, FFS, SFS, FSFS and CSFS methods, as well as the results produced on the same dataset using lmer. All values in this table were obtained using ML, including the results of lmer which usually by default employs ReML. Log-likelihood values are given as absolute differences from lmer and reported on a scale of 10^{-7}.

S10 Index of notation

Notation	Definition	First introduced in
Y	Response vector.	Section 1.2.1, equation (1)
X	Fixed effects design matrix.	Section 1.2.1, equation (1)
β	Fixed effects parameter vector.	Section 1.2.1, equation (1)
Z	Random effects design matrix.	Section 1.2.1, equation (1)
b	Random effects vector.	Section 1.2.1, equation (1)
ε	Error term.	Section 1.2.1, equation (1)
σ^2	Fixed effects variance.	Section 1.2.1, equation (1)
I_n	Identity matrix of size $(n \times n)$.	Section 1.2.1, equation (1)
D	Random effects covariance matrix.	Section 1.2.1, equation (1)
Superscript $'$ (e.g. A')	Matrix transpose.	Section 1.2.2
n	Total number of observations.	Section 1.2.1
p	Total number of fixed effects.	Section 1.2.1
q	Second dimension of Z.	Section 1.2.1
θ	Shorthand for the parameters (β, σ^2, D).	Section 1.2.1, equation (2)
$l(\theta)$	The log-likelihood function of θ.	Section 1.2.1, equation (2)
e	The residual vector, $Y - X\beta$.	Section 1.2.1, equation (2)
V	The matrix defined as $I_n + ZDZ'$.	Section 1.2.1, equation (2)
r	Total number of factors.	Section 1.2.1
f_k	The kth factor.	Section 1.2.1
l_k	Number of levels (categories) into which the kth factor groups random effects.	Section 1.2.1
q_k	Number of random effects which are grouped by the kth factor.	Section 1.2.1
Subscript (k) (e.g. $A_{(k)}$)	The columns of a matrix which correspond to random effects grouped by the kth factor.	Section 1.2.1

Subscript (k, j) (e.g. $A_{(k,j)}$)	The columns of a matrix which correspond to random effects grouped into the jth level of the kth factor.	Section 1.2.1
D_k	The covariance matrix for the random effects grouped by the kth factor.	Section 1.2.1
$\oplus$	Direct sum.	Section 1.2.1
Hat notation. (e.g. $\hat{a}$)	Maximum likelihood (or restricted maximum likelihood) estimator. For example, $\hat{\beta}$ is the (restricted) maximum likelihood estimator of β.	Section 1.2.2
Subscript $[X,Y]$ (e.g. $A_{[X,Y]}$)	Row/column indexing. The matrix $A_{[X,Y]}$ represents the submatrix of A formed by the elements of A with row index $x \in X$ and the column index. $y \in Y$	Section 1.2.2
vec	Vectorization.	Section 1.2.2
vech	Half-vectorization.	Section 1.2.2
N_k	The unique matrix of dimension $(k^2 \times k^2)$ satisfying $\text{vec}(A) = N_k\text{vec}(A + A')/2$ for any arbitrary square matrix A of dimension $(k \times k)$.	Section 1.2.2
$K_{m,n}$	Commutation matrix. I.e. the unique $(mn \times mn)$ matrix which satisfies $\text{vec}(A) = K_{m,n}\text{vec}(A')$ for any arbitrary matrix A of dimension $(m \times n)$.	Section 1.2.2
$\mathscr{D}_k$	Duplication Matrix. I.e. the unique $(k^2 \times k(k+1)/2)$ matrix satisfying $\text{vec}(A) = \mathscr{D}_k\text{vech}(A)$ for any arbitrary symmetric matrix A of dimension $(k \times k)$.	Section 1.2.2
$\mathscr{L}_k$	Elimination matrix. I.e. the unique $(k(k+1)/2 \times k^2)$ matrix satisfying $\text{vech}(A) = \mathscr{L}_k\text{vec}(A)$ for any arbitrary lower triangular matrix A of dimension $(k \times k)$.	Section 1.2.2
Subscript s (e.g. θ_s)	Iteration number. This subscript is only employed in Fisher Scoring update rules where its meaning is clear.	Section 2.1, equation (3)
α	Scalar step size.	Section 2.1, equation (3)
d (e.g. $\frac{dY}{dX}$)	Total derivative operator. I.e. the matrix derivative operator which accounts for dependencies between elements of a matrix. For example, d accounts for the equality between reflected elements of a symmetric matrix (see Supplementary Material S12.1).	Section 2.1, equation (3)
E (e.g. $E(X)$)	Expectation.	Section 2.1
Superscript $+$ (e.g. A^+)	Moore-Penrose inverse.	Section 2.1, equation (4)

$\theta^h, \theta^f, \theta^c$	Half, full and Cholesky representations of θ, respectively.	Section 2.1
Λ_k	The Cholesky factor of D_k (I.e. $\Lambda_k\Lambda_k' = D_k$).	Section 2.1
$\mathscr{I}^{h(f/c)}_{a,b}$,$\mathscr{I}^{h(f/c)}_{a}$	Sub-matrix of the Fisher Information matrix. The superscript h, f or c specifies the half, full or Cholesky representations, respectively. The subscript represents the parameter vectors a and b, to which the sub-matrix corresponds. The notation $\mathscr{I}^{h(f/c)}_{a}$ is shorthand for $\mathscr{I}^{h(f/c)}_{a,a}$.	Section 2.1
$\mathbf{0}_{m,n}$	Matrix of zeros with dimensions $(m \times n)$.	Section 2.1.1, equation (9)
$\otimes$	Kronecker product.	Section 2.1.1, equation (10)
∂ (e.g. $\frac{\partial X}{\partial Y}$)	Partial derivative operator. I.e. the matrix derivative operator which does not account for dependencies between elements of a matrix. For example, ∂ does not account for the equality between reflected elements of a symmetric matrix (see Supplementary Material S12.1).	Section 2.1.2, equation (11)
$F(\theta^f)$	The matrix used in the place of the Fisher Information matrix for the Pseudo-Inverse Fisher Scoring approach.	Section 2.1.2, equation (14)
$F_{a,b}, F_a$	$F_{a,b}$ is the sub-matrix of F corresponding to parameter vectors a and b. F_a is shorthand for $F_{a,a}$.	Section 2.1.2, equation (15)
$\beta_0, \sigma_0^2, D_{k,0}$	Initial estimates for β, σ^2 and D_k, respectively.	Section 2.2, equations (22), (23)
P,Q,R,S,T,U	The product forms $X'X, X'Y, X'Z, Y'Y, Y'Z$ and $Z'Z$, respectively.	Section 2.3
vec_m	Generalized vectorization.	Section 2.3
$\tilde{A}$	For an arbitrary matrix, A, the tilde matrix, $\tilde{A}$, is defined by the mapping given by equation (25). Notation only employed in Section 2.3.	Section 2.3, equation (25)
vecu	Unique vectorisation. I.e. $\text{vecu}(A)$ is a column vector of the unique variables required to specify A, listed in column major order.	Section 2.4
θ^{con}	Constrained representation of θ.	Section 2.4
$\mathscr{C}_k$	Constraint matrix for the kth random factor.	Section 2.4, equation (27)

$\mathscr{I}^{con}$	Fisher Information matrix for constrained representation of θ.	Section 2.4
ρ_D	A set of parameters which, when taken together, can be used to specify all non-zero elements of D_k.	Section 2.4
$\mathscr{C}$	Constraint matrix modelling constraints for all random factors in the LMM.	Section 2.4
T	T-statistic. Notation only employed in section 2.5.	Section 2.5
L	Contrast vector of dimension $(1 \times p)$	Section 2.5
v	Degrees of freedom of T-statistic.	Section 2.5
Var	Variance.	Section 2.5, equation (28)
η	Shorthand for the parameters (σ^2, D).	Section 2.5, equation (28)
$S^2(\hat{\eta})$	The estimated variance of $L\hat{\beta}$, $\widehat{\text{Var}}(L\hat{\beta})$, as a function of the variance estimates, $\hat{\eta}$.	Section 2.5, equation (28)
$v(\hat{\eta})$	Satterthwaite estimated degrees of freedom, based on the variance parameter estimates, $\hat{\eta}$.	Section 2.5, equation (28)
η^h	Half-representation of η.	Section 2.5
$\hat{B}$	The matrix $Z'\hat{V}^{-1}X(X'\hat{V}^{-1}X)^{-1}L'$.	Section 2.5
$\sigma_a^2, \sigma_c^2, \sigma_e^2$	The additive genetic, common environmental and residual error variances in the ACE model. σ_e^2 is equivalent to the notation σ^2 employed in previous sections.	Section 4.1.2
$\mathbf{K}_k^a, \mathbf{K}_k^c$	Kinship matrices for additive genetic and common environmental variance components, respectively (see see Supplementary Material S16.1).	Section 4.1.2

Table S9: Index of notation. Listed above are all notational conventions used throughout the paper, "Fisher Scoring for crossed factor Linear Mixed Models". Notations which are introduced in the appendices are not included.

S11 Ensuring non-negative definiteness

In order to ensure numerical optimization produces a valid covariance matrix when performing LMM parameter estimation, optimization must be constrained to ensure that for each factor, f_k, D_k is non-negative definite. This is a well-documented obstacle for numerical approaches to LMM parameter estimation for which many solutions have been suggested. In this section, we describe how non-negative definiteness was ensured for the covariance matrix estimates produced by the Fisher Scoring algorithms described in the main text.

A common approach, utilized in Bates et al. [2015] for example, is to reparameterize optimization in terms of the Cholesky factor of the covariance matrix, Λ_k, as oppose to the covariance matrix itself, D_k. This approach is adopted by the CSFS method, described in Section 2.1.5. However, the methods described in Sections 2.1.1-2.1.4 require an alternative approach as optimization does not account for the constraint that D_k must be non-negative definite. For the FS, FFS, SFS and FSFS algorithms, we use an approach described by Demidenko [2013], for the single factor LMM. Denoting the Eigendecomposition of $\{D_k\}_{k\in\{1,\dots,r\}}$ as $D_k = U_k\Sigma_k U_k'$, we project D_k onto the space of all $(q_k \times q_k)$ non-negative definite matrices by, following each update of the covariance matrix, D, replacing $\{D_k\}_{k\in\{1,..r\}}$ with $\{D_{+,k}\}_{k\in\{1,..r\}}$,

$$D_{+,k} = U_k\Sigma_{+,k}U_k',$$

where $\Sigma_{+,k} = \max(\Sigma_k, 0)$ with 'max' representing the element-wise maximum. For each factor, f_k, this ensures D_k, and as a result D, is non-negative definite.

We note here it could be argued that using the Eigendecomposition in this manner defeats our objective to employ only simplistic vectorizable operations for parameter estimation. In response to this potential criticism, however, we highlight that vectorized Eigendecomposition operations are widely available in practice and can be found in many programming languages such as, for example, MatLab and Python.

S12 Matrices and differentiation

In this section, supplementary information is provided for the material found in Appendix 6.1 of the main text. Section S12.1 provides an explicit definition for, and discussion of, the notion of derivative employed throughout the main text. Section S12.2 restates and proves the lemmas employed in the arguments of Appendix 6.1.

S12.1 Derivative definition

Many different notions of matrix derivative currently exist in the statistical literature. In this section, we explicitly state the definition of derivative which is employed throughout "Fisher Scoring for crossed factor Linear Mixed Models". Throughout this work, for arbitrary matrices A and B of dimensions $(p \times q)$ and $(m \times n)$ respectively, the following definition of matrix (partial) derivative has been implicitly employed:

$$\frac{\partial \text{vec}(A)}{\partial \text{vec}(B)} = \begin{bmatrix} \frac{\partial A_{[1,1]}}{\partial B_{[1,1]}} & \cdots & \frac{\partial A_{[p,1]}}{\partial B_{[1,1]}} & \cdots & \frac{\partial A_{[p,q]}}{\partial B_{[1,1]}} \\ \vdots & \ddots & \vdots & \ddots & \vdots \\ \frac{\partial A_{[1,1]}}{\partial B_{[m,1]}} & \cdots & \frac{\partial A_{[p,1]}}{\partial B_{[m,1]}} & \cdots & \frac{\partial A_{[p,q]}}{\partial B_{[m,1]}} \\ \vdots & \ddots & \vdots & \ddots & \vdots \\ \frac{\partial A_{[1,1]}}{\partial B_{[m,n]}} & \cdots & \frac{\partial A_{[p,1]}}{\partial B_{[m,n]}} & \cdots & \frac{\partial A_{[p,q]}}{\partial B_{[m,n]}} \end{bmatrix},$$

with the equivalent definition holding for the total derivative. In addition, for a scalar value, a, and matrix, B, defined as above, we employ the below matrix (partial) derivative definition:

$$\frac{\partial a}{\partial B} = \begin{bmatrix} \frac{\partial a}{\partial B_{[1,1]}} & \cdots & \frac{\partial a}{\partial B_{[1,n]}} \\ \vdots & \ddots & \vdots \\ \frac{\partial a}{\partial B_{[m,1]}} & \cdots & \frac{\partial a}{\partial B_{[m,n]}} \end{bmatrix},$$

with, again, the equivalent definition holding for the total derivative. The above notation can be useful since by definition, the two derivatives defined above satisfy the below relation:

$$\text{vec}\left(\frac{\partial a}{\partial B}\right) = \frac{\partial a}{\partial \text{vec}(B)}.$$

The matrix derivative definitions we have used are commonly employed in the mathematical and statistical literature, but are not a universal standard. For example, sources such as Neudecker and Wansbeek [1983] and Magnus and Neudecker [1999] define $\partial \text{vec}(A)/\partial \text{vec}(B)$ as the transpose of the definition supplied above. This discrepancy between definitions is noteworthy as some of the referenced results used in the appendices of "Fisher Scoring for crossed factor Linear Mixed Models" are the transpose of those found in the original source. For a full account and in-depth discussion of the different definitions of matrix derivatives used in the literature, we recommend the work of Turkington [2013].

S12.2 Derivative lemmas

In this Section, Lemma 1 and Lemma 2, which were employed in Section 6.1 of the main text, are restated as Lemma S1 and Lemma S2 and proven rigorously.

Lemma S1. *Let g be a column vector, A be a square matrix and $\{B_s\}$ be a set of arbitrary matrices of equal size. Let K be an unstructured matrix which none of g, A or any of the $\{B_s\}$ depend on. Further, assume $A, \{B_s\}, g$ and K can*

be multiplied as required. The below matrix derivative expression now holds;

$$\frac{\partial}{\partial K}\left[g'(A+\sum_t B_tKB_t')^{-1}g\right] =$$
$$-\sum_s B_s'\big(A'+\sum_t B_tKB_t'\big)^{-1}gg'\big(A'+\sum_t B_tKB_t'\big)^{-1}B_s. \tag{S1}$$

Proof. To begin, denote $M = A+\sum_s B_sKB_s'$. For clarity and convenience, in this proof, we shall briefly depart from the usual notation of $H_{[i,j]}$ for the $(i,j)^{th}$ element of an arbitrary matrix H and instead employ Ricci calculus notation, in which the $(i,j)^{th}$ element of H is given by H^i_j and all summations are made implicit. The left hand side of (S1) is now given, using Ricci calculus notation, as;

$$\frac{\partial}{\partial K^m_n}\left[(g')_i(M^{-1})^i_jg^j\right] = (g')_ig^j\frac{\partial}{\partial K^m_n}\left[M^{-1}\right]^i_j. \tag{S2}$$

We employ a result from Giles [2008] which states, in Ricci calculus notation;

$$\frac{\partial[F^{-1}]^i_j}{\partial x} = -(F^{-1})^i_q\frac{\partial F^q_p}{\partial x}(F^{-1})^p_j.$$

Applying this result to (S2), it can be seen that the right hand side of (S2) is equal to:

$$-(g')_ig^j(M^{-1})^i_q\frac{\partial M^q_p}{\partial K^m_n}(M^{-1})^p_j.$$

Evaluating the derivative in the above expression and rearranging the terms gives the below:

$$\begin{aligned}&-(B_s')^n_p(M^{-1})^p_jg^j(g')_i(M^{-1})^i_qB^q_{sm}\\ &=-[B_s'M^{-1}gg'M^{-1}B_s]^n_m.\end{aligned}$$

By applying a transposition to the matrices in the above expression, in order to interchange the indices m and n, the result follows. □

Lemma S2. *Let $A,\{B_s\}$ and K be defined as in Lemma S1. Then the following is true:*

$$\frac{\partial}{\partial K}\log|A+\sum_t B_tKB_t'| = \sum_s B_s'(A+\sum_t B_tK'B_t')^{-1}B_s. \tag{S3}$$

Proof. As in the proof of Lemma S1, the notation of $M = A+\sum_t B_tK'B_t'$ will be adopted and the proof will be given in Ricci calculus notation. Expanding the derivative with respect to K^m_n using the partial derivatives of the elements of M gives the following:

$$\frac{\partial}{\partial K^m_n}\log|M| = \frac{\partial\log|M|}{\partial M^i_j}\frac{\partial M^i_j}{\partial K^m_n}. \tag{S4}$$

We now make use of the following well-known result, which can be found in Giles [2008].

$$\frac{\partial\log|X|}{\partial X^i_j} = (X'^{-1})^i_j. \tag{S5}$$

Inserting (S5) into (S4) and noting that A^i_j does not depend on K^m_n, yields:

$$\frac{\partial}{\partial K^m_n}\log|M| = (M'^{-1})^i_j\frac{\partial(B_sK(B_s)')^i_j}{\partial K^m_n}.$$

Evaluating the derivative on the right hand side of the above now returns:

$$\frac{\partial}{\partial K_n^m} \log|M| = (M'^{-1})^i_j (B_s)^i_m (B'_s)^n_j = (B'_s)^m_i (M'^{-1})^i_j (B_s)^j_n.$$

Combining the summation terms in the above expression yields the following.

$$\frac{\partial}{\partial K_n^m} \log|M| = (B'_s M'^{-1} B_s)^m_n.$$

Finally, converting the above into matrix notation now gives (S3) as desired. □

S13 Full Fisher Scoring

In this section, we prove that equations (14) and (18) in the main text are valid Fisher Scoring rules of the form given by equation (4). To achieve this, we first require the following lemma.

Lemma S3. *Let n, p and k be arbitrary positive integers with $p < n$. Let $\tilde{N}$ be of dimension $(n \times n)$ with rank$(\tilde{N}) < n$, $\tilde{K}$ be of dimension $(n \times p)$ with rank$(\tilde{K}) = p$, C be of dimension $(n \times n)$ with rank$(\tilde{N}) = n$ and δ be of size $(n \times k)$. If the following properties are satisfied:*

1. *$\tilde{K}\tilde{K}^{+} = \tilde{N}$, where $\tilde{K}^{+} = (\tilde{K}'\tilde{K})^{-1}\tilde{K}'$ is the generalized inverse of $\tilde{K}$.*
2. *$\tilde{N}$ is symmetric and idempotent.*
3. *$\tilde{N}C = C\tilde{N} = \tilde{N}C\tilde{N}$.*
4. *$\tilde{N}\delta = \delta$.*

then it follows that:

$$(\tilde{N}C)^{+}\delta = C^{-1}\delta.$$

Proof. In proving the above lemma, we make use of the following result given by Barnett [1990];

- If A is a matrix of dimension $(m \times l)$ with rank$(A) = l < m$, and B is a matrix of dimension $(l \times m)$ with rank$(B) = l$, then;

$$(AB)^{+} = B'(BB')^{-1}(A'A)^{-1}A'. \tag{S6}$$

By property 1, we have that:

$$(\tilde{N}C)^{+} = (\tilde{K}\tilde{K}^{+}C)^{+}. \tag{S7}$$

By applying (S6) to the above, with $A = \tilde{K}$ and $B = \tilde{K}^{+}C$, and simplifying the result, the following is obtained:

$$(\tilde{K}\tilde{K}^{+}C)^{+} = C'\tilde{K}^{+'}(\tilde{K}^{+}CC'\tilde{K}^{+'})^{-1}\tilde{K}^{+}. \tag{S8}$$

We now consider the inversion in the center of the above expression, i.e. the inversion of $(\tilde{K}^{+}CC'\tilde{K}^{+'})$. This inversion is given by;

$$(\tilde{K}^{+}CC'\tilde{K}^{+'})^{-1} = \tilde{K}'C^{-1'}C^{-1}\tilde{K}.$$

We will demonstrate this by showing that the product of the matrix inside the inversion on the left hand side and the matrix on the right hand side is the identity matrix:

$$(\tilde{K}^{+}CC'\tilde{K}^{+'})(\tilde{K}'C^{-1'}C^{-1}\tilde{K}) = \tilde{K}^{+}CC'\tilde{K}^{+'}\tilde{K}'C^{-1'}C^{-1}\tilde{K}.$$

Using properties 1, 2 and 3 we see that the above is equal to:

$$\tilde{K}^{+}\tilde{N}CC'C^{-1'}C^{-1}\tilde{K}.$$

Simplifying and applying property 1 now yields that the above is equal the below:

$$\tilde{K}^{+}\tilde{N}\tilde{K} = \tilde{K}^{+}\tilde{K} = I,$$

where I is the identity matrix as required. Returning to (S8) and applying properties 1 and 2, we now have that:

$$(\tilde{N}C)^{+} = C'\tilde{N}C^{-1'}C^{-1}\tilde{N}.$$

By applying properties 2 and 3, the above can now be reduced to:

$$(\tilde{N}C)^{+} = \tilde{N}C^{-1}\tilde{N}. \tag{S9}$$

We now note that, by property 3, $\tilde{N}C = C\tilde{N}$. By multiplying the left and right side of this identity by C^{-1} it can be seen that:

$$C^{-1}\tilde{N} = \tilde{N}C^{-1}.$$

Multiplying the right hand side of the above by $\tilde{N}$ and applying property 2 we obtain:

$$C^{-1}\tilde{N} = \tilde{N}C^{-1}\tilde{N}.$$

Substituting the above into (S9) gives:

$$(\tilde{N}C)^{+} = C^{-1}\tilde{N}.$$

The result of Lemma S3 now follows by multiplying the right-hand side by δ and applying property 4.

□

Theorem S1. *The below two update rules are equivalent, where $F(\theta_s^f)$ is the matrix defined in Section 2.1.2 and $\mathscr{I}(\theta_s^f)$ is the Fisher Information matrix of θ_s^f.*

$$\theta_{s+1}^f = \theta_s^f + \alpha_s F(\theta_s^f)^{-1}\frac{\partial l(\theta_s^f)}{\partial \theta},$$

$$\theta_{s+1}^f = \theta_s^f + \alpha_s \mathscr{I}(\theta_s^f)^{+}\frac{\partial l(\theta_s^f)}{\partial \theta}.$$

Proof. To prove Theorem S1, it suffices to prove the below equality:

$$\mathscr{I}(\theta_s^f)^{+}\frac{\partial l(\theta_s^f)}{\partial \theta} = F(\theta_s^f)^{-1}\frac{\partial l(\theta_s^f)}{\partial \theta}.$$

To prove the above, we employ Lemma S3. To achieve this, we proceed by defining:

- $\tilde{N}$ to be the matrix:

$$\begin{bmatrix} I_{p+1} & 0 & 0 & \dots & 0 \\ 0 & N_{q_1} & 0 & \dots & 0 \\ 0 & 0 & N_{q_2} & \dots & 0 \\ \vdots & \vdots & \vdots & \ddots & \vdots \\ 0 & 0 & 0 & \dots & N_{q_r} \end{bmatrix},$$

- $\tilde{K}$ to be the matrix:

$$\begin{bmatrix} I_{p+1} & 0 & 0 & \dots & 0 \\ 0 & K_{q_1,q_1} & 0 & \dots & 0 \\ 0 & 0 & K_{q_2,q_2} & \dots & 0 \\ \vdots & \vdots & \vdots & \ddots & \vdots \\ 0 & 0 & 0 & \dots & K_{q_r,q_r} \end{bmatrix},$$

- δ to be the column vector:

$$\frac{\partial l(\theta^f)}{\partial \theta^f},$$

- C to be the matrix $F(\theta^f)$,

and claim that $F(\theta^f)\tilde{N} = \mathscr{I}(\theta^f)$. To prove this equality, we first note the following two properties of the matrix N_k, given by Magnus and Neudecker [1986].

1. $N_{k_1}(A \otimes A) = N_{k_1}(A \otimes A)N_{k_2} = (A \otimes A)N_{k_2}$ for any arbitrary matrix A and scalars k_1 and k_2, such that the matrix multiplications are well defined.

2. $N_k\text{vec}(A) = \text{vec}(A)$ for any arbitrary symmetric matrix A of appropriate dimension.

The first of the above properties, in combination with equations (13) and (15), can be seen to imply that, for any k_1 and k_2 between 1 and r:

$$\mathscr{I}^f_{\text{vec}(D_{k_1}),\text{vec}(D_{k_2})} = F_{\text{vec}(D_{k_1}),\text{vec}(D_{k_2})}N_{q_{k_2}}.$$

In a similar manner, the second of the above properties, in combination with equation (12), can be seen to imply that, for any k between 1 and r:

$$\mathscr{I}^f_{\sigma^2,\text{vec}(D_k)} = F_{\sigma^2,\text{vec}(D_k)}N_{q_k}.$$

Expanding the multiplication $F(\theta^f_s)\tilde{N}$ block-wise demonstrates that the two equations above are sufficient to prove the equality $F(\theta^f_s)\tilde{N} = \mathscr{I}(\theta^f_s)$. The matrices $\tilde{N}, \tilde{K}$ and C can be seen to satisfy properties 1-3 of Lemma S3 by noting standard results concerning the matrices $\mathscr{D}_k$, $K_{m,n}$ and N_n, provided in Magnus and Neudecker [1986]. Property 4 of Lemma S3 can be seen to hold for the matrix $\tilde{N}$ and column vector δ by noting the symmetry of $\frac{\partial l(\theta^f_s)}{\partial D_k}$ (see Theorem 1). By combining the previous arguments and applying Lemma S3, the below is obtained:

$$\mathscr{I}(\theta^f)^+\delta = (F(\theta^f)\tilde{N})^+\delta = F(\theta^f)^{-1}\delta.$$

The result of Theorem S1 now follows.

□

Theorem S2. *For any fixed integer k between* 1 *and r, the below two update rules are equivalent, where $F_{vec(D_{k,s})}$ is as defined in Section 2.1.2 and $\mathscr{I}^f_{vec(D_{k,s})}$ is the Fisher Information matrix of $vec(D_k)$.*

$$vec(D_{k,s+1}) = vec(D_{k,s}) + \alpha_s F^{-1}_{vec(D_{k,s})}\frac{\partial l(\theta^f_s)}{\partial vec(D_{k,s})},$$

$$vec(D_{k,s+1}) = vec(D_{k,s}) + \alpha_s\big(\mathscr{I}^f_{vec(D_{k,s})}\big)^+\frac{\partial l(\theta^f_s)}{\partial vec(D_{k,s})}.$$

Proof. The proof of the above proceeds by defining $\tilde{N} = N_{q_k}$, $\tilde{K} = K_{q_k,q_k}$, $C = F_{\text{vec}(D_k)}$ and δ to be the partial derivative appearing in the statement of the theorem. Following this, the proof follows the same structure as that of Theorem S1. □

S14 Constraint Matrices

In this section, we provide further detail on the approach to constrained optimization which was outlined in Section 2.4 of the main text. We begin by providing examples of how the notation described in Section 2.4 may be used in practice. For a given k, we introduce the notation $\{d_i\}$ and $\{\tilde{d}_i\}$ to represent the set of unique parameters required to specify the constrained and unconstrained representations of D_k, respectively. For example, if D_k is (3×3) in dimension and we wish to induce Toeplitz structure onto D_k, then D_k can be considered to take the following "constrained" and "unconstrained" representations:

$$D_k = \begin{bmatrix} d_1 & d_4 & d_7 \\ d_2 & d_5 & d_8 \\ d_3 & d_6 & d_9 \end{bmatrix} = \begin{bmatrix} \tilde{d}_1 & \tilde{d}_2 & \tilde{d}_3 \\ \tilde{d}_2 & \tilde{d}_1 & \tilde{d}_2 \\ \tilde{d}_3 & \tilde{d}_2 & \tilde{d}_1 \end{bmatrix}.$$

The notation $\text{vecu}(A)$ is used to denote the column vector of unique variables in the matrix A, given in order of first occurrence as listed in column major order. In the above example, this implies $\text{vecu}(D_k) = [\tilde{d}_1, \tilde{d}_2, \tilde{d}_3]'$ whilst $\text{vec}(D_k) = [d_1, \dots, d_9]'$. Using this notation, the constraint matrix $\mathscr{C}_k$ may be defined element-wise. To see this, note that the chain rule for the total derivative is given by:

$$\frac{dl(\theta^{con})}{d\tilde{d}_j} = \sum_i \frac{\partial d_i}{\partial \tilde{d}_j} \frac{\partial l(\theta^{con})}{\partial d_i}.$$

Comparing this with the derivative expression provided in equation (27), it can be seen that the elements of $\mathscr{C}_k$ are given by:

$$\mathscr{C}_{k[j,i]} = \frac{\partial d_i}{\partial \tilde{d}_j}.$$

In general, derivation of the constraint matrix for the kth factor is relatively straightforward for most practical applications. Table S10 supports this statement by providing examples of the constraint matrix for the kth factor operating under the assumption that D_k exhibits several commonly employed covariance structures. In addition, we note that two instances in which a constraint matrix approach was employed implicitly can be found in the main body of this work. In Section 2.1.1, we took $\mathscr{C}_k = \mathscr{D}_{q_k}$ to enforce symmetry in D_k and, in Section 2.1.5, we took $\mathscr{C}_k = (d\text{vech}(D_k)/d\text{vech}(\Lambda_k))\mathscr{D}_{q_k}$ in order to derive the Fisher Scoring update in terms of the Cholesky factor, Λ_k.

It is also worth noting that this approach to constrained optimization allows for different covariance structures to be enforced on different factors in the LMM. For instance, a researcher may enforce the first factor in an LMM to have an AR(1) structure while allowing the second factor to exhibit a completely different structure such as compound symmetry. As noted in Section 2.4, this approach can also be extended further to allow all elements of the vectorized covariance matrices, $\text{vec}(D_1), \dots \text{vec}(D_r)$, to be expressed as functions of one set of parameters, ρ_D, common to all $\{\text{vec}(D_k)\}_{k \in \{1,\dots,r\}}$. To further detail this, we define $v(D)$ and extend the definition θ^{con}:

$$v(D) = \begin{bmatrix} \text{vec}(D_1) \\ \vdots \\ \text{vec}(D_r) \end{bmatrix}, \qquad \theta^{con} = \begin{bmatrix} \beta \\ \sigma^2 \\ \rho_D \end{bmatrix},$$

Through the same process used to obtain equation the equations presented in Section 2.4, the below can be obtained;

$$\begin{aligned} \frac{dl(\theta^{con})}{d\rho_D} &= \mathscr{C} \frac{\partial l(\theta^{con})}{\partial v(D)}, \\ \mathscr{I}^{con}_{\rho_D} &= \mathscr{C} \mathscr{I}^f_{\text{v}(D)} \mathscr{C}', \end{aligned} \tag{S10}$$

where $\mathscr{C}$ is the constraint matrix defined to be the Jacobian matrix of partial derivatives of $v(D)$ taken with respect to ρ_D. The score vector and Fisher Information matrix associated to $v(D)$ are readily seen to be given by block-wise combinations of the matrix expressions (11) and (13).

**Covariance Structure**	**Constraint matrix, $\mathscr{C}_k$**
Diagonal $\begin{bmatrix} \tilde{d}_1 & 0 & 0 \\ 0 & \tilde{d}_1 & 0 \\ 0 & 0 & \tilde{d}_1 \end{bmatrix}$	$\begin{bmatrix} 1 & 0 & 0 & 0 & 1 & 0 & 0 & 0 & 1 \end{bmatrix}$
Variance components $\begin{bmatrix} \tilde{d}_1 & 0 & 0 \\ 0 & \tilde{d}_2 & 0 \\ 0 & 0 & \tilde{d}_3 \end{bmatrix}$	$\begin{bmatrix} 1 & 0 & 0 & 0 & 0 & 0 & 0 & 0 & 0 \\ 0 & 0 & 0 & 0 & 1 & 0 & 0 & 0 & 0 \\ 0 & 0 & 0 & 0 & 0 & 0 & 0 & 0 & 1 \end{bmatrix}$
Toeplitz $\begin{bmatrix} \tilde{d}_1 & \tilde{d}_2 & \tilde{d}_3 \\ \tilde{d}_2 & \tilde{d}_1 & \tilde{d}_2 \\ \tilde{d}_3 & \tilde{d}_2 & \tilde{d}_1 \end{bmatrix}$	$\begin{bmatrix} 1 & 0 & 0 & 0 & 1 & 0 & 0 & 0 & 1 \\ 0 & 1 & 0 & 1 & 0 & 1 & 0 & 1 & 0 \\ 0 & 0 & 1 & 0 & 0 & 0 & 1 & 0 & 0 \end{bmatrix}$
Compound Symmetry $\begin{bmatrix} 1 & \tilde{d}_1 & \tilde{d}_1 \\ \tilde{d}_1 & 1 & \tilde{d}_1 \\ \tilde{d}_1 & \tilde{d}_1 & 1 \end{bmatrix}$	$\begin{bmatrix} 0 & 1 & 1 & 1 & 0 & 1 & 1 & 1 & 0 \end{bmatrix}$
AR(1) $\begin{bmatrix} 1 & \tilde{d}_1 & \tilde{d}_1^2 \\ \tilde{d}_1 & 1 & \tilde{d}_1 \\ \tilde{d}_1^2 & \tilde{d}_1 & 1 \end{bmatrix}$	$\begin{bmatrix} 0 & 1 & 2\tilde{d}_1 & 1 & 0 & 1 & 2\tilde{d}_1 & 1 & 0 \end{bmatrix}$

Table S10: The constraint matrix for the kth factor, $\mathscr{C}_k$, in the setting in which the kth factor groups 3 random effects, under various covariance structures.

S15 Satterthwaite degrees of freedom estimation

In this section, we provide an in-depth accounting of Satterthwaite estimation for degrees of freedom in the multi-factor LMM setting. In Section S15.1, we detail how the expression described by equation (28) of the main text is derived for estimating the degrees of freedom of the approximate T-statistic. Following this, in Section S15.2, we describe how this approach is extended to degrees of freedom estimation for the approximate F-statistic. These sections follow the proofs outlined in the work of Kuznetsova et al. [2017]. The reader is referred to this work for a more in-depth discussion of Satterthwaite degrees of freedom estimation for the LMM.

S15.1 Satterthwaite estimation for the approximate T-statistic

To derive the estimator given by (28), we first begin by noting that the approximate T-statistic used for LMM null hypothesis testing is given by:

$$T = \frac{L\hat{\beta}}{\sqrt{L\widehat{\text{Var}}(\hat{\beta})L'}},$$

where $\hat{\beta}$ is the estimator of β obtained from numerical optimization and $\widehat{\text{Var}}(\hat{\beta})$ is an estimate of the variance of the β estimator, given by:

$$\widehat{\text{Var}}(\hat{\beta}) = \hat{\sigma}^2(X'\hat{V}^{-1}X)^{-1}.$$

We highlight that the above expression differs from the T-statistic typically employed in the setting of linear regression hypothesis testing. Under the standard assumptions for linear regression, it is possible to derive an exact expression for the distribution of the OLS variance estimate of $\hat{\beta}$ given by $\hat{\sigma}^2(X'X)^{-1}$. However, the above estimator, $\widehat{\text{Var}}(\hat{\beta})$, differs from the OLS estimate as it includes an estimate of V which has an unknown distribution. As a direct result, no equivalent theory is available to obtain the distribution of $\widehat{\text{Var}}(\hat{\beta})$ in the setting of the LMM. A consequence of this is that, whilst T follows a student's t_{n-p} distribution in the setting of linear regression, the distribution of T in the LMM setting is unknown. The aim of this section is to approximate the degrees of freedom of T assuming that it follows a students t-distribution.

To meet this aim, we now denote $\text{Var}(\hat{\beta})$ as the unknown true variance of the $\hat{\beta}$ estimator. By multiplying both the numerator and denominator of the T-statistic by $(L\text{Var}(\hat{\beta})L)^{1/2}$, the below expression for T is obtained:

$$T = \frac{Z}{\sqrt{\frac{L\widehat{\text{Var}}(\hat{\beta})L'}{L\text{Var}(\hat{\beta})L'}}},$$

where Z is a random variable with standard normal distribution, given by $Z = L\hat{\beta}/\sqrt{L\text{Var}(\hat{\beta})L'}$. We now consider the definition of the students t-distribution, which states that a random variable is t-distributed with v degrees of freedom if and only if it takes the following form:

$$T = \frac{Z}{\sqrt{\frac{X}{v}}},$$

where $Z \sim N(0,1)$ and $X \sim \chi^2_v$. When the degrees of freedom v are unknown, as both X and T are distributed with v degrees of freedom, it suffices to estimate the degrees of freedom of the chi-square variable X instead of the t-distributed variable T. By equating the approximate T-statistic with the defining form of a t-distributed random variable, under the assumption that the T-statistic is truly t-distributed, the below expression for X can be obtained:

$$X = \frac{vL\widehat{\text{Var}}(\hat{\beta})L'}{L\text{Var}(\hat{\beta})L'} \sim \chi^2_v.$$

We now define the notation $S^2(\hat{\eta})$ to represent the estimated variance of $L\hat{\beta}$ as a function of the estimated variance parameters given by $\hat{\eta} = (\hat{\sigma}^2, \hat{D}_1, \dots, \hat{D}_r)$. Similarly, we define Σ^2 to be the true unknown variance of $L\hat{\beta}$. Noting that $\Sigma^2 = L\text{Var}(\hat{\beta})L'$ and by rearranging the above, the below can be obtained:

$$S^2(\hat{\eta}) = L\widehat{\text{Var}}(\hat{\beta})L' \sim \frac{\Sigma^2}{v}\chi^2_v.$$

By considering the variance of the above expression and noting that, as $X \sim \chi^2_v$, $\text{Var}(X) = 2v$, the below expression is obtained.

$$\text{Var}(S^2(\hat{\eta})) = \frac{(\Sigma^2)^2}{v^2}\text{Var}(X) = \frac{2(\Sigma^2)^2}{v}.$$

To obtain an estimator of v in terms of $S^2(\hat{\eta})$, the above is rearranged and the approximation $\Sigma^2 \approx S^2(\hat{\eta})$ is used. This yields the below approximation.

$$\hat{v}(\hat{\eta}) = \frac{2(S^2(\hat{\eta}))^2}{\text{Var}(S^2(\hat{\eta}))}.$$

Under the assumption that $T \sim t_v$, $\hat{v}(\hat{\eta})$ is an estimator for the degrees of freedom of X and, therefore, for the degrees of freedom of T. This concludes the derivation of (28).

S15.2 Satterthwaite estimation for the approximate F-statistic

In this section, we detail how the Satterthwaite method described in Section S15.1 may be extended in order to estimate the degrees of freedom of the approximate F-statistic. The approximate F-statistic for the LMM takes the below form:

$$F = \frac{\hat{\beta}'L'[L\widehat{\text{Var}}(\hat{\beta})L']^{-1}L\hat{\beta}}{r},$$

where, throughout this section only, the notation F will represent the approximate F-statistic and r will be used to denote $r = \text{rank}(L)$. The aim of this section is to approximate F with an $F_{r,v}$ distribution where v, the denominator degrees of freedom, is estimated using a Satterthwaite method of approximation. To simplify the notation we will first consider Q, which is the numerator of F;

$$Q = rF = \hat{\beta}'L'[L\text{Var}(\hat{\beta})L']^{-1}L\hat{\beta}.$$

We begin by considering the eigendecomposition of $L\text{Var}(\hat{\beta})L'$, which we will denote:

$$L\text{Var}(\hat{\beta})L' = U\Lambda U',$$

where U is an orthonormal matrix of eigenvectors and Λ is a diagonal matrix of eigenvalues. Using standard properties of the eigendecomposition the below is obtained:

$$\begin{aligned} Q &= (U'L\hat{\beta})'\Lambda^{-1}(U'L\hat{\beta}) \\ &= \sum_{i=1}^{r} \frac{(U'L\hat{\beta})^2_i}{\lambda_i}, \end{aligned}$$

where $(U'L\hat{\beta})_i$ is the ith element of the vector $U'L\hat{\beta}$ and λ_i is the ith diagonal element (eigenvalue) of Λ, $\Lambda_{[i,i]}$. For purposes which will become clear, we define $\tilde{L}$ as $U'L$. By noting that $\lambda_i = (U'L\text{Var}(\hat{\beta})L'U)_i$, the above can be seen to be equal to:

$$Q = \sum_{i=1}^{r} \left(\frac{\tilde{L}_i\hat{\beta}}{\sqrt{\tilde{L}_i\text{Var}(\hat{\beta})\tilde{L}'_i}} \right)^2,$$

where $\tilde{L}_i$ represents the ith row of $\tilde{L}$. Noting now the definition of $\tilde{L}$, it can be seen that the above is a sum of independent squared T-statistics of the form discussed in Section S15.1. By denoting the unknown degrees of freedom of each of the T-statistics in the above sum by v_i, it follows that:

$$\begin{aligned}\mathbb{E}(Q) &= \sum_{i=1}^{r} \mathbb{E}\left[\left(\frac{\tilde{L}_i\hat{\beta}}{\sqrt{\tilde{L}_i \text{Var}(\hat{\beta})\tilde{L}_i'}}\right)^2\right] \\ &= \sum_{i=1}^{r} \frac{v_i}{v_i - 2},\end{aligned}$$

where the second equality follows from the fact that a squared T-statistic with degrees of freedom v_i is an F_{1,v_i}-statistic, which in turn has expectation $\frac{v_i}{v_i-2}$. Noting that, in practice, the true variance of $\hat{\beta}$ is unknown, the methods of S15.1 must be employed to estimate each of the v_i in the above sum.

To use the above expression for $\mathbb{E}(Q)$ to estimate the denominator degrees of freedom of F, three cases are considered; the cases $r > 2, r = 2$ and $r = 1$. Each of these will now be considered in turn.

Case 1 (r $>$ 2): By recalling $Q = rF$, and by the standard formula for the expectation of an F distribution with numerator degrees of freedom v greater than 2, the below is obtained.

$$\mathbb{E}(Q) = \mathbb{E}(rF) = \frac{rv}{v-2}.$$

Setting the above equal to the previous expression for $\mathbb{E}(Q)$ and rearranging, the below expression for v is obtained:

$$v = \frac{2\sum_{i=1}^{r} \frac{v_i}{v_i-2}}{\left(\sum_{i=1}^{r} \frac{v_i}{v_i-2}\right) - r}.$$

By using the Satterthwaite degrees of freedom approximation method described in Section S15.1 to estimate $\{v_i\}_{i\in\{1,\dots,r\}}$, it follows that the above formula can be used to estimate the degrees of freedom of the approximate F-statistic when $r > 2$.

Case 2 (r $=$ 2): When $r = 2$, the expectation of Q is infinite and, resultantly, the equality used in the argument of case 1 no longer holds. A common convention for estimation of v in the case $r = 2$ is based on the observation that for $r > 2$ the expectation of Q satisfies the following relationship with v:

$$v = \frac{2\mathbb{E}(Q)}{\mathbb{E}(Q) - r}.$$

By taking the limit from above of both sides of this expression, and noting that $\mathbb{E}(Q) \to \infty$ as $v \downarrow 2$, the following estimate for v is obtained.

$$v = \lim_{r \downarrow 2} \frac{2\mathbb{E}(Q)}{\mathbb{E}(Q) - r} = 2.$$

Case 3 (r $=$ 1): For the case $r = 1$, it is well known that if $F \sim F_{1,v_1}$ for some integer v_1, then F is the square of a T-statistic with the same degrees of freedom, v_1. Resultantly, in this case, the unknown denominator degrees of freedom of the F-statistic, v_1, can be estimated using the methods of Section S15.1.

S16 The ACE model

This section provides additional material concerning the ACE model described in Section 4.1.2 of the main text. For readers unfamiliar with the ACE model, Section S16.1 provides a brief overview of the kinship and environmental matrices that appear in the model specification. For readers interested in computational efficiency, Section S16.2 provides detail on how the FSFS algorithm of Section 2.1.4, ReML adjustments of Appendix 6.3 and constrained optimization approach of Section 2.4 may be efficiently combined in order to perform parameter estimation for the ACE model.

S16.1 Kinship and environmental matrices

In twin studies, twin pairs are typically described as either Monozygotic (MZ) or Dizygotic (DZ). MZ twin pairs are identical twins, whilst DZ twins are non-identical. The additive genetic kinship matrix, $\mathbf{K}^a_k$, describes the additive genetic similarity between members of a family unit. For a family of q_k members, $\mathbf{K}^a_k$, is a $(q_k \times q_k)$ dimensional constant matrix with its $(i,j)^{th}$ element given by:

$$(\mathbf{K}^a_k)_{[i,j]} = \begin{cases} 1 & \text{if subjects } i \text{ and } j \text{ are MZ twins or } i=j. \\ \frac{1}{2} & \text{if subjects } i \text{ and } j \text{ are DZ twins or full siblings.} \\ \frac{1}{4} & \text{if subjects } i \text{ and } j \text{ are half siblings.} \\ 0 & \text{if subjects } i \text{ and } j \text{ are unrelated.} \end{cases}$$

The common environmental matrix, $\mathbf{K}^c_k$, describes the similarity between members of a family unit which is attributed to individuals' being reared in a shared environment. $\mathbf{K}^c_k$ is a $(q_k \times q_k)$ dimensional constant matrix with its $(i,j)^{th}$ element given by:

$$(\mathbf{K}^a_k)_{[i,j]} = \begin{cases} 1 & \text{if subjects } i \text{ and } j \text{ were reared together.} \\ 0 & \text{if subjects } i \text{ and } j \text{ were not reared together.} \end{cases}$$

For more information on the construction of kinship and common environmental matrices for twin studies, the reader is referred to Lawlor et al. [2009].

S16.2 Computation and the ACE model

A key aspect in which the ACE model differs from other LMM's considered in this work is that, in the ACE model, the second dimension of the random effects design matrix, q, is equal to the number of observations, n. Resultantly, the random effects design matrix, Z, which is given by the $(n \times n)$ identity matrix, has notably large dimensions. Due to the large size of Z, the methods of Section 2.3, which assume that the second dimension of Z is much smaller than n (i.e. $q << n$), cannot be applied to the ACE model in order to improve computation speed. In this section, we briefly outline how the FSFS algorithm of Section 2.1.4 may be implemented efficiently, in conjunction with the ReML adjustments described in Appendix 6.3 and the constrained optimization methods of Section 2.4 to perform parameter estimation for the ACE model.

To begin, we consider the GLS updates, given by equation (16), which are employed for updating the parameters β and σ^2. By defining the matrix, $\bar{D}_k$ by $\bar{D}_k = I_{q_k} + D_k$, and noting that the matrix Z is the $(n \times n)$ identity matrix, the below matrix equality can be seen to hold for the ACE model:

$$X'V^{-1}X = \sum_{k=1}^{r} \sum_{j=1}^{l_k} X'_{(k,j)} \bar{D}_k^{-1} X_{(k,j)},$$

where $X_{(k,j)}$ represents the design matrix for the jth family unit which possesses family structure of type k. Via well-known properties of the Kronecker product, the above equality can be seen to be equivalent to the following:

$$\text{vec}(X'V^{-1}X) = \sum_{k=1}^{r} \left(\sum_{j=1}^{l_k} X'_{(k,j)} \otimes X'_{(k,j)} \right) \text{vec}(\bar{D}_k^{-1}).$$

The above equality is noteworthy as the matrix expression which appears inside the large brackets on the right-hand side is fixed and does not depend on β, σ^2 or D. Further, the matrix expression inside the brackets is typically small in size and has dimensions $(p^2 \times q_k^2)$. As a result, this expression can be calculated and stored prior to the iterative procedure of the FSFS algorithm. This means that the above equality offers a quick and convenient method for repeated evaluation of the matrix, $X'V^{-1}X$, which is employed for many calculations throughout the FSFS algorithm. Similar logic can be applied to the evaluation of the vector, $X'V^{-1}Y$, and the scalar value, $Y'V^{-1}Y$. Using this approach, it can now be seen that the GLS estimators, (16), can be calculated using only $\{D_k\}_{k\in\{1,\dots,r\}}$ and pre-calculated matrices which are small in dimension. Since the matrices $\{D_k\}_{k\in\{1,\dots,r\}}$ are also typically small in dimension, this calculation is extremely quick and computationally efficient.

Next, we consider the matrix $F_{\text{vec}(D_k)}$ and the partial derivative vector of $l_R(\theta^f)$, taken with respect to $\text{vec}(D_k)$. Expressions for these quantities can be obtained using equations (15) and (11), respectively, in combination with the adjustments described in Appendix 6.3. Again noting that the random effects design matrix, Z, is equal to the identity matrix, the ReML equivalent of equation (11) simplifies substantially to the below expression:

$$\frac{\partial l_R(\theta^f)}{\partial \text{vec}(D_k)} = \frac{1}{2}\text{vec}\left(\bar{D}_k^{-1}\left(\frac{E_k E_k'}{\sigma^2} - l_k \bar{D}_k + \sum_{j=1}^{l_k} X_{(k,j)}(X'V^{-1}X)^{-1}X'_{(k,j)}\right)\bar{D}_k^{-1}\right),$$

where $E_k = \text{vec}_{q_k}(e'_{(k)})'$, vec_m is defined as the generalized vectorization operator described in Section 2.3 and $e_{(k)}$ is defined as the residual vector corresponding to subjects who belong to a family unit with family structure type k. Using equation (15), the sub-matrix of F corresponding to $\text{vec}(D_k)$ can also be seen to be given by the following simplified expression.

$$F_{\text{vec}(D_k)} = \frac{1}{2} l_k (\bar{D}_k^{-1} \otimes \tilde{D}_k^{-1}).$$

The two expressions above can be evaluated computationally in an extremely efficient manner primarily for two reasons. The first of these is that the matrices involved in computation are typically small; D_k, E_k and $X_{(k,j)}$ have dimensions $(q_k \times q_k)$, $(q_k \times l_k q_k)$ and $(q_k \times p)$, respectively. The second reason is that, excluding the inversions of $\bar{D}_k$ and $X'V^{-1}X$, the only operations required to evaluate the above expressions are computationally quick to perform. These operations are: matrix multiplication, the reshape operation, matrix addition and the Kronecker product.

Lastly, we consider the evaluation of the score vector and Fisher Information matrix associated to $\tau = [\tau_a, \tau_c]' = \sigma_e^{-1}[\sigma_a, \sigma_c]'$, which shall be denoted as $\frac{dl(\theta^{ACE})}{d\tau}$ and $\mathscr{I}_\tau^{ACE}$, respectively. We define $\mathbf{K}_k = [\text{vec}(\mathbf{K}_k^a), \text{vec}(\mathbf{K}_k^c)]'$. By employing equation (S10) of Section S14, and using the constraint matrix derived in Appendix 6.6.2, the score vector and Fisher Information matrix associated to τ can be reduced to the following:

$$\mathscr{I}_\tau^{ACE} = (\tau\tau') \odot \left(\sum_{k=1}^{r} \mathbf{K}_k F_{\text{vec}(D_k)} \mathbf{K}_k'\right),$$
$$\frac{dl_R(\theta^{ACE})}{d\tau} = \tau \odot \left(\sum_{k=1}^{r} \mathbf{K}_k \frac{\partial l_R(\theta^f)}{\partial \text{vec}(D_k)}\right),$$

where $\odot$ represents the Hadamard (entry-wise) product. The results of Section 4.2.2 were produced by using the GLS updates for the parameters β and σ^2 and by employing the two expressions above to perform Fisher Scoring update steps for the parameter vector τ.

It should be noted that, in the ACE model, misspecification of variance components can result in a low-rank Fisher Information matrix. This can be seen by noting the positioning of the Hadamard product in the above Fisher Information matrix expression and by considering the case in which one of the elements of τ equals 0. If not accounted for appropriately, this issue may, in practice, result in failure of the algorithm to converge. A simple, practical solution to this issue is to execute the algorithm multiple times using different initial starting points; once with a starting point where τ_a is set to zero, once with τ_c set to zero and once with neither τ_a nor τ_c set to zero. The results of Section 4.2.2 were obtained using this approach.

References

Douglas Bates, Martin Mächler, Ben Bolker, and Steve Walker. Fitting linear mixed-effects models using lme4. *Journal of Statistical Software, Articles*, 67(1):1–48, 2015. ISSN 1548-7660. doi: 10.18637/jss.v067.i01.

E. Demidenko. *Mixed Models: Theory and Applications with R*. Wiley Series in Probability and Statistics. Wiley, 2013. ISBN 9781118592991. URL `https://books.google.co.uk/books?id=uSmRAAAAQBAJ`.

H. Neudecker and Tom Wansbeek. Some results on commutation matrices, with statistical applications. *Canadian Journal of Statistics*, 11(3):221–231, 1983. doi: 10.2307/3314625.

Jan R. Magnus and Heinz Neudecker. *Matrix differential calculus with applications in statistics and econometrics*. Wiley Series in Probability and Statistics. John Wiley, rev. ed edition, 1999. ISBN 9780471986324,0471986321,047198633X.

Darrell A. Turkington. *Generalized Vectorization, Cross-Products, and Matrix Calculus*. Cambridge University Press, 2013. doi: 10.1017/CBO9781139424400.

Mike B. Giles. Collected matrix derivative results for forward and reverse mode algorithmic differentiation. pages 35–44, 2008.

S. Barnett. *Matrices: Methods and Applications*. Oxford applied mathematics and computing science series. Clarendon Press, 1990. ISBN 9780198596806.

Jan R. Magnus and Heinz Neudecker. Symmetry, 0-1 matrices, and jacobians : a review. *Econometric Theory*, page 46, 1986.

Alexandra Kuznetsova, Per Brockhoff, and Rune Christensen. lmertest package: Tests in linear mixed effects models. *Journal of Statistical Software, Articles*, 82(13):1–26, 2017. ISSN 1548-7660. doi: 10.18637/jss.v082.i13. URL `https://www.jstatsoft.org/v082/i13`.

D.A. Lawlor, D.A. Lawlor, and G.D. Mishra. *Family Matters: Designing, Analysing and Understanding Family Based Studies in Life Course Epidemiology*. Life Course Approach to Adult Health. OUP Oxford, 2009. ISBN 9780199231034.
